# Effect of Treatment on Steroidome in Women with Multiple Sclerosis

**DOI:** 10.3390/ijms26051835

**Published:** 2025-02-20

**Authors:** Martin Hill, Radmila Kancheva, Marta Velíková, Ludmila Kančeva, Josef Včelák, Radek Ampapa, Michal Židó, Ivana Štětkářová, Jana Libertínová, Michala Vosátková, Jana Vítků, Lucie Kolátorová, Tereza Škodová, Eva Kubala Havrdová

**Affiliations:** 1 Institute of Endocrinology, 110 00 Prague, Czech Republic; mvelikova@endo.cz (M.V.); lkancheva@endo.cz (L.K.); jvcelak@endo.cz (J.V.); mvosatkova@endo.cz (M.V.); jvitku@endo.cz (J.V.); lkolatorova@endo.cz (L.K.); tskodova@endo.cz (T.Š.); 2 MS Center, Jihlava Hospital, 586 01 Jihlava, Czech Republic; ampapar@gmail.com; 3 Department of Neurology 3FM CU and UHKV, Third Faculty of Medicine, Charles University, 100 34 Prague, Czech Republic; michal.zido@fnkv.cz (M.Ž.); ivana.stetkarova@fnkv.cz (I.Š.); 4 MS Center, Second Faculty of Medicine, Charles University, 150 06 Prague, Czech Republic; jana.libertinova@fnmotol.cz; 5 Department of Neurology, First Faculty of Medicine, Charles University, 128 21 Prague, Czech Republic; ehavr@lf1.cuni.cz

**Keywords:** multiple sclerosis, steroidomics, GC-MS/MS, multivariate statistics, anti-MS drugs

## Abstract

Multiple sclerosis (MS) is a chronic inflammatory neurodegenerative disease of the central nervous system. The manifestation of MS is related to steroid changes during the menstrual cycle and pregnancy. As data focusing on the effect of anti-MS drug treatment on steroidome are scarce, we evaluated steroidomic changes (79 steroids) in 61 female MS patients of reproductive age 39 (29, 47) years (median with quartiles) after treatment with anti-MS drugs on the GC-MS/MS platform and immunoassays (cortisol and estradiol). The changes were assessed using steroid levels and steroid molar ratios (SMRs) that may reflect the activities of steroidogenic enzymes (SMRs). A repeated measures ANOVA, followed by multiple comparisons and OPLS models, were used for statistical analyses. The anti-MS treatment decreased steroid levels in the follicular phase. Anti-CD20 monoclonal antibodies (mAb), such as ofatumumab and ocrelizumab; inhibitors of the sphingosine-1-phosphate receptor (S1PRI); and IFNβ-1a decreased circulating 17-hydroxy-pregnanes and shifted the CYP17A1 functioning from the hydroxylase- toward the lyase step. Decreased conjugated/unconjugated steroid ratios were found after treatment with anti-MS drugs, especially for glatiramer acetate and anti-CD20 mAb. In the luteal phase, IFN-β1a treatment increased steroidogenesis; both IFN-β1a and ocrelizumab increased AKR1D1, and S1PRI increased SRD5A functioning. Anti-CD20 mAb reduced the functioning of enzymes catalyzing the synthesis of immunomodulatory 7α/β and 16α-hydroxy-androgens, which may affect the severity of MS. The above findings may be important concerning the alterations in bioactive steroids, such as cortisol; active androgens and estrogens; and neuroactive, neuroprotective, and immunomodulatory steroids in terms of optimization of anti-MS treatment.

## 1. Introduction

Multiple sclerosis (MS) is a chronic inflammatory disease of the central nervous system (CNS) that causes demyelination and neurodegeneration [1]. MS is associated with varying degrees of inflammation, with the inflammatory reaction being promoted by the secretion of autoantigens, and is therefore an autoimmune inflammatory response. This hypothesis suggests that primary degeneration is present several years before the first overt clinical symptoms and progresses over the course of the disease (see review [2]).

### 1.1. Multiple Sclerosis and Steroids

Hormones play a key role in the physiology and pathology of the immune system, specifically in adaptive immunity. Exacerbation of MS is closely related to the menstrual cycle and occurs at very low levels of sex steroids [3]. In addition, MS symptoms decrease in the last three months of pregnancy and increase after delivery, probably due to hormonal and immunological changes [4].

#### 1.1.1. Cortisol

Chronic inflammation is associated with altered hypothalamic pituitary adrenal axis (HPAA) in patients with autoimmune diseases (see review [5]). The most important endogenous steroid involved in the regulation of HPAA, cortisol, is a potent endogenous anti-inflammatory hormone that is released into the circulation from the *zona fasciculata* of the adrenal cortex after physical and mental stimuli. With the loss of adequate response to stress, HPAA does not produce sufficient quantities of this glucocorticoid [5,6]. The genomic effects of cortisol are mediated by binding to high-affinity mineralocorticoid receptors (MR) and low-affinity glucocorticoid receptors, whereas the non-genomic effects of cortisol are mediated by membrane receptors and G-protein coupled signaling [6].

Circulating cortisol, which is governed by circadian rhythms and peaks early in the morning, suppresses levels of several pro-inflammatory cytokines, including IL-2, IL-3, IL-6, TNF, and interferon (IFN), and also affects the activity and survival of immune cells (see review [5]). In addition, cortisol binds to the GC receptor on T cell mitochondria and disrupts their function. While low cortisol levels induce activation of macrophages, high concentrations have an immunosuppressive effect (see review [5]).

Furthermore, cortisol affects the activity of several neurotransmitter systems that influence reward processing, attention regulation, executive function, mood, and emotion, such as type A GABA receptors (GABA_A_Rs), glutamate, dopamine, and acetylcholine receptors. Cortisol inhibits the synthesis and secretion of serotonin, which increases the risk of depression [6] and also affects attention, perception, memory, and emotions. Finally, chronically elevated cortisol levels can lead to cognitive impairment [7,8,9].

#### 1.1.2. Δ^5^ Steroids

While the adrenal *zona fasciculata* produces all the cortisol circulating in the body, the adrenal *zona reticularis* produces approximately 80% of dehydroepiandrosterone (DHEA), and the testes, ovaries, and brain synthesize the rest. Although adrenocorticotropic hormone (ACTH) stimulates both cortisol and DHEA and its sulfate (DHEAC) synthesis, the feedback mechanisms typical for cortisol are absent in DHEA/C [6]. In contrast to the diabetogenic effect of cortisol, DHEAC is an anti-glucocorticoid that protects the hippocampus from the damaging effect of cortisol [7,9,10,11].

The formation of another neuroprotective Δ^5^ steroid pregnenolone (Preg) is catalyzed by cholesterol desmolase, which activity is rate-limiting for adrenal steroidogenesis [6]. Preg, DHEA, Preg sulfate (PregC), and DHEAC cross the blood-brain barrier (BBB), and their adrenal production affects their levels in the CNS [12,13,14].

The aforementioned steroids protect from glutamate-induced neurotoxicity, stabilize microtubules, activate neurite growth, and promote myelination [15,16] and may also improve cognitive function and counteract pain transmission and fear via modulation of various ionotropic receptors (see [7,17]).

DHEA/DHEAC has anti-inflammatory effects reducing the levels of interleukin 1 (IL-1), interleukin 4 (IL-4) and interleukin 6 (IL-6), interleukin 12 (IL-12), and tumor necrosis factor (TNF) [16]. Interestingly, higher TNF levels, which are typical for autoimmune diseases, inhibit the conversion from the DHEAC to the DHEA, which may induce disequilibrium between anti- and pro-inflammatory factors (see review [5]).

#### 1.1.3. Active Androgens

Androgens play a positive role in the development and function of the innate immune response by inhibiting the adaptive immune system, thereby preventing autoimmunity [1]. Lower androgen levels were observed in some autoimmune diseases, and the protective effects of androgens were attributed to suppression of DCs activation, decreased secretion of type I IFNγ, diminished secretion of pro-inflammatory cytokines, and reduced T helper 1 (Th1) T cell response. Moreover, androgens suppress B cell differentiation and antibody production and stimulate T helper 2 (Th2) pathway and IL-10 secretion (see review [5]).

#### 1.1.4. Progesterone Metabolites

The P metabolite allopregnanolone (3α,5α-THP) has multiple neuroprotective effects, including alleviation of neurobehavioral deficits and attenuation of neuropathology and inflammation [18,19].

### 1.2. Effect of Anti-MS Drugs on Circulating Steroids and Steroid Molar Ratios (SMRs)

Data in the literature focusing on the effect of steroid treatment on anti-MS are rare, but a few relevant reports were found [20,21,22,23,24,25].

#### 1.2.1. Glatiramer Acetate (GA)

GA is an immunomodulatory drug used for the treatment of relapsing-remitting MS (RRMS). GA blocks peptide-major histocompatibility complex and exerts its anti-inflammatory effect by affecting antigen-presenting cells (APCs) to promote the development of Th2 and production of anti-inflammatory cytokines (IL-4, IL-13, and IL-10) and, consequently, suppressing the development of pathogenic T cells (Th1 and Th17) [26,27]. As to the effect of GA therapy on steroidome, Cil et al. did not find changes in follitropin (FSH) and E2 levels in MS patients treated by combined therapy with interferon β and GA but found diminished ovarian volume in MS patients compared to age-matched controls [20].

#### 1.2.2. Interferons β1a (IFN-β1a)

Interferon β-1a (IFN-β1a), which is produced by mammalian cells, is a cytokine used for MS treatment. IFN-β1a balances pro- and anti-inflammatory cytokines and primarily decreases pro-inflammatory Th17 cells [27,28,29]. Griffiths and Wang [21] proposed that cholesterol 25-hydroxylase (CH25H) is an interferon-stimulated gene and 25-hydroxycholesterol can be considered as an immunoregulatory oxysterol that affects the adaptive immune system.

#### 1.2.3. Inhibitors of the Sphingosine-1-Phosphate Receptor (S1PRI)

S1PRI decreases T and B cells, activates S1P signaling in CNS, and reduces Th1 cytokines [27,28]. E2 rapidly stimulates sphingosine kinase activity in endothelial cells (EC), promoting sphingosine-1-phosphate (S1P) production in EC. Decreased S1P1R levels impair estrogen-induced EC migration and tube formation [22]. S1P rapidly increases cortisol biosynthesis and the expression of genes participating in cholesterol uptake and transport in H295R adrenocortical cells [23]. Luteotropin (LH) and FSH promote S1P synthesis in follicular cells via activation of protein kinase C, while S1P mediates FSH and LH proliferation, survival, and viability in follicular cells [24].

#### 1.2.4. Ofatumumab (Kesimpta) and Ocrelizumab (Ocrevus)

Ofatumumab is a humanized anti-CD20 mAb developed as an immunosuppressive drug for the treatment of MS [30]. Regarding ofatumumab, there is no information on its effect on the steroidome to date. Ocrelizumab is another humanized anti-CD20 mAb frequently used for the treatment of RRMS [31] and primary progressive multiple sclerosis (PPMS) [32]. In a recent study, suppression of the production of various androstane and pregnane steroids by ocrelizumab was reported [25].

## 2. Results

### 2.1. Pooled Trends of Changes in Circulating Steroid Levels and Steroid Molar Ratios (SMRs) After Treatment with Anti-MS Drugs

The significance of the changes after the anti-MS treatment (stage or treatment effect) were evaluated for stage factor in ANOVA model (before treatment vs. after treatment) and for least significant difference (LSD) multiple comparisons separately for follicular (FP) and luteal (LP) phases of the menstrual cycle (PMCs). The pooled steroidomic changes (without differentiation of individual anti-MS drugs) after treatment are shown in Table S1. The results of the Wilcoxon one-sample test with correction for continuity, which was used to assess the prevailing trend of changes in steroid levels or SMRs in MS patients after treatment with anti-MS drugs, are summarized in Table 1, separately for FP and LP.

Generally, significant trends in steroidomic changes after anti-MS treatment are obvious in the FP; changes in LP are mostly insignificant, with the exception of a borderline trend to lower values of SMRs that may reflect the activity of CYP11B1. Therefore, the following results apply to FP. For a total of 79 investigated steroids, a significant trend toward a decrease in their levels after treatment was found. A borderline trend to decrease after treatment was observed for unconjugated steroids, but this trend reached significance for steroid conjugates. Regarding the groups of steroids, while the C21 steroids (pregnanes) showed a significant downward trend after treatment, the C19 steroids (androstanes) showed only an insignificant tendency in this direction. Also, 17α-hydroxy-pregnanes tended to decrease after treatment, but their 17-deoxy-counterparts did not. Furthermore, the 20α-dihydro-pregnanes showed a borderline trend to decrease in FP, but their 20-oxo counterparts did not. Finally, the GABAergic 3α-hydroxy-5α/β-steroids showed a significant downward trend after treatment; however, this was not the case for the 3-oxo-/3β -hydroxy-5α/β-steroids.

#### 2.1.1. Corticoids (C21 Δ^4^ Steroids) and 11β-Hydroxy-Androstanes (C19 Δ^4^ and 5α/β Steroids)

**Figure 1:**
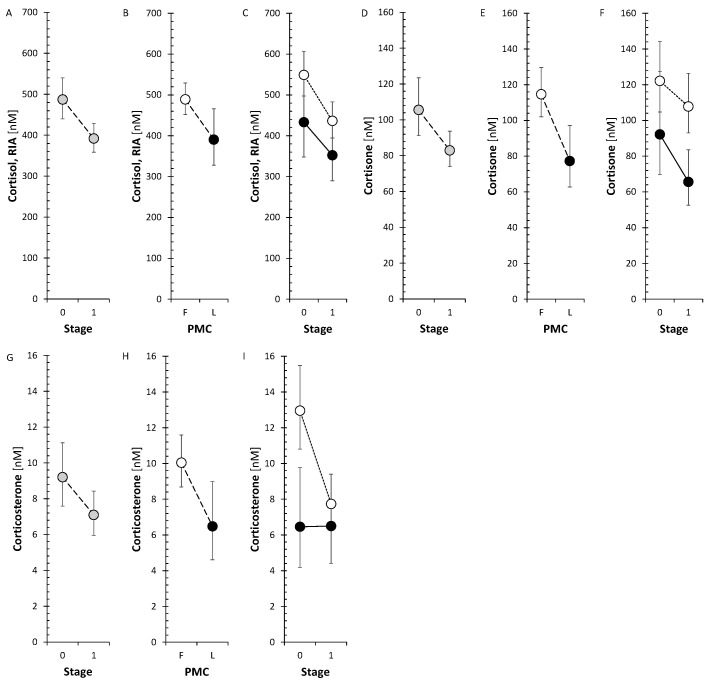
Changes in cortisol (measured by RIA) and cortisone levels (measured by GC-MS/MS) in patients with MS after treatment with anti-MS drugs. White, black, and grey circles with error bars represent the retransformed means with 95% confidence intervals for follicular menstrual phase, luteal menstrual phase, and changes in main factor stage of ANOVA model consisting of subject factor (Subj), effects of treatment (Stage) and menstrual phase (PMC), and Stage × PMC interaction. Panels (**A**–**C**) show the effects of Stage, PMC, and Stage × PMC interactions for cortisol, panels (**D**–**F**) for cortisone, and panels (**G**–**I**) for corticosterone. The symbols F, *p*, and η_p_^2^ represent the F-statistics, *p*-values, and effect size for factors and interaction. The statistics for factors and interaction are as follows: Stage: F = 6.6, *p* = 0.013, η_p_^2^ = 0.11; PMC: F = 1.9, *p* = 0.171, η_p_^2^ = 0.0351; Stage × PMC: F = 0, *p* = 0.925, η_p_^2^ = 0.000167; Subj: F = 2.1, *p* = 0.004, η_p_^2^ = 0.7 for cortisol (measured by RIA), Stage: F = 4, *p* = 0.051, η_p_^2^ = 0.0702; PMC: F = 3.3, *p* = 0.074, η_p_^2^ = 0.059; Stage × PMC: F = 0.9, *p* = 0.351, η_p_^2^ = 0.0164; Subj: F = 1.5, *p* = 0.07, η_p_^2^ = 0.621 for cortisone and Stage: F = 2.5, *p* = 0.12, η_p_^2^ = 0.0442; PMC: F = 2.2, *p* = 0.144, η_p_^2^ = 0.0392; Stage × PMC: F = 2, *p* = 0.16, η_p_^2^ = 0.0363; Subj: F = 2.5, *p* < 0.001, η_p_^2^ = 0.725. Non-overlapping error bars indicate a significant difference (*p* < 0.05, least significant multiple comparisons).

The corticoids and 11β-hydroxy-androstanes tended to a borderline decrease after treatment in FP but not in LP. Specifically, cortisol measured by both GC-MS/MS and radioimmunoassay (RIA) showed a significant decrease in FP, like cortisone and corticosterone. The changes in circulating cortisol, cortisone, and corticosterone are also illustrated in Figure 1.

#### 2.1.2. Δ^5^ and Δ^4^ Steroids

**Figure 2:**
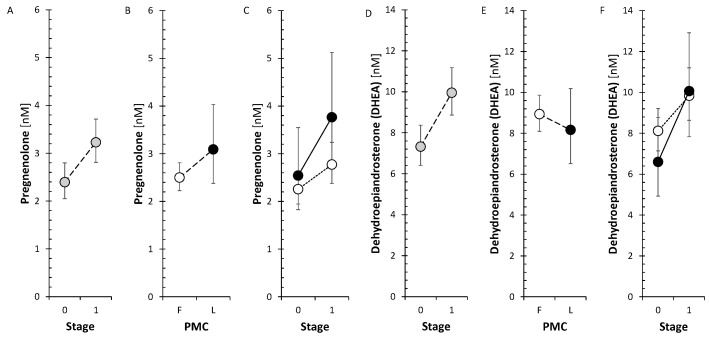
Changes of pregnenolone and DHEA levels in patients with MS after treatment with anti-MS drugs. The drawings and symbols are the same as for Figure 1. The statistics for factors and interaction are as follows: Panel (**A**): Stage: F = 5.1, *p* = 0.028, η_p_^2^ = 0.0848; Panel (**B**): PMC: F = 0.8, *p* = 0.376, η_p_^2^ = 0.0143; Panel (**C**): Stage × PMC: F = 0.4, *p* = 0.543, η_p_^2^ = 0.00675; Subj: F = 2.3, *p* = 0.001, η_p_^2^ = 0.709 for pregnenolone and Panel (**D**): Stage: F = 7.5, *p* = 0.009, η_p_^2^ = 0.119; Panel (**E**): PMC: F = 0.2, *p* = 0.656, η_p_^2^ = 0.00364; Panel (**F**): Stage × PMC: F = 0.8, *p* = 0.374, η_p_^2^ = 0.0144; Subj: F = 2.8, *p* < 0.001, η_p_^2^ = 0.751 for DHEA.

Changes after treatment showed no consistent significant trend for either Δ^5^ steroids or 11-deoxy Δ^4^ steroids. Nevertheless, the neuroprotective steroids, pregnenolone and DHEA, significantly increased when evaluating the factor stage in ANOVA model (Figure 2).

**Figure 3:**
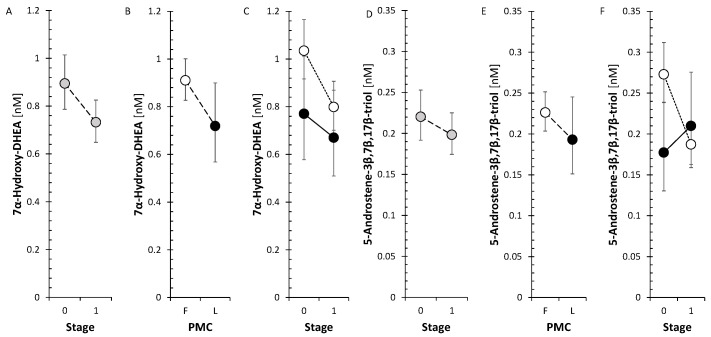
Changes of 7α-hydroxy-DHEA and 5-androstene-3β,7β,17β-triol levels in patients with MS after treatment with anti-MS drugs. The drawings and symbols are the same as for Figure 1. The statistics for factors and interaction are as follows: Panel (**A**): Stage: F = 3.3, *p* = 0.074, η_p_^2^ = 0.0578; Panel (**B**): PMC: F = 1.4, *p* = 0.249, η_p_^2^ = 0.0245; Panel (**C**): Stage × PMC: F = 0.3, *p* = 0.602, η_p_^2^ = 0.00506; Subj: F = 4, *p* < 0.001, η_p_^2^ = 0.809 for 7α-hydroxy-DHEA and Panel (**D**): Stage: F = 0.8, *p* = 0.377, η_p_^2^ = 0.0148; Panel (**E**): PMC: F = 0.5, *p* = 0.468, η_p_^2^ = 0.00998; Panel (**F**): Stage × PMC: F = 4.2, *p* = 0.045, η_p_^2^ = 0.0736; Subj: F = 3.6, *p* < 0.001, η_p_^2^ = 0.794 for 5-androstene-3β,7β,17β-triol.

However, the levels of circulating metabolites of DHEA and androstenediol (Adiol), such as 7α-hydroxy-DHEA and 5-androstene-3β,7β,17β-triol (3β,7β,17β-AT), significantly decreased in FP after anti-MS treatment (Figure 3).

#### 2.1.3. Active Androgens and Estrogens

**Figure 4:**
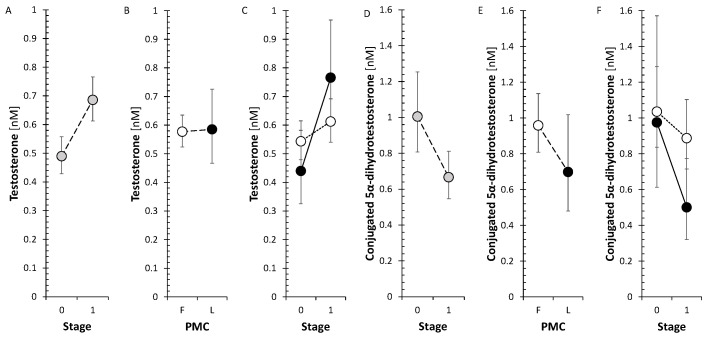
Changes of testosterone and conjugated 5α-dihydrotestosterone levels in patients with MS after treatment with anti-MS drugs. The drawings and symbols are the same as for Figure 1. The statistics for factors and interaction are as follows: Panel (**A**): Stage: F = 9.8, *p* = 0.003, η_p_^2^ = 0.154; Panel (**B**): PMC: F = 0, *p* = 0.945, η_p_^2^ = 0.0000883; Panel (**C**): Stage × PMC: F = 3.3, *p* = 0.077, η_p_^2^ = 0.0568; Subj: F = 3, *p* < 0.001, η_p_^2^ = 0.765 for testosterone and Panel (**D**): Stage: F = 4.8, *p* = 0.033, η_p_^2^ = 0.0863; Panel (**E**): PMC: F = 0.9, *p* = 0.361, η_p_^2^ = 0.0164; Panel (**F**): Stage × PMC: F = 1.5, *p* = 0.226, η_p_^2^ = 0.0286; Subj: F = 1.4, *p* = 0.1, η_p_^2^ = 0.618 for 5α-dihydrotestosterone.

From active androgens, testosterone after treatment significantly increased in LP but not in FP, conjugated 5α-dihydrotestosterone significantly decreased (Figure 4), and unconjugated 5α-dihydrotestosterone (5α-DHT) was not significantly influenced by anti-MS treatment. From estrogens, only the inactive E1 sulfate showed a significant decrease after treatment, while E2 did not change.

#### 2.1.4. Progesterone and Its Metabolites

As expected, P and its C21 Δ^4^ metabolites strongly correlated with PMC but did not exhibit a pronounced trend in changes after anti-MS treatment. Nevertheless, the levels of GABAergic 3α-hydroxy-5α/β-pregnanes showed a borderline trend to decrease in FP, while their 3-oxo-/3β-hydroxy-counterparts did not.

#### 2.1.5. C17-Hydroxylase, C17-20-Lyase (CYP17A1), and 3β-Hydroxysteroid Dehydrogenases (HSD3Bs)

**Figure 5:**
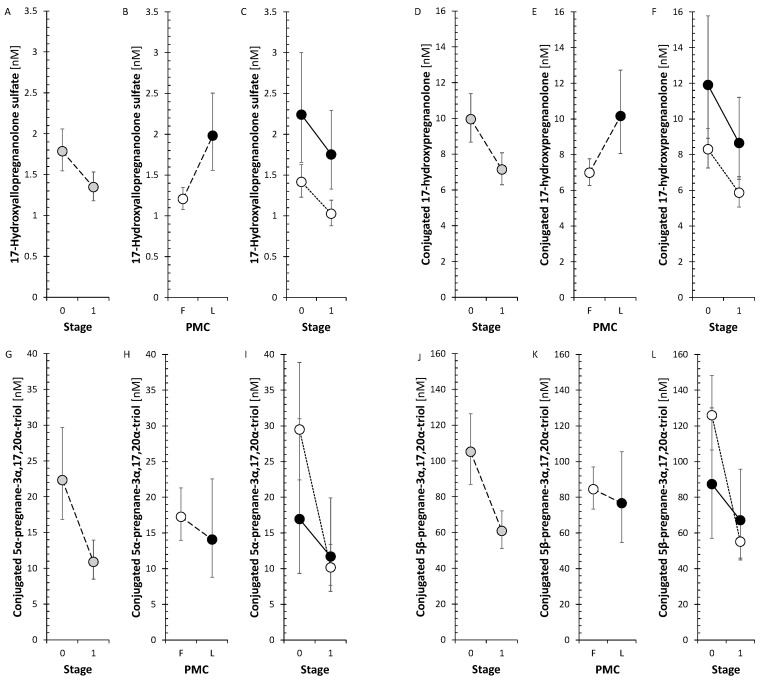
Changes of 17-hydroxyallopregnanolone sulfate, conjugated 17-hydroxypregnanolone, conjugated 5α-pregnane-3α,17,20α-triol, and conjugated 5β-pregnane-3α,17,20α-triol levels in patients with MS after treatment with anti-MS drugs. The drawings and symbols are the same as for Figure 1. The statistics for factors and interaction are as follows: Panel (**A**): Stage: F = 5.2, *p* = 0.026, η_p_^2^ = 0.087; Panel (**B**): PMC: F = 5, *p* = 0.029, η_p_^2^ = 0.0836; Panel (**C**): Stage × PMC: F = 0.1, *p* = 0.831, η_p_^2^ = 0.000837; Subj: F = 3.5, *p* < 0.001, η_p_^2^ = 0.791 for 17-hydroxyallopregnanolone sulfate; Panel (**D**): Stage: F = 8, *p* = 0.007, η_p_^2^ = 0.127; Panel (**E**): PMC: F = 3.2, *p* = 0.082, η_p_^2^ = 0.0541; Panel (**F**): Stage × PMC: F = 0, *p* = 0.961, η_p_^2^ = 0.0000441; Subj: F = 2.4, *p* < 0.001, η_p_^2^ = 0.721 for conjugated 17-hydroxypregnanolone; Panel (**G**): Stage: F = 9.1, *p* = 0.004, η_p_^2^ = 0.142; Panel (**H**): PMC: F = 0.2, *p* = 0.634, η_p_^2^ = 0.00415; Panel (**I**): Stage × PMC: F = 1.6, *p* = 0.205, η_p_^2^ = 0.029; Subj: F = 1.9, *p* = 0.011, η_p_^2^ = 0.666 for 5α-pregnane-3α,17,20α-triol; and Panel (**J**): Stage: F = 11.7, *p* = 0.001, η_p_^2^ = 0.178; Panel (**K**): PMC: F = 0.1, *p* = 0.742, η_p_^2^ = 0.00202; Panel (**L**): Stage × PMC: F = 2.6, *p* = 0.114, η_p_^2^ = 0.0455; Subj: F = 3.4, *p* < 0.001, η_p_^2^ = 0.787 for conjugated 5β-pregnane-3α,17,20α-triol.

There was no significant trend in SMRs that could reflect summary CYP17A1, including both hydroxylase and lyase steps, activity and CYP17A1 activity separately in the hydroxylase and lyase steps. Otherwise, all of the conjugated 17-hydroxy-5α/β-pregnanes decreased significantly in FP (Figure 5), but none of their unconjugated counterparts, whereas of the 17-hydroxy-Δ^5^ and Δ^4^ steroids, only cortisol, measured by both RIA and GC-MS/MS, decreased significantly in FP.

The SMRs, which may reflect HSD3B activity, did not show a significant trend toward changes after treatment with anti-MS drugs.

#### 2.1.6. Balance Between Steroid Sulfotransferase (SULT2A1) and Steroid Sulfatase (STS)

The ratios of conjugated steroids to their unconjugated counterparts that may reflect a balance between steroid sulfotransferase SULT2A1 and steroid sulfatase STS tended to decrease in FP. The same was valid for the levels of conjugated steroids.

#### 2.1.7. 11β-Hydroxylase (CYP11B1) and Type 1 11β-Hydroxysteroid Dehydrogenase (HSD11B1)

SMRs that may reflect CYP11B1 activity did not reach significance (probably due to insufficient data) but showed a borderline trend to decrease after treatment with anti-MS drugs in LP. A low number of SMRs, which may reflect HSD11B1 activity, were also observed to assess the trend of changes after treatment. However, these data did not even suggest such a trend.

#### 2.1.8. 7α-, 7β-, and 16α-Hydroxylating Enzymes (CYP7B1, CYP3A4, and CYP3A7)

The SMRs that may reflect the activities of pluripotent 7α/β- and 16α-hydroxylating enzymes showed a borderline trend to decrease after treatment in FP. Due to the low number of indices (*n* = 8), this trend did not reach significance, but still, 4 of them decreased after treatment with anti-MS drugs, and 4 showed no significant change.

#### 2.1.9. 5α-Reductases (SRD5As), 5β-Reductase (AKR1D1), and Aldoketoreductases Subfamily1c (AKR1C1-4))

The SMRs that may reflect the activities of SRD5As, AKR1C1, AKR1C2, and AKR1C3 did not show consistent trends in changes after treatment.

### 2.2. Effect of Anti-MS Drugs on Circulating Steroids and Steroid Molar Ratios (SMRs)

In addition to the aggregate evaluation of the effects of treatment on the steroidome, the effects of individual anti-MS drugs were evaluated as well. The steroidomic changes after treatment with differentiation between individual anti-MS drugs are shown in Table S2. The results of the Wilcoxon one-sample test with correction for continuity, which was used to assess the prevailing trend of changes in steroid levels or SMRs in MS patients after treatment with individual anti-MS drugs, are summarized in Table 2, separately for FP and LP.

The following drugs were used: GA (Copaxone) FP *n* = 6, LP *n* = 2; dimethyl fumarate (Tecfidera) FP *n* = 2, LP *n* = 1; natalizumab (Tysabri) FP *n* = 1, LP *n* = 0; interferons β1a (Avonex, Rebif, Plegridy FP *n* = 6, LP *n* = 5); inhibitors of the sphingosine-1-phosphate receptor (S1PR) (Ponvory, Zeposia) FP *n* = 5, LP *n* = 1; teriflunomide (Aubagio) FP *n* = 3, LP *n* = 0; ofatumumab (Kesimpta) FP *n* = 7, LP *n* = 1; ocrelizumab (Ocrevus) FP *n* = 11, LP *n* = 5; MS patients in Stage 1 without medication FP *n* = 2, LP *n* = 2. Due to the low numbers of applications of these drugs, the effects of some of them were monitored in both PMCs, while others were monitored only in the follicular phase of the menstrual cycle (FP), and some drugs were not included in the evaluation at all.

#### 2.2.1. Glatiramer Acetate (GA) in the Follicular Menstrual Phase

GA treatment did not show a significant trend in changes for steroid levels, levels of corticoids and 11β-hydroxy-androgens, Δ^5^ and Δ^4^ steroids, P 5α/β-reduced metabolites, summary CYP17A1 activity, activities in CYP17A1 hydroxylase step, CYP17A1 lyase step, HSD3Bs, conjugated/unconjugated steroids ratio, CYP11B1, CYP7B1, HSD11B1, SRD5As, and in balances AKR1C1 vs. HSD17B2, AKR1C2 vs. HSD17B2 and HSD17B6, and AKR1C3 vs. HSD17B2.

GA therapy induced a trend of decreased ratios of conjugated steroids to their unconjugated counterparts. Also, the SMRs that may reflect the balance between reductive AKR1C1 on one hand and oxidative HSD17B2 enzymes showed a trend toward a shift to the latter one. Although the trend in SMRs that may reflect the activity of CYP17A1 in the lyase step was insignificant; specifically, the DHEA/17-OH-Preg and A/17-OH-P ratios were higher after GA treatment. Concerning the effects of GA treatment on the levels of androstenedione and active androgens (T, 5α-DHT, and 5α-DHTC), their number was low to assess their pooled trend after anti-MS treatment. Nevertheless, GA did not show any effect on the levels of the aforementioned steroids.

#### 2.2.2. Interferons β1a (IFN-β1a)

In FP, the treatment with IFN-β1a did not show a significant trend in changes after treatment for steroid levels and specifically for corticoids and 11β-hydroxy-androgens, HSD3Bs, conjugated/unconjugated steroids ratios, CYP11B1, CYP7B1, HSD11B1, SRD5As, and AKR1D1. The balance AKR1C3 vs. HSD17B2 showed indications of significant trends to decrease in the levels of Δ^5^ and Δ^4^ steroids and of P 5α/β-reduced metabolites. In FP, the treatment with IFN-β1a displayed a significant trend to higher values for CYP17A1 in the lyase step but an insignificant indication of a trend to lower values for CYP17A1 in the hydroxylase step. From the biologically most important steroids, also the levels of T, 5α-DHT, and cortisol measured by GC-MS/MS (but not by RIA) showed significant increase after IFN-β1.

In the LP, the treatment with IFN-β1a did not show significant trends in changes after treatment for summary CYP17A1 activity, CYP17A1 activity in the hydroxylase step, CYP17A1 activity in the lyase step, HSD3Bs, conjugated/unconjugated steroids ratio, CYP11B1, CYP7B1, HSD11B1, SRD5As, AKR1D1, and balances AKR1C2 vs. HSD17B2, HSD17B6, AKR1C2 vs. HSD17B2 and HSD17B6, and AKR1C3 vs. HSD17B2. However, there was a pronounced pooled trend to higher steroid levels, specifically higher levels of corticoids and 11β-hydroxy-androgens, Δ^5^ and Δ^4^ steroids, and P 5α/β-reduced metabolites and a significant trend to higher SMRs that may reflect AKR1D1 activity.

#### 2.2.3. Inhibitors of the Sphingosine-1-Phosphate Receptor (S1PRI) in the Follicular Menstrual Phase

S1PRI treatment did not significantly change the levels of corticoids and 11β-hydroxy-androgens, Δ^5^ and Δ^4^ steroids, CYP17A1 activity in the lyase step, HSD3Bs, conjugated/unconjugated steroid ratios, CYP11B1, CYP7B1, HSD11B1, AKR1D1, and balances AKR1C1 vs. HSD17B2 and AKR1C2 vs. HSD17B2 and HSD17B6.

S1PRI treatment showed an indication of insignificant trend to lower levels of Δ^5^ and Δ^4^ steroids, lower summary CYP17A1 activity in the hydroxylase step, and a borderline trend to lower summary CYP17A1 activity. On the one hand, S1PRI treatment resulted in a significant pooled trend to decrease steroid levels and specifically for the levels of P 5α/β-reduced metabolites. On the other, there was a significant trend to increase SMSs that may reflect SRD5As activity. S1PRI did not show any effect on the levels of androstenedione and active androgens (T, 5α-DHT, and 5α-DHTC).

#### 2.2.4. Ofatumumab (Kesimpta) in the Follicular Menstrual Phase

Ofatumumab treatment did not show a significant trend in changes in the levels of corticoids and 11β-hydroxy-androgens, summary activity of CYP17A1, activities of CYP17A1 in the hydroxylase and lyase steps, conjugated/unconjugated steroid ratios, CYP11B1, HSD11B1, SRD5As, AKR1D1, and balances between activities of AKR1C2 vs. HSD17B2 and HSD17B6.

Ofatumumab treatment resulted in a borderline trend to decrease for HSD3Bs and significant trend to decrease circulating steroids, specifically Δ^5^ and Δ^4^ steroids, P 5α/β-reduced metabolites, and SMRs that may reflect activities of CYP7B1, CYP3A4, and CYP3A7 enzymes and a significant trend to decrease for the balance between reductive AKR1C3 and oxidative HSD17B2 activities.

#### 2.2.5. Ocrelizumab (Ocrevus)

In the FP, ocrelizumab treatment did not show a significant trend regarding the levels of corticoids and 11β-hydroxy-androgens; SMRs that may reflect summary CYP17A1 activity; activities of HSD3Bs, CYP11B1, HSD11B1, SRD5As, and AKR1D1; and SMRs that may reflect balances between activities of reductive AKR1C1 vs. oxidative HSD17B2, reductive AKR1C2 vs. oxidative HSD17B2 and HSD17B6 (also possessing 3α/β-hydroxy-isomerase activity), and also reductive AKR1C3 vs. oxidative HSD17B2. Despite the absence of a significant trend in corticoid and 11β-hydroxy-androgen levels, the most important of these, cortisol, increased significantly in FP after ocrelizumab treatment.

In FP, ocrelizumab treatment also resulted in a borderline trend to decrease for SMRs that may reflect CYP7B1 activity and significant trend to decrease for steroid levels, specifically for the levels of P 5α/β-reduced metabolites and a significant trend to decrease for SMRs that may reflect activities of CYP17A1 in the hydroxylase step and, at the same time, a significant trend to increase for SMRs that may reflect activities of CYP17A1 in the lyase step and a significant trend to decrease for conjugated/unconjugated steroid ratios.

In the LP, ocrelizumab treatment did not show a significant trend in the levels of P 5α/β-reduced metabolites, SMRs that may reflect activities of CYP17A1 in the lyase step, HSD3Bs, CYP11B1, HSD11B1, and SMRs that may reflect balances between activities of reductive AKR1C1 vs. oxidative HSD17B2, reductive AKR1C2 vs. oxidative HSD17B2 and oxidative HSD17B6 (also possessing 3α/β-hydroxy-isomerase activity).

In the LP, ocrelizumab treatment showed an indication of insignificant trend to lower SMRs that may reflect a balance between activities of reductive AKR1C3 vs. oxidative HSD17B2. In the LP, ocrelizumab treatment also showed a borderline trend to decrease for the levels of Δ^5^ and Δ^4^ steroids and for the SMRs that may reflect activities of CYP7B1, CYP3A4, and CYP3A7 enzymes; a significant trend to decrease for the levels of steroids and specifically for corticoids and 11β-hydroxy-androgens; a significant trend to decrease for SMRs that may reflect summary CYP17A1 activity and activities of CYP17A1 in the hydroxylase step; conjugated/unconjugated steroid ratios; and SMRs that may reflect activity of AKR1D1. It should also be noted that from individual circulating steroids in the LP, P, 5α-DHP, and 3β,5α-THP positively correlated with ocrelizumab treatment.

## 3. Discussion

### 3.1. Changes in Steroid Levels and Steroid Molar Ratios (SMRs) in MS Patients After Treatment with Anti-MS Drugs

While our previous results evaluated steroidomic changes in drug-naive female MS patients in FP compared to age-matched female controls in FP [33], our current data showed steroidomic changes in initially drug-naive female MS patients after treatment with anti-MS drugs.

While the aggregated results from our previous study showed a significant trend toward lower steroid levels in patients in FP [33], this study demonstrated a significant trend to further decrease steroid levels in MS patients after anti-MS treatment in FP.

Female sex hormones in FP are synthesized either directly in the adrenal glands or in peripheral tissues from adrenal precursors, while steroids in mid-cycle and LP are predominantly of ovarian origin [34]. The results from our previous study suggested an overall trend toward impaired adrenal activity in patients, which may affect the synthesis of most steroids (including active steroid hormones and neuroactive, neuroprotective, and immunoprotective steroids) found downstream in the metabolic pathway [33]. However, our previous data demonstrated a trend toward positive correlations of steroid levels with the MS severity. This was interpreted that although lower steroid levels could be related to the onset of the disease, there could be a counter-regulation toward an increase during the progression of the disease.

**Figure 6:**
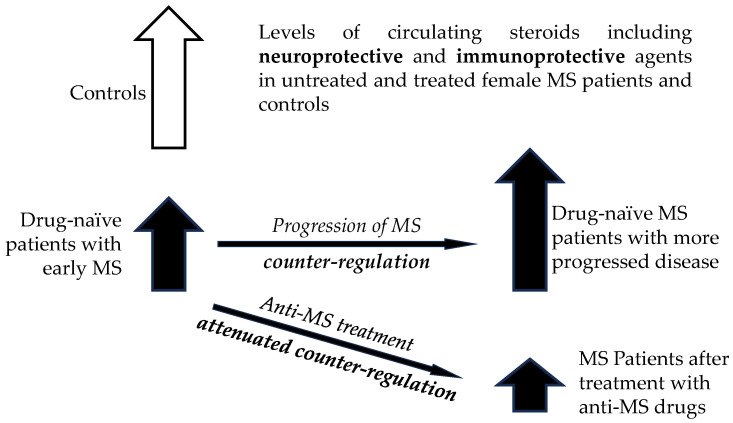
Scheme illustrating alterations in circulating steroids in patients with early MS, advanced MS and controls, and in patients treated with anti-MS drugs.

In addition, lower steroid levels were suggested as an indicator of a predisposition to MS [33]. Nevertheless, during the development of MS, the differences between steroid levels in patients and controls diminished, which could hinder the differentiation of patients with more serious MS from controls [33]. Our current data show that anti-MS treatment tends to further decrease the levels of predominantly adrenal steroids in FP, but the general trend is absent in LP. In the latter, the steroidogenesis is more dependent on the ovarian activity (see Figure 6).

In terms of changes in steroid levels after treatment with individual anti-MS drugs, significant general trends to decrease were found for S1PRI, ofatumumab in FP, and also ocrelizumab in both PMCs but a trend to increase for IFNβ-1a in LP.

The steroidogenesis is mainly under adrenal control in the FP. Alternatively, in the LP, the steroidogenesis is predominantly controlled by the ovarian activity. The attenuation of steroidogenesis in FP after therapy with anti-MS drugs may be explained as a weakening of protective steroid-dependent counterregulatory mechanisms depending on adrenal activity due to the beneficial effect of anti-MS therapy. In addition, in play could be the anti-steroidogenic effect of key anti-MS drugs on adrenal steroidogenesis. The trend toward increased steroid levels after IFN-β1a in LP included important bioactive steroids as the neuroprotective pregnenolone and the steroid hormones as testosterone, 5α-DHT, and cortisol, which indicated a beneficial effect of IFN-β1a treatment in LP in terms of MS severity.

Regarding the effect of GA on steroidogenesis, Cil et al. did not find changes in FSH and E2 levels in MS patients treated by combined therapy with interferon β and GA; however, the authors reported diminished ovarian volume and follicular reserve in MS patients compared to age-matched healthy controls [20]. Our current results showing no effect of GA on circulating steroids in FP are in line with the data from Cil et al. [20].

Regarding the effect of IFN-β1a treatment on steroidogenesis, the only reference in the literature was from Griffiths and Wang [21], who stated that cholesterol 25-hydroxylase (CH25H) is an interferon-stimulated gene and 25-hydroxycholesterol can be considered as an immunoregulatory oxysterol that has a role in the adaptive immune system.

As to the steroidomic effect of S1PRI, Lucki and Sewer reported that S1P rapidly increased cortisol biosynthesis and the expression of genes participating in cholesterol uptake and transport in H295R adrenocortical cells [23]. Also, LH and FSH promoted S1P synthesis in the follicular cells via activation of protein kinase C. Moreover, S1P is an important mediator of FSH and LH proliferation, survival, and viability effects in follicular cells [24]. However, a borderline trend to decrease in circulating corticoids and 11β-hydroxy-androstanes in association with S1PRI treatment was observed in our current data similarly, like for the general trend in circulating steroids.

**Figure 7:**
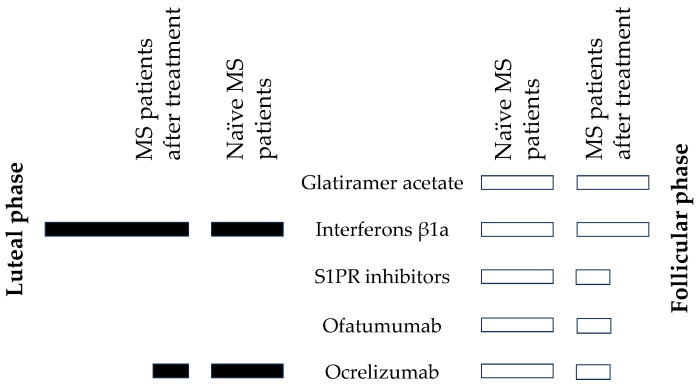
Scheme illustrating changes in circulating steroids in patients treated with individual anti-MS drugs.

Although there is still no information on the effect ofatumumab on the steroidome, another anti-MS drug, which is also based on the anti-CD20 monoclonal antibody ocrelizumab, suppressed the production of a number of androstane and pregnane steroids [25], which is consistent with the attenuation of steroidogenesis after treatment with the above drugs observed in this study. The effects of individual anti-MS drugs are illustrated in Figure 7.

#### 3.1.1. Corticoids (C21 Δ^4^ Steroids) and 11β-Hydroxy-Androgens (C19 Δ^4^ and 5α/β Steroids), 11β-Hydroxylase (CYP11B1), and Type 1 11β-Hydroxysteroid Dehydrogenase (HSD11B1)

MS is an autoimmune disease induced by autoreactive T lymphocytes that is characterized by an imbalance of pro-inflammatory cytokines, such as TNF, interferon γ (IFN-γ), IL-2 and lymphotoxin, and regulatory cytokines (e.g., IL-4 and IL-10). These substances stimulate pituitary gland via the hypothalamus to produce adrenocorticotropic hormone (ACTH), which in turn stimulates cortisol production in the *zona fasciculata* [35,36,37]. The secretion of ACTH is controlled by hypothalamic corticotropin-releasing hormone (CRH), pituitary ACTH, and cytokines. The bioavailability of cortisol in target tissues depends on its interconversion to cortisone, which is inactive. The balance between bioactive cortisol and inactive cortisone is regulated by the reductive enzyme HSD11B1 and the oxidative enzyme HSD11B2. In contrast to HSD11B1, which may influence cortisol circulating levels, the latter enzyme is primarily expressed in selective tissues acting mainly in paracrine and autocrine manner and does not substantially influence the circulating cortisol [38]. Glucocorticoids play a decisive role in the regulation of the immune system and act through binding to the low-affinity glucocorticoid receptors. Although glucocorticoids are mainly a product of the adrenal *zona fasciculata*, they can be produced extra-adrenally, for example, in cells of the immune system, intestine, skin, or brain [38]. Our current data did not find a significant trend in SMRs that may reflect the HSD11B1 activity (*n* = 4), but this result cannot be considered as conclusive due to the small number of relevant SMRs and, thus, the low statistical power of the test.

In addition to the response to emotional and cognitive stress, HPAA also regulates the interaction between peripheral inflammatory processes and cortisol secretion [39,40]. Most authors have reported that MS patients have overactive HPAA with elevated cortisol levels (summarized in [41]), but others found unchanged circulating cortisol levels in patients or even reduced cortisol levels in the cerebrospinal fluid of patients compared with controls [42,43]. Foroughipour et al. [43] indicated that chronic activation of HPAA occurs in patients. However, Wei and Lightman suggested that HPAA is unlikely to play a major role in the initial pathogenesis of MS, but the overactivation of the HPAA in MS patients is secondary to an active inflammatory stimulus [44].

Our current data showed that the anti-MS treatment reduced the levels of active glucocorticoid cortisol and corticosterone, which is a precursor of the mineralocorticoid aldosterone pathway, as well as the inactive metabolite of cortisol, cortisone. As the SMRs that may reflect CYP11B1 did not show significant change, this trend may be ascribed to the attenuated adrenal activity after anti-MS treatment instead of blocked CYP11B1 activity. However, the number of above SMRs was low (*n* = 7); therefore, a definite conclusion could not be made.

Reduced cortisol secretion in response to chronic inflammation has been reported in patients with autoimmune diseases compared to healthy subjects (see review [5]). Some authors consider that the relative adrenal insufficiency in these patients may be secondary to impaired hepatic metabolism of steroid hormones. The key enzyme involved in the conversion of inactive cortisone to active cortisol, 11β-hydroxysteroid dehydrogenase type 1 (HSD11B1), is significantly stimulated by TNF and other pro-inflammatory cytokines, and the conversion of inactive cortisone to active hormone takes place primarily in the liver. According to this hypothesis, the stimulation of HSD11B1 and the subsequent increased concentration of the active glucocorticoid cortisol could induce a negative feedback loop and, thus, dysfunction of the HPAA [11] (see review [5]).

Our previous study indicated an absence of serious blocks in the pathway of cortisol synthesis, which was indicated by lower levels of 17-OH-P at elevated levels of cortisol and cortisone [33]. Our current data also suggested an absence of specific enzyme blocks in the cortisol pathway associated with anti-MS treatment (unaffected MS treatment CYP17A1, HSD3B, CYP11B1, and HSD11B1) activities. The only change associated with anti-MS treatment on the cortisol pathway was an increase in pregnenolone levels, but this occurred with a parallel increase in DHEA levels, again indicating an absence of enzyme blocks.

Corticoid and 11β-hydroxy-androgen levels showed no significant trend after GA treatment in FP. However, IFNβ-1a treatment in LP increased the levels of these steroids, whereas ocrelizumab suppressed them in this menstrual phase. These changes in LP were consistent with trends found for steroids in general and like the pooled trends of steroids in LP for IFN-β1a and ocrelizumab treatments. From the biologically most important steroids, also the levels of cortisol showed significant increase after IFN-β1 and ocrelizumab treatment in LP.

No changes were observed in corticosteroid levels after S1PRI treatment in our present data. Our data are not consistent with the results of Lucki and Sewer, who reported that S1P rapidly increased cortisol biosynthesis [23].

Of note, our data for cortisol (Figure 1) were in line with data from Hamidović et al. [45], who proved using meta-analysis that the cortisol levels were lower in the follicular phase.

#### 3.1.2. Δ^5^ and Δ^4^ Steroids

Our previous study did not find significant associations of MS with these steroids except for lower levels of 7β-hydroxy-DHEA (7β-OH-DHEA). Concerning the changes of Δ^5^ and Δ^4^ steroids in association with anti-MS treatment, there was a parallel increase in pregnenolone and DHEA levels, as mentioned above, which may be of importance as both steroids are neuroprotective. They cross the BBB; therefore, their adrenal production and/or therapeutic supplementation may affect their concentration in the brain [13,14]. Pregnenolone is pro-cognitive and neuroprotective against glutamate-induced neurotoxicity [15]. Furthermore, sulfates of pregnenolone and DHEA positively modulate NMDARs and σ1 receptors (σ1R) [6,15,46]. The protective effects of DHEA and its sulfate can be attributed to their modulation of type A GABA receptors (GABA_A_R) and protection of mitochondria from intracellular Ca^2+^ overload [15]. In addition, DHEA and its sulfate have antioxidant and anti-inflammatory potential also outside the CNS [16,47]. Therefore, specific increases in pregnenolone and DHEA can be considered one of the beneficial effects of anti-MS therapy. From the anti-MS drugs, GA did not show an effect on Δ^5^ and Δ^4^ steroids. In the FP, the treatment with IFNβ-1 and with S1PRI showed an insignificant indication of a decrease of these steroids, treatment with ocrelizumab showed a borderline trend to their decrease, and the treatment with ofatumumab exhibits a trend to decrease the levels of the Δ^5^ and Δ^4^ steroids. In the LP, ocrelizumab treatment had the same effect as in the FP. These trends were also similar for all steroids.

#### 3.1.3. Active Androgens and Estrogens

Androstenedione metabolite T, like T metabolite 5α-DHT, are important neuromodulators. However, their neuromodulatory effects are sex-dependent (see reviews [48,49]). T is a modulator of the mesocorticolimbic system affecting the density of dopaminergic neurons [47]. Nevertheless, only T, not 5α-DHT, has a direct neuroprotective effect, suggesting that T and 5α-DHT may have independent effects on the hippocampal immune cells (see review [49]).

The experiments with EAE, induced by helper T lymphocytes, showed that MS patients have a predilection for Th1, which may be associated with low T levels (see review [49]). The latter finding is in accordance with our previous study [33].

Surprisingly, RRMS patients with higher T levels were more likely to have tissue damage [50]. Based on correlations with MRI data, the authors [50] considered the increase of protective steroids E and T to be a response to brain damage [50]. The active androgens shift from Th1 to Th2 phenotype. Increased IFN-γ secretion due to this change may contribute to the known susceptibility of female experimental animals to induction of autoimmune diseases, including EAE [1].

Other effects of T include proliferation and differentiation of lymphocytes and inhibition of immunoglobulin production. Furthermore, T can penetrate the BBB and protect neuronal cells from glutamate toxicity and increase neurite outgrowth and brain-derived neurotrophic factor expression [1]. Androgens also indirectly increase T cell thymocyte apoptosis and thus exert neuroprotective effects in EAE (see review [21]). In addition, T reduces reactive gliosis and astrocyte proliferation, which promotes axon regeneration and brain lesion repair in MS patients and animal models [51,52,53,54]. However, sex hormones can not only protect brain tissue, but also they can have excitotoxic and apoptotic effects [55] (see also review [48]).

Data from our previous study showed lower levels of A and T compared to controls but non-significantly different levels of conjugated and unconjugated 5α-DHT [33].

Data from the current study show an increase in circulating T after anti-MS treatment for LP with unchanged A levels but show a decrease in conjugated 5α-DHT levels. The observed increase in T levels after treatment could be related to the progression of the disease but also to the effect of anti-MS treatment itself. The increase in T levels with unchanged A levels is consistent with the results for the T/A ratio, which could reflect a shift in the balance between the activities of the reductive AKR1C3 and oxidative HSD17B2 to the former enzyme. However, in the aggregate, no significant trend was found for this balance in terms of treatment effect. Since the literature data point to predominantly positive effects of T on the condition of MS patients, it can be assumed that anti-MS treatment also has a positive effect in this respect, at least in LP.

With regard to the active estrogen E2, unlike testosterone, no changes in circulating levels were detected after anti-MS treatment, regardless of the PMC. As for the decrease in E1 sulfate levels, this result is consistent with the pooled trend toward a decrease in circulating steroid levels after anti-MS treatment.

Concerning the effects of anti-MS treatment on androstenedione and active androgens (T, 5α-DHT, and 5α-DHTC), the number of variables was insufficient to assess their pooled trend after anti-MS treatment. Nevertheless, while GA, IFNβ-1, and S1PRI in FP did not show any effect, T and 5α-DHT were higher after IFNβ-1a therapy in LP. Androstenedione and 5α-DHT decreased after ofatumumab therapy, and 5α-DHT decreased after ocrelizumab treatment in LP but not in FP.

#### 3.1.4. Progesterone and Its 5αβ-Reduced Metabolites

The P metabolite 3α,5α-THP has various neuroprotective effects, including alleviation of neurobehavioral deficits and attenuation of neuropathology and inflammation in animal models of autoimmune demyelination [18,19]. Moreover, recent studies demonstrated that 3α,5α-THP suppresses neuroinflammation through activation of TLR4 protein in macrophages and in the brain [51].

Although there were no consistent changes in circulating P 5α/β-reduced metabolites, levels of 3α-hydroxy-5α/β-steroids tended to decrease in FP after anti-MS treatment, whereas their 3β-hydroxy counterparts did not. Thus, the decline in the previously mentioned steroids that are neuroprotective could also be related to the attenuated counterregulatory activity against the effects of MS due to the beneficial effects of anti-MS treatment.

From the anti-MS drugs, GA did not show an effect on P 5α/β-reduced metabolites. IFNβ-1a treatment in the FP indicated an insignificant tendency to decrease these steroids, whereas the treatment with S1PRI, ofatumumab, and ocrelizumab reached significance. In the LP, IFNβ-1a treatment showed a significant trend to increase, while for ocrelizumab, there was no significant trend. Progesterone, 5α-DHP, and 3β positively correlated with ocrelizumab treatment in LP. Progesterone has promyelinating effects on the CNS, acts against glutamate toxicity, normalizes functional defects of injured neurons, and enhances proliferation and differentiation of oligodendrocyte precursor cells. In addition, P is an immunomodulator that shifts the pro-inflammatory Th1 response to an anti-inflammatory Th2 response and promotes a reduction in IFN-γ production by natural killer cells [56,57] (see also review [48]). Therefore, the increase in P levels in LP after treatment with ocrelizumab could have a beneficial effect on the severity of MS.

#### 3.1.5. C17-Hydroxylase, C17-20-Lyase (CYP17A1), Hydroxylase, and 3β-Hydroxysteroid Dehydrogenases (HSD3Bs)

The SMRs, which may reflect summary CYP17A1 activity in the hydroxylase and lyase steps, did not show a significant trend for change after anti-MS treatment. However, the SMRs, which may reflect CYP17A1 activity separately in the hydroxylase step, showed a borderline trend toward a decrease in FP but a borderline trend toward an increase in CYP17A1 activity in the lyase step. In addition, all conjugated 17-hydroxy-5α/β-pregnanes showed a significant decrease in the FP (Figure 5).

In this context, Gupta et al. reported that 17-hydroxyallopregnanolone (3α,5α17-PD) was rapidly converted to androsterone (3α,5α-THA) under CYP17A1 catalysis in a lyase step, which occurred even in the absence of cytochrome B5 (CYB5) [58]. The authors found that 3α,5α17-PD was a better substrate for CYP17A1 than 17-OH-Preg. It should be mentioned that CYB5, which activates the CYP17A1 lyase step, is expressed about four times more in the adrenal cortex than in other tissues (http://biogps.org/#goto=genereport&id=80777, accessed on 26 December 2024). However, CYP17A1 is more than 1500 times expressed in the adrenal cortex compared to the other tissues and approximately 70 and 40 times more in the kidneys and testes, respectively (http://biogps.org/#goto=genereport&id=1586, accessed on 26 December 2024).

These data suggest that the adrenal cortex forms mainly 17-hydroxy C21 steroids and C19 Δ^5^ steroids from their 17-deoxy C21 precursors, whereas 5α/β-reduced steroids are formed mainly extra-adrenally, independent of the enzyme CYB5, which is weakly expressed outside the *zona reticularis* of the adrenal gland [59]. This may be the reason why in the synthesis of Δ^5^ androgens, the adrenal cortex prefers the Δ^5^ pathway, while in other tissues, the synthesis of 5α/β-reduced androstanes occurs, and it is independent of CYB5. Our results showed very low circulating levels of intermediates, such as 17-hydroxyallopregnanolone sulfate (3α,5α,17-PDC), 3α,5β,17-PD, and 3α,5β,17-PD) in the pathway from 17-deoxy-5α/β-reduced pregnanes to 5α/β-reduced androstanes, which indicated their rapid conversion to the corresponding androstanes.

There was no significant trend in SMRs that could reflect summary CYP17A1 activity and CYP17A1 activities separately in the hydroxylase and lyase steps. Otherwise, all of the conjugated 17-hydroxy-5α/β-pregnanes decreased significantly in FP (Figure 5) but none of their unconjugated counterparts, whereas of the 17-hydroxy-Δ^5^ and Δ^4^ steroids, only cortisol decreased significantly in FP. In the present study, the trend toward a decrease in 17-hydroxy-pregnanes after treatment with anti-MS drugs in FP, but not the one in SMRs, suggests a possible downward shift in CYP17A1 activity in the hydroxylase step. This may prompt a weakening of one of the steps in the pathway toward cortisol. Our data also indicate that monoclonal anti-CD20 mAbs, S1PRI, and IFNβ-1a may be responsible for attenuating CYP17A1 activity while enhancing the lyase step. In LP, only ocrelizumab, but not IFNβ-1a, promoted total CYP17A1 activity and CYP17A1 activity at the hydroxylase step, with no effect at the lyase step. To summarize, the aforementioned findings could have implications for the synthesis and metabolism of bioactive steroids, including cortisol, active androgens and estrogens, or neuroactive and neuroprotective substances.

In the case of HSD3Bs (involved in the synthesis of cortisol as well as active androgens and estrogens), the anti-MS drug treatment does not seem to affect their activity.

#### 3.1.6. Balance Between Steroid Sulfotransferase (SULT2A1) and Steroid Sulfatase (STS)

Regarding neuroactive steroids, unconjugated steroids and their corresponding sulfates are often antagonists at the same receptors or may act antagonistically in relation to neuronal activity at different receptors [60,61,62]. Preg and DHEA sulfates are known to modulate several types of ionotropic receptors, such as NMDARs, AMPARs, nicotinic receptors, TRPM3s, TRPC5s, or TRPV1s, and may improve cognitive function while counteracting pain and fear transmission [7,17].

The aggregated data in our previous study showed a significant trend toward lower levels of unconjugated steroids but not in steroid conjugates in MS patients compared to controls. This finding was ascribed to increased SULT2A1 activity in MS patients compared to controls. Moreover, the SMRs that may reflect the balance between SULT2A1 and STS showed a significant trend toward elevated values in patients compared to controls, which indicated increased SULT2A1 activity in MS patients.

Our current data showed a significant trend to decrease the ratios of conjugated steroids to their unconjugated counterparts in FP after anti-MS therapy, which indicated either suppression of SULT2A1 activity and/or stimulation of STS activity by anti-MS drugs. In addition to a significant pooled trend toward a decrease in the ratios of conjugated steroids to their unconjugated counterparts after treatment with anti-MS drugs, a significant trend toward a decrease in these ratios was found for GA in FP, while for ocrelizumab, this trend was observed in both PMCs. Moreover, our current data showed a significant trend toward a decrease in circulating conjugated steroids in FP.

These data may be of relevance for regulation of neuronal excitability. Unconjugated steroids act through binding to nuclear receptors while their sulfates are inactive. Nevertheless, steroids may be accumulated in the sulfate form and may be consequently hydrolyzed to active hormones [63]. Furthermore, sulfation of unconjugated neuroinhibitory and neuroprotective GABAergic steroids leads to their inactivation or even to the formation of their antagonists [61,64]. Conversely, some sulfated steroids can be positive or negative modulators of excitatory glutamate receptors, whereas all their unconjugated analogues are inactive in this context [65,66]. In addition, sulfated androgens may serve as a reservoir of substrates for the synthesis of bioactive androgens acting as sex hormones, neuroprotective and immunomodulatory agents.

#### 3.1.7. 11β-Hydroxylase (CYP11B1) and 11β-Hydroxysteroid Dehydrogenase Type 1 (HSD11B1)

11β-Hydroxylase (CYP11B1) and 11β-hydroxysteroid dehydrogenase type 1 (HSD11B1) are important enzymes participating in cortisol synthesis and homeostasis. CYP11B1 catalyzes the final step in cortisol synthesis converting 11-deoxycortisol to cortisol as well as 11-deoxy-androstanes to 11β-hydroxy-androstanes. 11β-Hydroxy-androstanes cannot be formed from cortisol and its 5α/β-reduced metabolites under CYP17A1 catalysis in the lyase step [67]. Our present data show a borderline trend toward a decrease in CYP11B1 after anti-MS therapy, which could lead to a decrease in cortisol synthesis in this last metabolic step. However, the effect of individual anti-MS drugs on SMRs that may reflect CYP11B1 activity was not observed, probably due to the low number of relevant SMRs combined with an insufficient number of patients.

HSD11B1 is an important diabetogenic enzyme that converts inactive cortisone to the active glucocorticoid cortisol [68]. However, our current data do not even suggest that anti-MS treatment could affect the activity of HSD11B1, which otherwise increases the level of the circulating glucocorticoid cortisol at the expense of inactive cortisone.

#### 3.1.8. 7α-, 7β-, and 16α-Hydroxylating Enzymes (CYP7B1, CYP3A4, and CYP3A7) and 11β-Hydroxysteroid Dehydrogenase, Type 1 (HSD11B1)

In our previous study [33], 7α-hydroxy-DHEA and 3β,7β,17β-AT showed reduced levels in MS patients vs. controls despite DHEA levels being significantly higher in patients. Concerning our current data, although the levels of 7α/β- and 16α-hydroxy-steroids did not show a significant trend toward changes after treatment with anti-MS drugs, the SMRs of 7α/β- and 16α-hydroxy-steroids to the parent steroids suggested a reduction in the activities of the enzymes catalyzing their synthesis (CYP7B1, CYP3A4, and CYP3A7) of borderline significance despite the low number of corresponding SMRs (*n* = 8). Regarding individual anti-MS drugs, there was a trend toward lower values of the above SMRs reaching significance with ofatumumab, a non-significant indication of this trend with ocrelizumab in FP and the same trend of borderline significance with ocrelizumab in LP, suggesting which anti-MS drugs contribute to the pooled effect of anti-MS therapy.

It should be pointed out that reduced activity of 7α/β- and 16α-hydroxylation enzymes may have a significant impact not only on the etiopathogenesis of the disease, but also on its course and further development. It is clear from the abundant data in the literature that Δ^5^ androstanes (and particularly their immunoprotective and antidiabetic 7α/β- and 16α-hydroxy-metabolites) alleviate the severity of autoimmune diseases, while these diseases can suppress the synthesis of adrenal Δ^5^ androstanes [69,70,71,72,73,74,75]. Thus, the latter finding could be related to the reduced activities of 7α/β- and 16α-hydroxylation enzymes in MS patients compared to controls. Interestingly, there is a further reduction of these enzymes in MS patients as a result of treatment with anti-MS drugs.

DHEA is known to control the Th1/Th2 balance and either favors the Th1 component or attenuates the production of both components [73,76]. The Δ^5^ androstanes also suppress cellular immunity and autoantibody production [71,72,73,74,77] and are capable of forming a Th1-dominated cytokine profile. The Δ^5^ androstanes and their 7α/β-,16α-hydroxylated metabolites may also prevent suppression of the primary immune response by glucocorticoids [78].

Two mechanisms have been proposed to explain the immunomodulatory effects of 7α/β- and 16α-hydroxy-metabolites of Δ^5^ androstanes. The first mechanism has suggested a link between the immunomodulatory effects of 7α/β-hydroxy-∆^5^-androstanes and their competition for active sites on 11β-hydroxysteroid dehydrogenase (HSD11B1), an enzyme that catalyzes the conversion of inactive cortisone to immunosuppressive cortisol [79,80].

The second mechanism has suggested that E2 may induce the autoimmune response via estrogen receptors and catabolism of estrogen precursors, such as DHEA and Adiol, to their 7α/β-, and 16α-hydroxylated metabolites reduce the concentration of these estrogen precursors and, consequently, E2 [81]. In addition, E2 promotes the catalytic activity of CYP7B1 and may thus have a feedback effect on the regulation of DHEA, Adiol, and its own levels [82].

Moreover, the Adiol catabolite 3β,7β,17β-AT, which can be synthesized either by interconversion from 5-androstene-3β,7α,17β-triol or directly from Adiol by the catalytic activity of CYP3A4 and CYP3A7, is itself immunoprotective, despite its very low concentrations and high metabolic turnover [83].

Based on our previous data [33], we hypothesized that increased 7α-, 7β-, and 16α-hydroxylation play a role in the transition from adaptive immunity involving autoimmunity to the innate immune system involving inflammatory processes [7]. Nonetheless, synthetic anti-inflammatory derivatives of 3β,7β,17β-AT also suppress the production of inflammatory markers and pro-inflammatory cytokines [84].

Thus, the above data suggest that impaired 7α/β-hydroxylation of Δ^5^ androstanes may be involved in the pathophysiology of MS, impeding the formation of more potent immunomodulatory metabolites.

To summarize, the impaired 7α/β-hydroxylation of Δ^5^ androstanes, which appears to be primarily associated with anti-CD20 mAb, may, on the one hand, be associated with an increase in autoimmune response. On the other hand, a shift in the balance from the Th1 pattern toward the Th2 pattern resulting from the aforementioned impairment could attenuate acute inflammatory processes so that decreased 7α/β-hydroxylation of Δ^5^ androstanes may not be a clearly undesirable phenomenon in terms of MS severity.

#### 3.1.9. 5α-Reductases (SRD5As), 5β-Reductase (AKR1D1), and Aldoketoreductases Subfamily1c (AKR1C1-4)

The role of SRD5As, AKR1D1, and aldoketoreductases subfamily1C (AKR1C1-4) in relation to MS was discussed in detail in our previous study [33]. In summary, our current data mostly did not show significant trends in SMRs changes after treatment with anti-MS drugs, which may reflect the activity of these enzymes. Nevertheless, the SMRs that may reflect the activities of steroidogenic enzymes or balances between them indicate that S1PRI stimulates the activity of SDR5As in the FP. Also, the transcriptional analyses in the study by Noorbakhsh et al. showed significantly lower SRD5A1 transcripts in the white matter of MS patients postmortem compared to corresponding control samples [18]. Unfortunately, the authors did not specify the medication used.

Otherwise, our previous study reported that all indices of MS severity tended to correlate positively with 5α-reduced steroids [33], suggesting that these findings may also be related to the counter-regulatory effects of 5α-reduced steroids, which include a number of neuroprotective agents [18,19,48,61,64] (see also reviews [49,85,86]). Nevertheless, our previous study also showed that in contrast to the trend to positive correlations of 5α-reduced steroids with indices of MS severity, the SMRs that may reflect the functioning of SRD5As were unrelated to MS presence as well as to indices of MS severity. These results suggested that changes in SRD5As activities were not directly associated with MS pathophysiology. Therefore, the aforementioned attenuation of SDR5As activities may be related to the effect of treatment with S1PRI.

Various 5α/β-reduced metabolites of P and A are neuroprotective substances [87,88,89,90] that have anti-inflammatory effects; stimulate myelination and remyelination of Schwann cells in the peripheral nervous system; protect mitochondria; regulate neurogenesis; affect mood, memory, and cognition (see reviews [91,92]); and protect nervous system cells from hyperexcitation. The 5α/β-reduced C21 and C19 steroids are active at a number of receptors, including GABAA (γ-aminobutyric acid type A) and glutamate receptors (see review [92]).

On the one hand, S1PRI treatment resulted in a significant overall trend to decrease steroid levels and specifically for the levels of P 5α/β-reduced metabolites, but on the other, there was a significant trend to increase for SMSs that may reflect SRD5As and activity. Therefore, the tendency to stimulate the activity of both SRD5A by S1PRI may partially mitigate the adverse consequences of the overall trend of decreasing steroid levels after S1PRI medication, including 5α/β P metabolites.

SMRs suggest that AKR1D1 activity in LP may be stimulated by IFNβ-1a as well as ocrelizumab. Therefore, the tendency for stimulation of both SRD5As and AKR1D1 activities by the abovementioned anti-MS drugs may be beneficial for MS patients.

Concerning the balance between the activities of reductive AKR1C1 and oxidative HSD17B2 (as assessed from SMRs), the only effect was found for GA shifting the balance from the former to the latter enzyme. Our previous data did not find trends in correlations with neither indices of MS severity nor with MS presence [33], which was consistent with the results of transcriptional analysis of AKR1C isoforms in the study by Noorbakhsh et al. that also showed no significant alteration for the AKR1C1 transcripts in the white matter of patients when compared with controls [18]. Therefore, the aforementioned shift observed in our present data may be considered as the effect of GA therapy.

The relationship between GA therapy and a shift in the balance from the AKR1C1 to the HSD17B2 may be important as AKR1C1 suppresses lipid peroxidation of byproducts with catalytic efficiency comparable to that for 20α-hydroxysteroids and provides an inducible cytosolic barrier for 4-hydroxy-2-nonenal formed during lipid peroxidation after exposure to reactive oxygen species (ROS). In addition, AKR1C1 stimulation induces the activity of the transcription factor NF-E2-related factor-2 (NRF2), a key player in the regulation of antioxidant pathways [93]. Therefore, in terms of its effect on the steroidome, GA treatment may be adverse because it may be associated with the promotion of inflammatory processes accompanying MS.

The balance between reductive AKR1C2 on the one hand and oxidative HSD17B2 and oxidative and, at the same time, 3α/β isomerizingHSD17B6 on the other is of particular importance for neuroactive steroid biosynthesis, as AKR1C2 catalyzes the last step in the reduction of P to pregnanolone isomers, including the most important of them, 3α,5α-THP (allopregnanolone) and 3α,5β -THP (pregnanolone), which are neuro-inhibitory and neuroprotective GABAergic steroids [18,19,48,61,64] (see also reviews [49,85,86]).

Noorbakhsh et al. found that although there was no difference between MS patients and controls regarding pregnenolone, there was significantly lower 3α,5α-THP in the white matter of patients compared to controls [18]. However, these alterations were specific for 3α,5α-THP, while the remaining pregnanolone isomers, including GABAergic 3α,5β-THP, did not differ between MS patients and controls [18]. These observations were in accordance with data from our previous study [33] and were also consistent with data from transcriptional analysis of the same authors who found significantly lower levels of AKR1C2 transcript in the brains of MS patients compared to controls [18]. In addition, Noorbakhsh et al. also reported significantly lower mRNA immunoreactivity for AKR1C2 in MS patients compared to controls [61]. Concerning our present results, no significant effect of anti-MS treatment on SMRs reflecting the balance between activity of AKR1C2 and activities of HSD17B2 and HSD17B6 was found.

CNS inflammation and immune dysfunction play a key role in the pathogenesis of MS [1,49,86,94]. AKR1C3 (further enzyme from the AKR1C subfamily) is not only involved in steroidogenesis but is also effective as prostaglandin (PG) F2α synthase. At the same time, a highly active metabolite of PGF_2α_, 8-iso-prostaglandin F2α (8-iso-PGF2α), stimulates oxidative stress and contributes to the inflammatory process [95,96,97]. AKR1C3 is highly expressed in immunocompetent cells, adipose tissue, intestine, smooth muscle, bronchial cells, colon, and liver, but its expression has also been detected in adrenal *zona reticularis* and in a variety of other tissues [98,99,100], http://biogps.org/#goto=genereport&id=8644, accessed on 26 December 2024) (see also reviews [101,102,103]).

Our previous study showed a borderline trend toward higher values of the SMRs that may reflect the balance between reductive AKR1C3 and oxidative HSD17B2 in MS patients. This may contribute to the increased incidence of inflammatory responses in MS patients [33]. Our current data suggested that ofatumumab in the FP (observed significant trend to decrease after treatment) and ocrelizumab in the LP (insignificant decrease after treatment) may shift a balance from the reductive proinflammatory AKR1C3 to oxidative HSD17B2. This data may indicate an attenuation of pro-inflammatory milieu associated with lower AKR1C3 activity after treatment with humanized anti-CD20 monoclonal antibodies.

### 3.2. Potential Clinical Implications of the Findings

This study can be considered the first attempt to systematically analyze the relationship between endogenous steroids and anti-MS drug treatment. The findings may be important concerning alterations in circulating bioactive steroids, such as cortisol, active androgens and estrogens, and neuroactive, neuroprotective, and immunomodulatory substances in terms of optimization of the MS treatment.

### 3.3. Future Directions

Respecting the use of SMRs as surrogate markers for estimation of functioning of steroidogenic enzymes, further research should focus on direct assessment of the enzyme activities and expression of respective steroidogenic enzymes. Also, in respect of insufficient data for some anti-MS drugs in our study, future studies should focus on individual anti-MS drugs with sufficient numbers of volunteers.

### 3.4. Limitations of the Study

The reported study results were focused on steroidomics only. Thus, the assessments of changes in steroidogenic enzyme functioning after anti-MS used only the steroidomic surrogate markers based on SMRs, and the changes in activities of steroidogenic enzymes were not directly tested.

Although this study includes a number of steroids and covers most of the steroid metabolic pathways, the study does not include 11-deoxycortisol and 11-deoxy-corticosterone.

In addition, the number of patients included in the present study was satisfactory for the estimation of pooled trends, but there was insufficient data concerning some of the individual anti-MS drugs; therefore, the respective results are preliminary. Nevertheless, despite the limited sample size, our patient population was homogeneous, i.e., included only Caucasian patients due to the demography of the Czech Republic.

## 4. Materials and Methods

### 4.1. Subjects

A total of 61 female MS patients of reproductive age 39 (29, 47) years was included in this study (shown as median with quartiles). The diagnosis MS was confirmed by cerebrospinal fluid analysis and magnetic resonance imaging. All of the MS patients fulfilled the revised 2017 McDonald criteria [104]. The MS patients included in this study had just been diagnosed and had not yet been treated. Patients who experienced COVID were not included in the present study.

The study was approved by the Ethics Committee of the General University Hospital, Prague, Czech Republic (Approval number: 74/19 Grant AZV VES 2020 VFN, 20 June 2019), and all procedures involving human subjects were conducted following ethical standards set by national and institutional committees on human experimentation and the Helsinki Declaration of 1975, as updated in 2008. The authors guarantee that all research procedures were carried out with the utmost respect for the participant’s safety, well-being, and confidentiality. Participants were examined after signing an informed consent approved by the aforementioned Ethics Committee. For the evaluation of steroidome, the peripheral blood was withdrawn on fasting in the morning. Blood samples were centrifuged and stored at −20 °C until analyzed.

### 4.2. Steroid Analyses

Steroids and their polar conjugates were measured using our previously described validated GC-MS/MS method [105] with the exception of E2, which was quantified using Electro-chemiluminescence immunoassays (ECLIA), performed on Cobas^®^ Pro, Roche Diagnostics International Ltd. (Rotkreuz, Switzerland). In addition to the use of GC-MS/MS, cortisol was also measured using the RIA cortisol kit from Beckmann Coulter, Czech Republic, Ltd., Praha, Czech Republic.

### 4.3. Statistical Analyses

In the first step, the power transformation parameters were found for each metric variable so that its distribution was as close as possible to the Gaussian distribution. The steroidomic data were evaluated using an ANOVA model as well as multivariate regression with reduced dimensionality known as orthogonal projections to latent structure (OPLS) model. The ANOVA consisted of subject factors separating the inter-individual variability, factor of menstrual phase (PMC, follicular vs. luteal), stage factor (Stage 0, before treatment vs. Stage 1, after treatment), and PMC × Stage interaction. Statgraphics Centurion v. XVIII statistical software from Statgraphics Technologies, Inc. (The Plains, VA, USA) was used for power transformations of the original data and for evaluation using the ANOVA model, while SIMCA-P version 12.0 statistical software from Umetrics AB (Umeå, Sweden) was used for OPLS analysis.

The OPLS models were focused on the differentiation between naïve MS patients and those treated with individual anti-MS drugs. In terms of diagnosing MS based on highly intercorrelated steroidomic data, it was appropriate to use OPLS models that examined the correlation of MS with multiple parameters simultaneously. The OPLS model, which is a multivariate regression with dimensionality reduction, permits the evaluation of relationships between explanatory variables and a number of explanatory variables that may be highly correlated, which is also the case for steroids in metabolic pathways. The use of the given anti-MS drug is expressed in the OPLS model as the logarithm of the likelihood ratio (the ratio of the probability of its use *p* to the probability of its absence (1 − *p*)), i.e., the logarithm of the likelihood ratio is calculated, which then ranges from −infinity to +infinity. This approach ensures that the prediction of the probability of the presence of pathology is between 0 and 1 (after using a recurrent formula that converts the logarithm of the likelihood ratio to the probability of the presence of pathology).

The variability of the explaining and explained variables is separated into two independent components in the OPLS. The former contains the variability in explaining variables that were shared with the probability of pathology (predictive component), while the orthogonal components express the variability shared between highly correlated explaining variables (orthogonal components). OPLS identifies significant explanatory variables and their best linear combination to estimate the probability of the presence of pathology. After standardizing the variables, the OPLS model can be expressed as follows:

(1) $$X=T_{p}P_{p}^{T}+T_{0}P_{0}^{T}+E$$

(2) $$Y=T_{p}P_{p}^{T}+F$$

where **X** is the matrix with predictors and subjects; **Y** is the vector of dependent variable and subjects; **T**_p_ is the vector of component scores from the single predictive component and subjects extracted from **Y**; **T**_o_ is the vector of component scores from the single orthogonal component and subjects extracted from **X**; **P**_p_ is the vector of component loadings for the predictive component extracted from **Y**; **P**_o_ is the vector of component loadings for the orthogonal component extracted from **X** and independent variables; and **E** and **F** are the error terms.

Significant predictors were selected using the variable importance statistics (VIP). The statistical software SIMCA-P v.12.0 from Umetrics AB (Umeå, Sweden), which was used for OPLS analysis, enabled finding the number of relevant components, the detection of multivariate non-homogeneities, and testing the multivariate normal distribution and homoscedasticity (constant variance).

The algorithm for obtaining the predictions were as follows:

- Transformation of the original data to obtain the values with symmetric distribution and constant variance.
- Checking the data homogeneity in predictors using Hotelling’s statistics and the eventual elimination of non-homogeneities.
- Testing the relevance of predictors using variable importance statistics and the elimination of irrelevant predictors.
- Calculating component loadings for individual variables to evaluate their correlations with the predictive component.
- Calculating regression coefficients for the ordinary multiple regression model (OMR) to evaluate the mutual independence of predictors after comparison with the corresponding component loadings from the OPLS model.
- Calculating predicted values of the logarithm of the ratio of the probability of pathology presence to the probability of pathology absence (LLR).
- Calculating the probability of the pathology presence for individual subjects.
- Calculating the sensitivity and specificity of the prediction.

The ratio between significantly positive, missing, and significantly negative correlations with MS was evaluated using the one-sample Wilcoxon test with correction for continuity.

## 5. Conclusions

In conclusion:

(1) A comprehensive steroidomic analysis of steroidomic changes after treatment with anti-MS drugs was performed. The study participants were newly diagnosed MS female patients who met the 2017 revised McDonald criteria and had not yet been treated.
(2) Almost all steroids have been studied for the first time in terms of the effects of treatment with anti-MS drugs.
(3) MS patients showed a general tendency toward a decrease in steroid levels in follicular menstrual phase due to treatment with anti-MS drugs, but absence of this trend in the luteal phase, which could be interpreted as a weakening of counterregulatory mechanisms associated with adrenal steroids, which may mitigate adverse effects of MS. Another explanation could be the anti-steroidogenic effect of key anti-MS drugs on adrenal steroidogenesis.
(4) Decreased 17-hydroxy-pregnane levels after treatment with anti-MS drugs in FP indicate a decreasing trend of CYP17A1 activity, suggesting a weakening of one of the steps of the cortisol pathway and, together with the analysis for individual anti-MS drugs, a strengthening of the step toward immunomodulatory adrenal androgens. These results also suggest that among the anti-MS drugs, the monoclonal anti-CD20, S1PRI, and IFNβ-1a antibodies may be responsible for the above findings. These findings could have implications for the synthesis and metabolism of bioactive steroids, whether they are cortisol, active androgens and estrogens, or neuroactive and neuroprotective substances acting through modulation of ionotropic receptors.
(5) A significant trend to decreased ratios of conjugated steroids to their unconjugated counterparts was observed, indicating either suppression of SULT2A1 activity and/or stimulation of STS activity by anti-MS drugs, from which GA and ofatumumab in FP and ocrelizumab in both menstrual phases contributed to this pooled trend. Moreover, our current data show a significant trend toward a decrease in conjugated steroid levels in FP after anti-MS drug therapy. The above data may be relevant to the regulation of neuronal excitability because:
  - Unconjugated steroids act through binding to nuclear receptors while their sulfates are inactive.
  - Sulfation of unconjugated neuroinhibitory GABAergic steroids leads to their inactivation or even to the formation of their antagonists.
  - Some sulfated steroids can be positive or negative modulators of excitatory glutamate receptors, whereas all their unconjugated analogues are inactive in this context.
  - Sulfated androgens may serve as a reservoir of substrates for the synthesis of bioactive androgens acting as sex hormones, neuroprotective and immunomodulatory agents.
(6) A borderline downward trend was observed in CYP11B1 after anti-MS therapy, which may downregulate cortisol synthesis in the last metabolic step.

As for further changes in steroid levels after treatment with individual anti-MS drugs:

(1) GA treatment in FP also shifted the balance from reductive AKR1C1 to oxidative HSD17B2, which may contribute to the inflammatory milieu.
(2) In addition to affecting CYP17A1 activities, IFN-β1a also showed:
  - A significant trend toward increased steroidogenesis in LP, which is predominantly controlled by ovarian activity but no effect in FP, which is predominantly controlled by adrenal activity.
  - A significant trend toward increased steroid levels after IFN-β1a in LP included significant increases in some important bioactive steroids, such as the neuroprotective pregnenolone and steroid hormones, such as testosterone, 5α-DHT, and cortisol. These observations indicate a beneficial effect of IFN-β1a treatment in terms of MS severity.
  - A significant trend to higher SMRs that may reflect AKR1D1 activity, which could be of importance in terms of the synthesis of neuroinhibitory 5β-pregnanes.
(3) S1PRI (in addition to affecting a general trend in steroid levels and SMRs that may reflect CYP17A1 activities) also showed a trend to lower SMRs that may reflect SRD5A activity.
(4) Anti-CD20 mAb (ofatumumab, ocrelizumab) treatment (in addition to affecting a general trend in steroid levels and SMRs that may reflect CYP17A1 activities and shift in the balance conjugated/unconjugated steroid) showed:
  - A borderline trend to decrease for the SMRs that may reflect the activities of HSD3Bs (ofatumumab).
  - A significant (ofatumumab) or borderline (ocrelizumab in LP) trend, or at least indication of the trend (ocrelizumab in LP) to lower values of SMRs that may reflect activities of CYP7B1, CYP3A4, and CYP3A7 enzymes catalyzing the synthesis of immunomodulatory 7α/β and 16α-hydroxy-androgens. Moreover, there was a pooled borderline downward trend in their levels after therapy with anti-MS drugs. These data are relevant concerning the immunomodulatory effect of these steroids as they affect Th1/Th2 balance and thus may influence the severity of MS.
  - An indication of the trend to decrease in SMRs that may reflect the activity of reductive AKR1C3 (primarily converting 17-oxo androstanes and estrogens to their 17 β-hydroxy counterparts) to oxidative HSD17B2 activity acting in the opposite direction for ocrelizumab in the LP and even the significant trend for ofatumumab.
  - A significant trend to lower values of the SMRs that may reflect the activity of AKR1D1 (ocrelizumab in the LP).

## Figures and Tables

**Table 1:** Trends in steroids SMRs (that may reflect activities of steroidogenic enzymes and balances between them) after treatment with anti-MS drugs. The data in Table 1 represent numbers of steroids or molar ratios with a significant decrease in values (↓), no significant change (~), or significant increase in values after treatment (↑).

Steroids and Steroid Molar Ratios	Trend	Follicular Phase	Luteal Phase	Steroids and Steroid Molar Ratios	Trend	Follicular Phase	Luteal Phase	Steroids and Steroid Molar Ratios	Trend	Follicular Phase	Luteal Phase	Steroids and Steroid Molar Ratios	Trend	Follicular Phase	Luteal Phase
Steroids	↓	***12***	*0*	17-oxo-Androstanes/ estrogens	↓	*2*	*0*	CYP17A1, hydroxylase	*↓*	*4*	*1*	AKR1D1	*↓*	*1*	*0*
	~	***66***	*78*		~	*19*	*21*		*~*	*8*	*12*		*~*	*10*	*13*
	↑	***1***	*1*		↑	*0*	*0*		*↑*	*1*	*0*		*↑*	*2*	*0*
	*p*	***0.002***	*0.323*		*p*	*0.168*	*1*		*p*	*0.193*	*0.356*		*p*	*0.176*	*1*
Unconjugated steroids	↓	6	*0*	17β-hydroxy Androstanes + estrogens	↓	*1*	*0*	CYP17A1, lyase	*↓*	*1*	*0*	AKR1C1 vs. HSD17B2	*↓*	*5*	*0*
	~	38	*44*		~	*14*	*14*		*~*	*7*	*13*		*~*	*9*	*16*
	↑	1	*1*		↑	*0*	*1*		*↑*	*5*	*0*		*↑*	*3*	*1*
	*p*	0.06	*0.328*		*p*	*0.351*	*0.351*		*p*	*0.111*	*1*		*p*	*0.496*	*0.347*
Conjugated steroids	↓	***6***	*0*	20-oxo-Pregnanes	↓	*5*	*0*	HSD3B	*↓*	*1*	*0*	AKR1C2 vs. HSD17B2 and HSD17B6	*↓*	*2*	*2*
	~	***28***	*34*		~	*17*	*23*		*~*	*10*	*11*		*~*	*18*	*18*
	↑	***0***	*0*		↑	*1*	*0*		*↑*	*0*	*0*		*↑*	*0*	*0*
	*p*	***0.015***	*1*		*p*	*0.107*	*1*		*p*	*0.363*	*1*		*p*	*0.168*	*0.168*
C21 steroids (pregnanes)	↓	***9***	*0*	20α-Hydroxy-pregnanes	↓	***4***	*0*	Conjugated/ unconjugated steroids	*↓*	***7***	*0*	AKR1C2 vs. HSD17B2 and HSD17B6, 3α/β-hydroxy steroids	*↓*	*2*	*2*
	~	***33***	*44*		~	***16***	*20*		*~*	***19***	*26*		*~*	*11*	*11*
	↑	***1***	*0*		↑	***0***	*0*		*↑*	***0***	*0*		*↑*	*0*	*0*
	*p*	***0.012***	*1*		*p*	***0.049***	*1*		*p*	***0.009***	*1*		*p*	*0.175*	*0.175*
C19 steroids (androstanes)	↓	3	*0*	3α-Hydroxy-5α/β-pregnanes	↓	4	*0*	CYP11B1	*↓*	*2*	4	AKR1C2 vs. HSD17B2 and HSD17B6, 3α-hydroxy/3-oxo steroids	*↓*	*0*	*0*
	~	31	*33*		~	11	*15*		*~*	*5*	3		*~*	*7*	*7*
	↑	0	*1*		↑	0	*0*		*↑*	*0*	0		*↑*	*0*	*0*
	*p*	0.086	*0.332*		*p*	0.05	*1*		*p*	*0.192*	0.056		*p*	*1*	*1*
Corticoids and 11β-hydroxy-androstanes	↓	4	*0*	3-oxo/3β-Hydroxy-5α/β-pregnanes	↓	*1*	*0*	CYP7B1, 3A4	*↓*	4	*0*	AKR1C3	*↓*	*2*	*0*
	~	7	*11*		~	*12*	*14*		*~*	4	*8*		*~*	*8*	*12*
	↑	0	*0*		↑	*1*	*0*		*↑*	0	*0*		*↑*	*2*	*0*
	*p*	0.051	*1*		*p*	*1*	*1*		*p*	0.054	*1*		*p*	*1*	*1*
17-Hydroxy-pregnanes	↓	***6***	*0*	7α/β- and 16α-Hydroxy-steroids	*-1*	*2*	*0*	HSD11B1	*↓*	*0*	*0*				
	~	***8***	*14*		*0*	*6*	*8*		*~*	*4*	*4*				
	↑	***0***	*0*		*1*	*0*	*0*		*↑*	*0*	*0*				
	*p*	***0.016***	*1*		*p*	*0.187*	*1*		*p*	*1*	*1*				
17-Deoxy-pregnanes	↓	*3*	*0*	CYP17A1, hydroxylase + lyase	*↓*	*3*	*0*	SRD5A	*↓*	*1*	*0*				
	~	*25*	*29*		*~*	*18*	*23*		*~*	*9*	*13*				
	↑	*1*	*0*		*↑*	*2*	*0*		*↑*	*3*	*0*				
	*p*	*0.326*	*1*		*p*	*0.67*	*1*		*p*	*0.339*	*1*				

**Table 2:** Effect of anti-MS drugs on steroidomic data, including SMRs (which may reflect activities of steroidogenic enzymes and balances between them). The data in Table 2 represent numbers of steroids or molar ratios with a significant decrease in values (↓), no significant change (~), or significant increase in values after treatment (↑).

Steroids and Steroid Molar Ratios	Trend	GA, Follicular Phase	IFNβ-1a, Follicular Phase	IFNβ-1a, Luteal Phase	S1PR Inhibitors, Follicular Phase	Ofatumumab, Follicular Phase	Ocrelizumab, Follicular Phase	Ocrelizumab, Luteal Phase
Steroids	↓	*0*	*6*	**0**	**11**	**17**	**16**	**20**
	~	*79*	*71*	**58**	**67**	**62**	**62**	**56**
	↑	*0*	*2*	**21**	**1**	**0**	**1**	**3**
	*p*	*1*	*0.159*	**<0.001**	**0.004**	**<0.001**	**<0.001**	**<0.001**
Corticoids and 11β-hydroxy-androgens	↓	*0*	*1*	**0**	*2*	*1*	*0*	**5**
	~	*11*	*10*	**6**	*9*	*10*	*10*	**6**
	↑	*0*	*0*	**5**	*0*	*0*	*1*	**0**
	*p*	*1*	*0.363*	**0.029**	*0.178*	*0.363*	*0.363*	**0.029**
Δ^5^ and Δ^4^ steroids	↓	*0*	3	**0**	3	**8**	6	6
	~	*11*	27	**20**	27	**22**	23	23
	↑	*0*	0	**10**	0	**0**	1	1
	*p*	*1*	0.087	**0.002**	0.087	**0.005**	0.061	0.061
Progesterone 5α/β-reduced metabolites	↓	*0*	3	**0**	**7**	**6**	**7**	*5*
	~	*26*	23	**20**	**18**	**20**	**19**	*19*
	↑	*0*	0	**6**	**1**	**0**	**0**	*2*
	*p*	*1*	0.087	**0.015**	**0.035**	**0.015**	**0.009**	*0.264*
CYP17A1	↓	*0*	*1*	*0*	1	*0*	*0*	**8**
	~	*23*	*18*	*23*	16	*23*	*23*	**15**
	↑	*0*	*4*	*0*	6	*0*	*0*	**0**
	*p*	*1*	*0.187*	*1*	0.061	*1*	*1*	**0.005**
CYP17A1, hydroxylase	↓	*0*	3	*1*	3	*5*	**6**	**7**
	~	*13*	10	*11*	10	*6*	**7**	**6**
	↑	*0*	0	*1*	0	*2*	**0**	**0**
	*p*	*1*	0.092	*1*	0.092	*0.273*	**0.016**	**0.009**
CYP17A1, lyase	↓	*0*	**0**	*0*	*0*	*1*	**0**	*0*
	~	*11*	**8**	*13*	*13*	*8*	**6**	*13*
	↑	*2*	**5**	*0*	*0*	*4*	**7**	*0*
	*p*	*0.175*	**0.028**	*1*	*1*	*0.193*	**0.009**	*1*
HSD3Bs	↓	*0*	*0*	*0*	*0*	4	*0*	*0*
	~	*11*	*11*	*11*	*11*	7	*11*	*9*
	↑	*0*	*0*	*0*	*0*	0	*0*	*2*
	*p*	*1*	*1*	*1*	*1*	0.051	*1*	*0.178*
Conjugated/ unconjugated steroids	↓	**6**	*3*	*0*	*4*	*5*	**4**	**6**
	~	**20**	*22*	*26*	*21*	*20*	**22**	**20**
	↑	**0**	*1*	*0*	*1*	*1*	**0**	**0**
	*p*	**0.015**	*0.327*	*1*	*0.186*	*0.106*	**0.048**	**0.015**
CYP11B1	↓	*0*	*0*	*0*	*0*	*0*	*0*	*0*
	~	*7*	*7*	*7*	*7*	*7*	*7*	*7*
	↑	*0*	*0*	*0*	*0*	*0*	*0*	*0*
	*p*	*1*	*1*	*1*	*1*	*1*	*1*	*1*
CYP7B1, CYP3A4, CYP3A7	↓	*0*	*0*	*0*	*2*	**5**	3	4
	~	*8*	*8*	*8*	*5*	**3**	5	4
	↑	*0*	*0*	*0*	*1*	**0**	0	0
	*p*	*1*	*1*	*1*	*0.621*	**0.031**	0.099	0.054
HSD11B1	↓	*0*	*0*	*0*	*0*	*2*	*1*	*0*
	~	*4*	*4*	*4*	*4*	*1*	*2*	*4*
	↑	*0*	*0*	*0*	*0*	*1*	*1*	*0*
	*p*	*1*	*1*	*1*	*1*	*0.7*	*1*	*1*
SRD5As	↓	*0*	*0*	*0*	**0**	*1*	*1*	*0*
	~	*13*	*13*	*13*	**8**	*9*	*9*	*13*
	↑	*0*	*0*	*0*	**5**	*3*	*3*	*0*
	*p*	*1*	*1*	*1*	**0.028**	*0.339*	*0.339*	*1*
AKR1D1	↓	*0*	*0*	**0**	*4*	*1*	*0*	**7**
	~	*13*	*13*	**8**	*8*	*8*	*13*	**6**
	↑	*0*	*0*	**5**	*1*	*4*	*0*	**0**
	*p*	*1*	*1*	**0.028**	*0.193*	*0.193*	*1*	**0.009**
AKR1C1 vs. HSD17B2	↓	**6**	*3*	*2*	*5*	*3*	*0*	*2*
	~	**11**	*12*	*12*	*10*	*13*	*17*	*15*
	↑	**0**	*2*	*3*	*2*	*1*	*0*	*0*
	*p*	**0.015**	*0.676*	*0.676*	*0.268*	*0.333*	*1*	*0.17*
AKR1C2 vs. HSD17B2 and HSD17B6	↓	*3*	*2*	*3*	*2*	*3*	*2*	*0*
	~	*15*	*16*	*17*	*14*	*15*	*15*	*21*
	↑	*3*	*3*	*1*	*5*	*3*	*4*	*0*
	*p*	*1*	*0.672*	*0.33*	*0.266*	*1*	*0.427*	*1*
AKR1C2 vs. HSD17B2 and HSD17B6, 3α/β-hydroxy steroids	↓	*3*	*1*	3	*2*	*3*	*2*	*0*
	~	*9*	*10*	11	*10*	*10*	*9*	*14*
	↑	*2*	*3*	0	*2*	*1*	*3*	*0*
	*p*	*0.682*	*0.337*	0.091	*1*	*0.337*	*0.682*	*1*
AKR1C2 vs. HSD17B2 and HSD17B6, 3α-hydroxy/3-oxo ster	↓	*0*	*1*	*0*	*0*	*0*	*0*	*0*
	~	*6*	*6*	*6*	*4*	*5*	*6*	*7*
	↑	*1*	*0*	*1*	*3*	*2*	*1*	*0*
	*p*	*0.391*	*0.391*	*0.391*	*0.102*	*0.192*	*0.391*	*1*
AKR1C3 vs. HSD17B2	↓	*0*	*0*	*0*	*0*	**5**	*0*	3
	~	*12*	*12*	*12*	*12*	**7**	*12*	9
	↑	*0*	*0*	*0*	*0*	**0**	*0*	0
	*p*	*1*	*1*	*1*	*1*	**0.028**	*1*	0.093

*p* = *p*-value, ↓ = trend to decrease, ~ = absent trend, ↑ = trend to increase, *p* < 0.05 (bold), *p* < 0.1 (normal), and *p* > 0.1 (italics).

## Data Availability

The data presented in this study are available on request from the corresponding author. The data is not publicly available due to the future use of part of it in another study.

## Supplementary Materials

The following supporting information can be downloaded at: https://www.mdpi.com/article/10.3390/ijms26051835/s1.

## Abbreviations

11β-OH-3α,5α-THA	11β-Hydroxyandrosterone
11β-OH-A	11β-hydroxyandrostenedione
16α-OH-P	16α-Hydroxyprogesterone
16α-OH-Preg	16α-Hydroxypregnenolone
17-OH-P	17-Hydroxyprogesterone
17-OH-Preg	17-Hydroxypregnenolone
17-OH-20α-DHP	17α-Hydroxy-20α-dihydroprogesterone
5α-DHA	5α-Androstane-3,17-dione
5α-DHT	5α-Dihydrotestosterone
3α,5α,17-PD	17-Hydroxyallopregnanolone
3α,5α,17,20α-PT	5α-Pregnane-3α,17,20α-triol
3α,5α,20α-PD	5α-Pregnane-3α, 20α-diol
3α,5α-THA	Androsterone
3α,5α-THP	3α,5α-Tetrahydroprogesterone, allopregnanolone
3β,5α,17,20α-PT	5α-Pregnane-3β,17,20α-triol
3β,5α-THA	Epiandrosterone
3α,5β,17,20α-PT	5β-Pregnane-3α,17,20α-triol
3α,5β,17-PD	17-Hydroxypregnanolone
3α,5β-THA	Etiocholanolone
3α,5β-THA	Epietiocholanolone
8-iso-PGF2α	8-iso-Prostaglandin F_2α_
σ_1_Rs	Sigma-1 receptors
A	Androstenedione
ACTH	Adrenocorticotropic hormone
Adiol	Androstenediol
AKR1C1	Aldo-keto reductase family 1 member C1
AKR1C2	Aldo-keto reductase family 1 member C2
AKR1C3	Aldo-keto reductase family 1 member C3
AKR1D1	5β-Reductase
AMPARs	α-Amino-3-hydroxy-5-methyl-4-isoxazolepropionic acid receptors
ANOVA	Analysis of variance
AT	Androstenetriol (5-androstene)
BBB	Blood-brain barrier
CNS	Central nervous system
CRH	Corticotropin-releasing hormone
CYP11B1	11β-Hydroxylase
CYP17A1	C17-hydroxylase-C17,20-lyase
CYP19A1	Aromatase
CYP21A2	21-Hydroxylase
CYP3A4	Cytochrome P450 3A4
CYP3A7	Cytochrome P450 3A7
CYP7B1	7α-hydroxylase
DHEA	Dehydroepiandrosterone
DHEAC	Dehydroepiandrosterone sulfate
DHP	Dihydroprogesterone
EDSS	Expanded Disability Status Scale
FSH	Follicle-stimulating hormone
GABA_A_Rs	Type A γ-aminobutyric acid receptors
GR	Glucocorticoid receptors
GC-MS/MS	Gas chromatography tandem mass spectrometry
HPAA	Hypothalamic-pituitary-adrenal axis
HSD3B1	Type 1 3β-Hydroxysteroid dehydrogenase
HSD3B2	Type 2 3β-Hydroxysteroid dehydrogenase
HSD3Bs	Type 1 and 2 3β-Hydroxysteroid dehydrogenases
HSD11B1	Type 1 11β-Hydroxysteroid dehydrogenase
HSD11B2	Type 2 11β-Hydroxysteroid dehydrogenase
HSD17B2	Type 2 17β-Hydroxysteroid dehydrogenase
HSD17B6	Type 6 17β-Hydroxysteroid dehydrogenase (3α/β epimerase)
HPT9_R	9-Hole Peg Test for right hand
HPT9_L	9-Hole Peg Test for left hand
IFN-γ	Interferon γ
IL-1	Interleukin 1
IL-2	Interleukin 2
IL-4	Interleukin 4
IL-6	Interleukin 6
IL-10	Interleukin 10
IL-12	Interleukin 12
IL-17	Interleukin 17
LLR	Logarithm of likelihood ratio
LSD	Least significant difference
mRNA	Messenger ribonucleic acid
NMDARs	N-methyl-D-aspartate receptors
OPLS	Orthogonal predictions to latent structure
P	Progesterone
PD	Pregnanediol
PGF_2α_	Prostaglandin F_2α_
PMC	Phase of menstrual cycle
PPMS	Primary progressive multiple sclerosis
Preg	Pregnenolone
PregC	Pregnenolone sulfate
PT	Pregnanetriol
RIA	Radioimmunoassay
ROS	Reactive oxygen species
RRMS	Relapsing-remitting multiple sclerosis
SRD5A1	Type 1 5α-reductase
SRD5As	5α-Reductases
SULT2A1	Steroid sulfotransferase type 2A1
STS	Steroid sulfatase
T	Testosterone
T25FWT	Timed 25-foot walk
THP	Tetrahydroprogesterone
TNF	Tumor necrosis factor
TRPC5s	Short transient receptor potential channels 5
TRPV1s	Vanilloid receptors
TRPM3s	Melastatin receptors
The letter C after the abbreviation of a steroid indicates its conjugated form.	
The “C” after the molar ratio of a steroid indicates that they are its conjugated forms.	

## References

[1] Ysrraelit M.C., Correale J. (2019). Impact of sex hormones on immune function and multiple sclerosis development. Immunology.

[2] Sparaco M., Bonavita S. (2021). The role of sex hormones in women with multiple sclerosis: From puberty to assisted reproductive techniques. Front. Neuroendocrinol..

[3] Smith R., Studd J.W. (1992). A pilot study of the effect upon multiple sclerosis of the menopause, hormone replacement therapy and the menstrual cycle. J. R. Soc. Med..

[4] Argyriou A.A., Makris N. (2008). Multiple sclerosis and reproductive risks in women. Reprod. Sci..

[5] Trombetta A.C., Meroni M., Cutolo M. (2017). Steroids and Autoimmunity. Front. Horm. Res..

[6] Kamin H.S., Kertes D.A. (2017). Cortisol and DHEA in development and psychopathology. Horm. Behav..

[7] Mikulska J., Juszczyk G., Gawronska-Grzywacz M., Herbet M. (2021). HPA Axis in the Pathomechanism of Depression and Schizophrenia: New Therapeutic Strategies Based on Its Participation. Brain Sci..

[8] Cherian K., Schatzberg A.F., Keller J. (2019). HPA axis in psychotic major depression and schizophrenia spectrum disorders: Cortisol, clinical symptomatology, and cognition. Schizophr. Res..

[9] Misiak B., Piotrowski P., Chec M., Samochowiec J. (2021). Cortisol and dehydroepiandrosterone sulfate in patients with schizophrenia spectrum disorders with respect to cognitive performance. Compr. Psychoneuroendocrinol..

[10] Ritsner M., Maayan R., Gibel A., Strous R.D., Modai I., Weizman A. (2004). Elevation of the cortisol/dehydroepiandrosterone ratio in schizophrenia patients. Eur. Neuropsychopharmacol..

[11] Ritsner M., Gibel A., Maayan R., Ratner Y., Ram E., Modai I., Weizman A. (2007). State and trait related predictors of serum cortisol to DHEA(S) molar ratios and hormone concentrations in schizophrenia patients. Eur. Neuropsychopharmacol..

[12] Kancheva R., Hill M., Novak Z., Chrastina J., Kancheva L., Starka L. (2011). Neuroactive steroids in periphery and cerebrospinal fluid. Neuroscience.

[13] Begemann M.J., Dekker C.F., van Lunenburg M., Sommer I.E. (2012). Estrogen augmentation in schizophrenia: A quantitative review of current evidence. Schizophr. Res..

[14] Qaiser M.Z., Dolman D.E.M., Begley D.J., Abbott N.J., Cazacu-Davidescu M., Corol D.I., Fry J.P. (2017). Uptake and metabolism of sulphated steroids by the blood-brain barrier in the adult male rat. J. Neurochem..

[15] Cai H., Cao T., Zhou X., Yao J.K. (2018). Neurosteroids in Schizophrenia: Pathogenic and Therapeutic Implications. Front. Psychiatry.

[16] Powrie Y.S.L., Smith C. (2018). Central intracrine DHEA synthesis in ageing-related neuroinflammation and neurodegeneration: Therapeutic potential?. J. Neuroinflammation.

[17] Honcu P., Hill M., Bicikova M., Jandova D., Velikova M., Kajzar J., Kolatorova L., Bestak J., Macova L., Kancheva R. (2019). Activation of Adrenal Steroidogenesis and an Improvement of Mood Balance in Postmenopausal Females after Spa Treatment Based on Physical Activity. Int. J. Mol. Sci..

[18] Noorbakhsh F., Ellestad K.K., Maingat F., Warren K.G., Han M.H., Steinman L., Baker G.B., Power C. (2011). Impaired neurosteroid synthesis in multiple sclerosis. Brain.

[19] Noorbakhsh F., Baker G.B., Power C. (2014). Allopregnanolone and neuroinflammation: A focus on multiple sclerosis. Front. Cell Neurosci..

[20] Cil A.P., Leventoglu A., Sonmezer M., Soylukoc R., Oktay K. (2009). Assessment of ovarian reserve and Doppler characteristics in patients with multiple sclerosis using immunomodulating drugs. J. Turk. Ger. Gynecol. Assoc..

[21] Griffiths W.J., Wang Y. (2021). Sterols, Oxysterols, and Accessible Cholesterol: Signalling for Homeostasis, in Immunity and During Development. Front. Physiol..

[22] Sukocheva O., Wadham C., Gamble J., Xia P. (2015). Sphingosine-1-phosphate receptor 1 transmits estrogens’ effects in endothelial cells. Steroids.

[23] Lucki N.C., Li D., Sewer M.B. (2012). Sphingosine-1-phosphate rapidly increases cortisol biosynthesis and the expression of genes involved in cholesterol uptake and transport in H295R adrenocortical cells. Mol. Cell Endocrinol..

[24] Guzman A., Rosales-Torres A.M., Medina-Moctezuma Z.B., Gonzalez-Aretia D., Hernandez-Coronado C.G. (2024). Effects and action mechanism of gonadotropins on ovarian follicular cells: A novel role of Sphingosine-1-Phosphate (S1P). A review. Gen. Comp. Endocrinol..

[25] Siavoshi F., Ladakis D.C., Muller A., Nourbakhsh B., Bhargava P. (2024). Ocrelizumab alters the circulating metabolome in people with relapsing-remitting multiple sclerosis. Ann. Clin. Transl. Neurol..

[26] Ito K., Ito N., Yadav S.K., Suresh S., Lin Y., Dhib-Jalbut S. (2021). Effect of switching glatiramer acetate formulation from 20 mg daily to 40 mg three times weekly on immune function in multiple sclerosis. Mult. Scler. J. Exp. Transl. Clin..

[27] Dargahi N., Katsara M., Tselios T., Androutsou M.E., de Courten M., Matsoukas J., Apostolopoulos V. (2017). Multiple Sclerosis: Immunopathology and Treatment Update. Brain Sci..

[28] Murdoch D., Lyseng-Williamson K.A. (2005). Spotlight on subcutaneous recombinant interferon-beta-1a (Rebif) in relapsing-remitting multiple sclerosis. BioDrugs.

[29] Giovannoni G., Munschauer F.E., Deisenhammer F. (2002). Neutralising antibodies to interferon beta during the treatment of multiple sclerosis. J. Neurol. Neurosurg. Psychiatry.

[30] Hauser S.L., Kappos L., Bar-Or A., Wiendl H., Paling D., Williams M., Gold R., Chan A., Milo R., Das Gupta A. (2023). The Development of Ofatumumab, a Fully Human Anti-CD20 Monoclonal Antibody for Practical Use in Relapsing Multiple Sclerosis Treatment. Neurol. Ther..

[31] McGinley M.P., Moss B.P., Cohen J.A. (2017). Safety of monoclonal antibodies for the treatment of multiple sclerosis. Expert. Opin. Drug Saf..

[32] Lamb Y.N. (2022). Ocrelizumab: A Review in Multiple Sclerosis. Drugs.

[33] Kancheva R., Hill M., Velikova M., Kancheva L., Vcelak J., Ampapa R., Zido M., Stetkarova I., Libertinova J., Vosatkova M. (2024). Altered Steroidome in Women with Multiple Sclerosis. Int. J. Mol. Sci..

[34] Labrie F., Martel C., Belanger A., Pelletier G. (2017). Androgens in women are essentially made from DHEA in each peripheral tissue according to intracrinology. J. Steroid Biochem. Mol. Biol..

[35] Angeli A., Masera R.G., Sartori M.L., Fortunati N., Racca S., Dovio A., Staurenghi A., Frairia R. (1999). Modulation by cytokines of glucocorticoid action. Ann. N. Y. Acad. Sci..

[36] de Kloet E.R., Joels M., Holsboer F. (2005). Stress and the brain: From adaptation to disease. Nat. Rev. Neurosci..

[37] Hildebrandt H., Stachowiak R., Heber I., Schlake H.P., Eling P. (2020). Relation between cognitive fatigue and circadian or stress related cortisol levels in MS patients. Mult. Scler. Relat. Disord..

[38] Slominski R.M., Tuckey R.C., Manna P.R., Jetten A.M., Postlethwaite A., Raman C., Slominski A.T. (2020). Extra-adrenal glucocorticoid biosynthesis: Implications for autoimmune and inflammatory disorders. Genes. Immun..

[39] Tucha L., Fuermaier A.B., Koerts J., Buggenthin R., Aschenbrenner S., Weisbrod M., Thome J., Lange K.W., Tucha O. (2017). Sustained attention in adult ADHD: Time-on-task effects of various measures of attention. J. Neural Transm..

[40] Vedhara K., Hyde J., Gilchrist I.D., Tytherleigh M., Plummer S. (2000). Acute stress, memory, attention and cortisol. Psychoneuroendocrinology.

[41] Pereira G.M., Soares N.M., Souza A.R., Becker J., Finkelsztejn A., Almeida R.M.M. (2018). Basal cortisol levels and the relationship with clinical symptoms in multiple sclerosis: A systematic review. Arq. Neuropsiquiatr..

[42] Heidbrink C., Hausler S.F., Buttmann M., Ossadnik M., Strik H.M., Keller A., Buck D., Verbraak E., van Meurs M., Krockenberger M. (2010). Reduced cortisol levels in cerebrospinal fluid and differential distribution of 11beta-hydroxysteroid dehydrogenases in multiple sclerosis: Implications for lesion pathogenesis. Brain Behav. Immun..

[43] Foroughipour A., Norbakhsh V., Najafabadi S.H., Meamar R. (2012). Evaluating sex hormone levels in reproductive age women with multiple sclerosis and their relationship with disease severity. J. Res. Med. Sci..

[44] Wei T., Lightman S.L. (1997). The neuroendocrine axis in patients with multiple sclerosis. Brain.

[45] Hamidovic A., Karapetyan K., Serdarevic F., Choi S.H., Eisenlohr-Moul T., Pinna G. (2020). Higher Circulating Cortisol in the Follicular vs. Luteal Phase of the Menstrual Cycle: A Meta-Analysis. Front. Endocrinol..

[46] Marx C.E., Keefe R.S., Buchanan R.W., Hamer R.M., Kilts J.D., Bradford D.W., Strauss J.L., Naylor J.C., Payne V.M., Lieberman J.A. (2009). Proof-of-concept trial with the neurosteroid pregnenolone targeting cognitive and negative symptoms in schizophrenia. Neuropsychopharmacology.

[47] Matuszewska A., Kowalski K., Jawien P., Tomkalski T., Gawel-Dabrowska D., Merwid-Lad A., Szelag E., Blaszczak K., Wiatrak B., Danielewski M. (2023). The Hypothalamic-Pituitary-Gonadal Axis in Men with Schizophrenia. Int. J. Mol. Sci..

[48] Tomassini V., Pozzilli C. (2009). Sex hormones, brain damage and clinical course of Multiple Sclerosis. J. Neurol. Sci..

[49] Gubbels Bupp M.R., Jorgensen T.N. (2018). Androgen-Induced Immunosuppression. Front. Immunol..

[50] Tomassini V., Onesti E., Mainero C., Giugni E., Paolillo A., Salvetti M., Nicoletti F., Pozzilli C. (2005). Sex hormones modulate brain damage in multiple sclerosis: MRI evidence. J. Neurol. Neurosurg. Psychiatry.

[51] Garcia-Estrada J., Del Rio J.A., Luquin S., Soriano E., Garcia-Segura L.M. (1993). Gonadal hormones down-regulate reactive gliosis and astrocyte proliferation after a penetrating brain injury. Brain Res..

[52] Bovolenta P., Wandosell F., Nieto-Sampedro M. (1992). CNS glial scar tissue: A source of molecules which inhibit central neurite outgrowth. Prog. Brain Res..

[53] Burda J.E., Sofroniew M.V. (2014). Reactive gliosis and the multicellular response to CNS damage and disease. Neuron.

[54] Dalal M., Kim S., Voskuhl R.R. (1997). Testosterone therapy ameliorates experimental autoimmune encephalomyelitis and induces a T helper 2 bias in the autoantigen-specific T lymphocyte response. J. Immunol..

[55] Caruso A., Di Giorgi Gerevini V., Castiglione M., Marinelli F., Tomassini V., Pozzilli C., Caricasole A., Bruno V., Caciagli F., Moretti A. (2004). Testosterone amplifies excitotoxic damage of cultured oligodendrocytes. J. Neurochem..

[56] Ghoumari A.M., Ibanez C., El-Etr M., Leclerc P., Eychenne B., O’Malley B.W., Baulieu E.E., Schumacher M. (2003). Progesterone and its metabolites increase myelin basic protein expression in organotypic slice cultures of rat cerebellum. J. Neurochem..

[57] Bodhankar S., Wang C., Vandenbark A.A., Offner H. (2011). Estrogen-induced protection against experimental autoimmune encephalomyelitis is abrogated in the absence of B cells. Eur. J. Immunol..

[58] Gupta M.K., Guryev O.L., Auchus R.J. (2003). 5alpha-reduced C21 steroids are substrates for human cytochrome P450c17. Arch. Biochem. Biophys..

[59] Rege J., Nakamura Y., Wang T., Merchen T.D., Sasano H., Rainey W.E. (2014). Transcriptome profiling reveals differentially expressed transcripts between the human adrenal zona fasciculata and zona reticularis. J. Clin. Endocrinol. Metab..

[60] Park-Chung M., Wu F.S., Purdy R.H., Malayev A.A., Gibbs T.T., Farb D.H. (1997). Distinct sites for inverse modulation of N-methyl-D-aspartate receptors by sulfated steroids. Mol. Pharmacol..

[61] Park-Chung M., Malayev A., Purdy R.H., Gibbs T.T., Farb D.H. (1999). Sulfated and unsulfated steroids modulate gamma-aminobutyric acidA receptor function through distinct sites. Brain Res..

[62] Smejkalova T., Korinek M., Krusek J., Hrcka Krausova B., Candelas Serra M., Hajdukovic D., Kudova E., Chodounska H., Vyklicky L. (2021). Endogenous neurosteroids pregnanolone and pregnanolone sulfate potentiate presynaptic glutamate release through distinct mechanisms. Br. J. Pharmacol..

[63] Labrie F. (2004). Adrenal androgens and intracrinology. Semin. Reprod. Med..

[64] Majewska M.D. (1990). Steroid regulation of the GABAA receptor: Ligand binding, chloride transport and behaviour. Ciba Found. Symp..

[65] Petrovic M., Sedlacek M., Horak M., Chodounska H., Vyklicky L. (2005). 20-oxo-5beta-pregnan-3alpha-yl sulfate is a use-dependent NMDA receptor inhibitor. J. Neurosci..

[66] Johansson T., Le Greves P. (2005). The effect of dehydroepiandrosterone sulfate and allopregnanolone sulfate on the binding of [(3)H]ifenprodil to the N-methyl-d-aspartate receptor in rat frontal cortex membrane. J. Steroid Biochem. Mol. Biol..

[67] Barnard L., Gent R., van Rooyen D., Swart A.C. (2017). Adrenal C11-oxy C(21) steroids contribute to the C11-oxy C(19) steroid pool via the backdoor pathway in the biosynthesis and metabolism of 21-deoxycortisol and 21-deoxycortisone. J. Steroid Biochem. Mol. Biol..

[68] do Nascimento F.V., Piccoli V., Beer M.A., von Frankenberg A.D., Crispim D., Gerchman F. (2015). Association of HSD11B1 polymorphic variants and adipose tissue gene expression with metabolic syndrome, obesity and type 2 diabetes mellitus: A systematic review. Diabetol. Metab. Syndr..

[69] Bottasso O., Bay M.L., Besedovsky H., del Rey A. (2007). The immuno-endocrine component in the pathogenesis of tuberculosis. Scand. J. Immunol..

[70] Du C., Khalil M.W., Sriram S. (2001). Administration of dehydroepiandrosterone suppresses experimental allergic encephalomyelitis in SJL/J mice. J. Immunol..

[71] Rontzsch A., Thoss K., Petrow P.K., Henzgen S., Brauer R. (2004). Amelioration of murine antigen-induced arthritis by dehydroepiandrosterone (DHEA). Inflamm. Res..

[72] Tan X.D., Dou Y.C., Shi C.W., Duan R.S., Sun R.P. (2009). Administration of dehydroepiandrosterone ameliorates experimental autoimmune neuritis in Lewis rats. J. Neuroimmunol..

[73] Choi I.S., Cui Y., Koh Y.A., Lee H.C., Cho Y.B., Won Y.H. (2008). Effects of dehydroepiandrosterone on Th2 cytokine production in peripheral blood mononuclear cells from asthmatics. Korean J. Intern. Med..

[74] Sudo N., Yu X.N., Kubo C. (2001). Dehydroepiandrosterone attenuates the spontaneous elevation of serum IgE level in NC/Nga mice. Immunol. Lett..

[75] Kasperska-Zajac A., Brzoza Z., Rogala B. (2008). Dehydroepiandrosterone and dehydroepiandrosterone sulphate in atopic allergy and chronic urticaria. Inflammation.

[76] Romagnani S., Kapsenberg M., Radbruch A., Adorini L. (1998). Th1 and Th2 cells. Res. Immunol..

[77] Pratschke S., von Dossow-Hanfstingl V., Dietz J., Schneider C.P., Tufman A., Albertsmeier M., Winter H., Angele M.K. (2014). Dehydroepiandrosterone modulates T-cell response after major abdominal surgery. J. Surg. Res..

[78] Sterzl I., Hampl R., Sterzl J., Votruba J., Starka L. (1999). 7Beta-OH-DHEA counteracts dexamethasone induced suppression of primary immune response in murine spleenocytes. J. Steroid Biochem. Mol. Biol..

[79] Hennebert O., Chalbot S., Alran S., Morfin R. (2007). Dehydroepiandrosterone 7alpha-hydroxylation in human tissues: Possible interference with type 1 11beta-hydroxysteroid dehydrogenase-mediated processes. J. Steroid Biochem. Mol. Biol..

[80] Le Mee S., Hennebert O., Ferrec C., Wulfert E., Morfin R. (2008). 7beta-Hydroxy-epiandrosterone-mediated regulation of the prostaglandin synthesis pathway in human peripheral blood monocytes. Steroids.

[81] Pettersson H., Lundqvist J., Norlin M. (2010). Effects of CYP7B1-mediated catalysis on estrogen receptor activation. Biochim. Biophys. Acta.

[82] Tang W., Eggertsen G., Chiang J.Y., Norlin M. (2006). Estrogen-mediated regulation of CYP7B1: A possible role for controlling DHEA levels in human tissues. J. Steroid Biochem. Mol. Biol..

[83] Ahlem C.N., Page T.M., Auci D.L., Kennedy M.R., Mangano K., Nicoletti F., Ge Y., Huang Y., White S.K., Villegas S. (2011). Novel components of the human metabolome: The identification, characterization and anti-inflammatory activity of two 5-androstene tetrols. Steroids.

[84] Reading C.L., Frincke J.M., White S.K. (2012). Molecular targets for 17alpha-ethynyl-5-androstene-3beta,7beta,17beta-triol, an anti-inflammatory agent derived from the human metabolome. PLoS ONE.

[85] Reddy D.S. (2010). Neurosteroids: Endogenous role in the human brain and therapeutic potentials. Prog. Brain Res..

[86] Balan I., Beattie M.C., O’Buckley T.K., Aurelian L., Morrow A.L. (2019). Endogenous Neurosteroid (3alpha,5alpha)3-Hydroxypregnan-20-one Inhibits Toll-like-4 Receptor Activation and Pro-inflammatory Signaling in Macrophages and Brain. Sci. Rep..

[87] Lapchak P.A. (2004). The neuroactive steroid 3-alpha-ol-5-beta-pregnan-20-one hemisuccinate, a selective NMDA receptor antagonist improves behavioral performance following spinal cord ischemia. Brain Res..

[88] Kudova E., Mares P., Hill M., Vondrakova K., Tsenov G., Chodounska H., Kubova H., Vales K. (2021). The Neuroactive Steroid Pregnanolone Glutamate: Anticonvulsant Effect, Metabolites and Its Effect on Neurosteroid Levels in Developing Rat Brains. Pharmaceuticals.

[89] Abramova V., Leal Alvarado V., Hill M., Smejkalova T., Maly M., Vales K., Dittert I., Bozikova P., Kysilov B., Hrcka Krausova B. (2023). Effects of Pregnanolone Glutamate and Its Metabolites on GABA(A) and NMDA Receptors and Zebrafish Behavior. ACS Chem. Neurosci..

[90] Munawar Cheema M., Macakova Kotrbova Z., Hrcka Krausova B., Adla S.K., Slavikova B., Chodounska H., Kratochvil M., Vondrasek J., Sedlak D., Balastik M. (2024). 5beta-reduced neuroactive steroids as modulators of growth and viability of postnatal neurons and glia. J. Steroid Biochem. Mol. Biol..

[91] Akwa Y. (2020). Steroids and Alzheimer’s Disease: Changes Associated with Pathology and Therapeutic Potential. Int. J. Mol. Sci..

[92] Hill M., Parizek A., Simjak P., Koucky M., Anderlova K., Krejci H., Vejrazkova D., Ondrejikova L., Cerny A., Kancheva R. (2021). Steroids, steroid associated substances and gestational diabetes mellitus. Physiol. Res..

[93] Burczynski M.E., Sridhar G.R., Palackal N.T., Penning T.M. (2001). The reactive oxygen species--and Michael acceptor-inducible human aldo-keto reductase AKR1C1 reduces the alpha,beta-unsaturated aldehyde 4-hydroxy-2-nonenal to 1,4-dihydroxy-2-nonene. J. Biol. Chem..

[94] Murgia F., Giagnoni F., Lorefice L., Caria P., Dettori T., D’Alterio M.N., Angioni S., Hendren A.J., Caboni P., Pibiri M. (2022). Sex Hormones as Key Modulators of the Immune Response in Multiple Sclerosis: A Review. Biomedicines.

[95] Xu C., Liu W., You X., Leimert K., Popowycz K., Fang X., Wood S.L., Slater D.M., Sun Q., Gu H. (2015). PGF2alpha modulates the output of chemokines and pro-inflammatory cytokines in myometrial cells from term pregnant women through divergent signaling pathways. Mol. Hum. Reprod..

[96] Zheng L., Fei J., Feng C.M., Xu Z., Fu L., Zhao H. (2021). Serum 8-iso-PGF2alpha Predicts the Severity and Prognosis in Patients With Community-Acquired Pneumonia: A Retrospective Cohort Study. Front. Med..

[97] Sharma I., Dhaliwal L.K., Saha S.C., Sangwan S., Dhawan V. (2010). Role of 8-iso-prostaglandin F2alpha and 25-hydroxycholesterol in the pathophysiology of endometriosis. Fertil. Steril..

[98] Tchernof A., Mansour M.F., Pelletier M., Boulet M.M., Nadeau M., Luu-The V. (2015). Updated survey of the steroid-converting enzymes in human adipose tissues. J. Steroid Biochem. Mol. Biol..

[99] Nakamura Y., Hornsby P.J., Casson P., Morimoto R., Satoh F., Xing Y., Kennedy M.R., Sasano H., Rainey W.E. (2009). Type 5 17beta-hydroxysteroid dehydrogenase (AKR1C3) contributes to testosterone production in the adrenal reticularis. J. Clin. Endocrinol. Metab..

[100] Ostinelli G., Vijay J., Vohl M.C., Grundberg E., Tchernof A. (2021). AKR1C2 and AKR1C3 expression in adipose tissue: Association with body fat distribution and regulatory variants. Mol. Cell Endocrinol..

[101] Rizner T.L., Penning T.M. (2014). Role of aldo-keto reductase family 1 (AKR1) enzymes in human steroid metabolism. Steroids.

[102] Luu-The V. (2013). Assessment of steroidogenesis and steroidogenic enzyme functions. J. Steroid Biochem. Mol. Biol..

[103] Miller W.L., Auchus R.J. (2011). The molecular biology, biochemistry, and physiology of human steroidogenesis and its disorders. Endocr. Rev..

[104] Thompson A.J., Banwell B.L., Barkhof F., Carroll W.M., Coetzee T., Comi G., Correale J., Fazekas F., Filippi M., Freedman M.S. (2018). Diagnosis of multiple sclerosis: 2017 revisions of the McDonald criteria. Lancet Neurol..

[105] Hill M., Hana V., Velikova M., Parizek A., Kolatorova L., Vitku J., Skodova T., Simkova M., Simjak P., Kancheva R. (2019). A method for determination of one hundred endogenous steroids in human serum by gas chromatography-tandem mass spectrometry. Physiol. Res..

