# Supplementary material for: Effect of Treatment on Steroidome in Women with Multiple Sclerosis
Source: Int J Mol Sci. 2025 Feb 20;26(5):1835. doi: 10.3390/ijms26051835 (PMC11899614; doi:10.3390/ijms26051835)
Supplement: Supplementary file 1 [file ijms-26-01835-s001.zip › Effect of treatment on steroidome in women with multiple sclerosis v021, Table S1.pdf]

Table S1. Changes of steroids, steroid molar ratios that may reflect activities of steroidogenic enzymes and balances between them, and of indices of MS severity after treatment with anti-MS drugs shown as re-transformed means with their 95% confidence intervals.

	Follicular phase				Luteal phase			
Variable	n	Before treatment	n	After treatment	n	Before treatment	n	After treatment
**Δ^5 Pregnanes**								
Pregnenolone (Preg) [nM]	44	2.26 (1.95, 2.62)	43	2.77 (2.38, 3.24)	14	2.54 (1.83, 3.55)	17	3.77 (2.79, 5.12)
Stage: F=5.1, p=0.028, η_p^2=0.0848; PMC: F=0.8, p=0.376, η_p^2=0.0143; Stage × PMC: F=0.4, p=0.543, η_p^2=0.00675; Subj: F=2.3, p=0.001, η_p^2=0.709								
Pregnenolone sulfate (PregC) [nM]	43	136 (122, 152)	41	138 (123, 154)	13	84.9 (66.1, 108)	17	102 (82.1, 126)
Stage: F=1, p=0.331, η_p^2=0.0182; PMC: F=5.3, p=0.026, η_p^2=0.0923; Stage × PMC: F=0.6, p=0.453, η_p^2=0.0109; Subj: F=3.7, p<0.001, η_p^2=0.805, F0>L0								
17-Hydroxypregnenolone (17-OH-Preg) [nM]	43	5.53 (4.66, 6.58)	42	5.57 (4.66, 6.67)	14	3.59 (2.47, 5.22)	17	5.47 (3.91, 7.69)
Stage: F=2, p=0.16, η_p^2=0.0363; PMC: F=0.7, p=0.403, η_p^2=0.013; Stage × PMC: F=1.5, p=0.227, η_p^2=0.0269; Subj: F=2.8, p<0.001, η_p^2=0.75								
17-Hydroxypregnenolone sulfate (17-OH-PregC) [nM]	43	11.2 (9.59, 13)	41	8.28 (7.02, 9.73)	13	4.83 (3.27, 6.95)	17	7.59 (5.54, 10.3)
Stage: F=0.2, p=0.64, η_p^2=0.00424; PMC: F=3.5, p=0.066, η_p^2=0.0635; Stage × PMC: F=5.4, p=0.025, η_p^2=0.0933; Subj: F=3, p<0.001, η_p^2=0.768, F0>L0								
16α-Hydroxypregnenolone (16α-OH-Preg) [nM]	43	0.446 (0.384, 0.515)	43	0.362 (0.309, 0.422)	14	0.275 (0.19, 0.389)	16	0.355 (0.257, 0.482)
Stage: F=0, p=0.899, η_p^2=0.000303; PMC: F=1, p=0.317, η_p^2=0.0185; Stage × PMC: F=2.4, p=0.131, η_p^2=0.0417; Subj: F=3.2, p<0.001, η_p^2=0.777								
20α-Dihydropregnenolone (20α-DHPreg) [nM]	42	3.59 (3.34, 3.86)	42	3.3 (3.07, 3.55)	13	3.84 (3.27, 4.5)	15	3.74 (3.22, 4.33)
Stage: F=0.8, p=0.39, η_p^2=0.0142; PMC: F=0.7, p=0.409, η_p^2=0.0131; Stage × PMC: F=0.2, p=0.699, η_p^2=0.0029; Subj: F=5.4, p<0.001, η_p^2=0.854								
20α-Dihydropregnenolone sulfate (20α-DHPregC) [nM]	40	791 (721, 865)	40	736 (668, 807)	13	613 (489, 755)	15	590 (475, 720)
Stage: F=0.5, p=0.503, η_p^2=0.00922; PMC: F=2.4, p=0.128, η_p^2=0.0467; Stage × PMC: F=0, p=0.839, η_p^2=0.000849; Subj: F=3.8, p<0.001, ηp2=0.808								
**Δ^5 Androstanes**								
Dehydroepiandrosterone (DHEA) [nM]	44	8.11 (7.14, 9.21)	43	9.84 (8.63, 11.2)	14	6.6 (4.93, 8.77)	17	10.1 (7.83, 12.9)
Stage: F=7.5, p=0.009, η_p^2=0.119; PMC: F=0.2, p=0.656, η_p^2=0.00364; Stage × PMC: F=0.8, p=0.374, η_p^2=0.0144; Subj: F=2.8, p<0.001, η_p^2=0.751								
DHEA sulfate (DHEAC) [µM]	42	2.75 (2.5, 3.01)	40	2.56 (2.31, 2.83)	13	1.51 (1.17, 1.92)	17	1.91 (1.56, 2.33)
Stage: F=0.7, p=0.417, η_p^2=0.013; PMC: F=8.1, p=0.006, η_p^2=0.137; Stage × PMC: F=2.1, p=0.155, ηp2=0.0392; Subj: F=4.5, p<0.001, η_p^2=0.835, F0>L0								
7α-Hydroxy-DHEA (7α-OH-DHEA) [nM]	43	1.03 (0.917, 1.17)	43	0.799 (0.702, 0.906)	14	0.77 (0.577, 1.01)	16	0.671 (0.509, 0.869)
Stage: F=3.3, p=0.074, η_p^2=0.0578; PMC: F=1.4, p=0.249, η_p^2=0.0245; Stage × PMC: F=0.3, p=0.602, η_p^2=0.00506; Subj: F=4, p<0.001, η_p^2=0.809, F0>F1								
7β-Hydroxy-DHEA (7β-OH-DHEA) [nM]	42	0.482 (0.431, 0.538)	42	0.432 (0.384, 0.485)	14	0.279 (0.207, 0.366)	16	0.329 (0.253, 0.419)
Stage: F=0, p=0.851, η_p^2=0.000673; PMC: F=4.6, p=0.036, η_p^2=0.0801; Stage × PMC: F=1.3, p=0.252, η_p^2=0.0247; Subj: F=4.7, p<0.001, η_p^2=0.835, F0>L0								
Androstenediol (Adiol) [nM]	42	1.87 (1.73, 2.02)	42	1.9 (1.75, 2.05)	14	1.64 (1.38, 1.94)	16	1.96 (1.68, 2.28)
Stage: F=2.1, p=0.157, η_p^2=0.0374; PMC: F=0.2, p=0.692, η_p^2=0.00298; Stage × PMC: F=1.1, p=0.291, η_p^2=0.021; Subj: F=5.9, p<0.001, η_p^2=0.863								
Androstenediol sulfate (AdiolC) [µM]	44	0.883 (0.77, 1.01)	42	0.781 (0.671, 0.902)	13	0.439 (0.281, 0.638)	17	0.667 (0.485, 0.884)
Stage: F=0.8, p=0.365, η_p^2=0.0155; PMC: F=3.2, p=0.08, η_p^2=0.0566; Stage × PMC: F=2.8, p=0.101, η_p^2=0.0501; Subj: F=2.2, p=0.002, η_p^2=0.712, F0>L0								
5-Androstene-3β,7α,17β-triol (3β,7α,17β-AT) [nM]	43	0.328 (0.286, 0.378)	43	0.289 (0.252, 0.333)	14	0.298 (0.22, 0.405)	16	0.286 (0.217, 0.38)
Stage: F=0.5, p=0.495, η_p^2=0.00865; PMC: F=0.1, p=0.808, η_p^2=0.0011; Stage × PMC: F=0.1, p=0.749, η_p^2=0.00191; Subj: F=4.4, p<0.001, η_p^2=0.825								
5-Androstene-3β,7β,17β-triol (3β,7β,17β-AT) [nM]	42	0.273 (0.238, 0.312)	42	0.187 (0.163, 0.215)	14	0.177 (0.13, 0.239)	16	0.21 (0.159, 0.276)
Stage: F=0.8, p=0.377, η_p^2=0.0148; PMC: F=0.5, p=0.468, η_p^2=0.00998; Stage × PMC: F=4.2, p=0.045, η_p^2=0.0736; Subj: F=3.6, p<0.001, η_p^2=0.794, F0>F1								
5-Androstene-3β,16α,17β-triol (3β,16α,17β-AT) [pM]	44	159 (133, 191)	43	190 (157, 229)	14	137 (92.8, 204)	17	191 (134, 277)
Stage: F=2.6, p=0.115, η_p^2=0.0446; PMC: F=0.1, p=0.801, η_p^2=0.00116; Stage × PMC: F=0.2, p=0.659, η_p^2=0.00358; Subj: F=3, p<0.001, η_p^2=0.76								
5-Androstene-3β,16α,17β-triol sulfate (3β,16α,17β-ATC) [nM]	43	68 (58.7, 78.7)	41	66.5 (56.9, 77.6)	13	30.2 (20.8, 42.9)	17	41.7 (30.7, 56.2)
Stage: F=1.1, p=0.295, ηp2=0.0211; PMC: F=7.3, p=0.01, ηp2=0.122; Stage × PMC: F=1.2, p=0.285, ηp2=0.022; Subj: F=3.4, p<0.001, ηp2=0.789, F0>L0								

Table S1, continued. Changes of steroids, steroid molar ratios that may reflect activities of steroidogenic enzymes and balances between them, and of indices of MS severity after treatment with anti-MS drugs shown as re-transformed means with their 95% confidence intervals

		**Follicular phase**				**Luteal phase**		
Variable	n	Before treatment	n	After treatment	n	Before treatment	n	After treatment
**Δ^4 Pregnanes**								
Progesterone (P) [nM]	44	0.192 (0.152, 0.244)	43	0.213 (0.166, 0.273)	14	7.77 (2.98, 26.6)	17	5.25 (2.36, 13.9)
Stage: F=0.1, p=0.808, η_p^2=0.00108; PMC: F=48.2, p<0.001, η_p^2=0.467; Stage × PMC: F=0.4, p=0.527, η_p^2=0.00732; Subj: F=1.1, p=0.371, η_p^2=0.54, F0<L0								
17-Hydroxyprogesterone (17-OH-P) [nM]	44	1.11 (0.946, 1.3)	43	0.829 (0.701, 0.978)	14	2.76 (1.95, 3.91)	17	3.22 (2.36, 4.39)
Stage: F=0.2, p=0.635, ηp2=0.00413; PMC: F=20.9, p<0.001, ηp2=0.275; Stage × PMC: F=2, p=0.167, ηp2=0.0345; Subj: F=2.1, p=0.003, ηp2=0.695, F0<L0								
17,20α-Dihydroxy-4-pregnene-3-one (17-OH-20α-DHP) [nM]	41	0.683 (0.614, 0.758)	41	0.604 (0.54, 0.673)	13	1.28 (1.03, 1.58)	15	1.22 (0.995, 1.48)
Stage: F=0.9, p=0.355, η_p^2=0.0168; PMC: F=17.1, p<0.001, η_p^2=0.251; Stage × PMC: F=0.1, p=0.753, η_p^2=0.00196; Subj: F=3.3, p<0.001, η_p^2=0.78, F0<L0								
16α-Hydroxyprogesterone (16α-OH-P) [nM]	44	0.323 (0.267, 0.39)	43	0.259 (0.212, 0.316)	14	0.903 (0.597, 1.37)	17	0.901 (0.623, 1.3)
Stage: F=0.4, p=0.513, η_p^2=0.00782; PMC: F=14.6, p<0.001, η_p^2=0.21; Stage × PMC: F=0.3, p=0.571, η_p^2=0.00586; Subj: F=2.5, p<0.001, η_p^2=0.728, F0<L0								
20α-Dihydroprogesterone (20α-DHP) [nM]	44	0.311 (0.263, 0.367)	43	0.305 (0.258, 0.363)	14	3.59 (2.05, 6.87)	17	2.78 (1.73, 4.75)
Stage: F=0.4, p=0.542, η_p^2=0.00681; PMC: F=52.9, p<0.001, η_p^2=0.49; Stage × PMC: F=0.2, p=0.658, η_p^2=0.00359; Subj: F=1.8, p=0.013, η_p^2=0.661, F0<L0								
Conjugated 20α-dihydroprogesterone (20α-DHPC) [nM]	44	1.11 (0.917, 1.34)	42	0.658 (0.536, 0.805)	13	1.25 (0.809, 1.92)	17	1.5 (1.02, 2.19)
Stage: F=0.9, p=0.337, η_p^2=0.0174; PMC: F=2.4, p=0.126, η_p^2=0.0436; Stage × PMC: F=3.2, p=0.081, η_p^2=0.0565; Subj: F=1.6, p=0.038, η_p^2=0.644, F0>F1								
**Δ^4 Androstanes**								
Androstenedione (A) [nM]	44	2.87 (2.56, 3.2)	43	2.56 (2.26, 2.88)	14	2.66 (2.02, 3.4)	17	4 (3.27, 4.84)
Stage: F=2.6, p=0.116, η_p^2=0.0443; PMC: F=1.2, p=0.275, η_p^2=0.0216; Stage × PMC: F=5.8, p=0.019, η_p^2=0.0955; Subj: F=2.5, p<0.001, η_p^2=0.728								
Testosterone (T) [nM]	43	0.544 (0.479, 0.615)	43	0.613 (0.54, 0.692)	14	0.44 (0.326, 0.582)	16	0.766 (0.601, 0.967)
Stage: F=9.8, p=0.003, η_p^2=0.154; PMC: F=0, p=0.945, η_p^2=0.0000883; Stage × PMC: F=3.3, p=0.077, η_p^2=0.0568; Subj: F=3, p<0.001, η_p^2=0.765, L0<L1, F1<L1								
**Estrogens**								
Estrone sulfate (E1C) [nM]	43	1.67 (1.37, 2.02)	42	1.25 (1.01, 1.53)	14	3.3 (2.2, 4.87)	17	2.15 (1.47, 3.08)
Stage: F=4.7, p=0.035, η_p^2=0.0798; PMC: F=4.2, p=0.045, η_p^2=0.0726; Stage × PMC: F=0.2, p=0.663, η_p^2=0.00355; Subj: F=2.1, p=0.004, η_p^2=0.688, F0<L0								
Estradiol (E2) [pM]	32	145 (118, 177)	33	133 (108, 162)	8	293 (179, 474)	8	267 (162, 431)
Stage: F=0.2, p=0.662, η_p^2=0.00522; PMC: F=4.2, p=0.049, η_p^2=0.101; Stage × PMC: F=0, p=0.974, η_p^2=0.00003; Subj: F=3.5, p<0.001, η_p^2=0.791, F0<L0								
**20-oxo-5α/β-Pregnanes**								
5α-Dihydroprogesterone [nM]	39	0.14 (0.113, 0.174)	43	0.18 (0.146, 0.223)	14	1.16 (0.679, 2.09)	17	1.16 (0.714, 1.96)
Stage: F=0.5, p=0.495, η_p^2=0.00937; PMC: F=32.5, p<0.001, η_p^2=0.394; Stage × PMC: F=0.4, p=0.543, η_p^2=0.00744; Subj: F=1.3, p=0.157, η_p^2=0.609, F0<L0								
Allopregnanolone [nM]	42	0.179 (0.15, 0.215)	42	0.205 (0.171, 0.247)	14	1.41 (0.903, 2.27)	17	1.32 (0.888, 2.01)
Stage: F=0.1, p=0.802, η_p^2=0.00119; PMC: F=44.1, p<0.001, η_p^2=0.454; Stage × PMC: F=0.3, p=0.59, η_p^2=0.00552; Subj: F=2.1, p=0.004, η_p^2=0.693, F0<L0								
Allopregnanolone sulfate [nM]	44	4.3 (3.76, 4.93)	42	3.45 (3.01, 3.97)	13	11.9 (8.18, 18)	17	10.4 (7.56, 14.7)
Stage: F=1.8, p=0.19, η_p^2=0.0322; PMC: F=20.3, p<0.001, η_p^2=0.277; Stage × PMC: F=0.2, p=0.683, η_p^2=0.00317; Subj: F=2.4, p<0.001, η_p^2=0.731, F0<L0								
Isopregnanolone [nM]	41	0.219 (0.182, 0.264)	41	0.372 (0.31, 0.446)	14	0.568 (0.39, 0.823)	16	0.715 (0.506, 1.01)
Stage: F=6.1, p=0.017, η_p^2=0.105; PMC: F=8.2, p=0.006, η_p^2=0.137; Stage × PMC: F=0.7, p=0.413, η_p^2=0.0129; Subj: F=1.8, p=0.015, η_p^2=0.662, F0<F1, F0<L0								
Isopregnanolone sulfate [nM]	44	11.2 (9.97, 12.7)	42	12.9 (11.3, 14.6)	13	14.7 (11.1, 19.6)	17	17.6 (13.7, 22.9)
Stage: F=2, p=0.167, η_p^2=0.0358; PMC: F=2.2, p=0.145, η_p^2=0.0396; Stage × PMC: F=0, p=0.887, η_p^2=0.000387; Subj: F=2.5, p<0.001, η_p^2=0.737								
Pregnanolone [nM]	42	0.0726 (0.0566, 0.0932)	43	0.0973 (0.0756, 0.126)	14	0.63 (0.335, 1.26)	17	0.586 (0.334, 1.08)
Stage: F=0.3, p=0.591, η_p^2=0.00548; PMC: F=22.5, p<0.001, η_p^2=0.298; Stage × PMC: F=0.5, p=0.475, η_p^2=0.00968; Subj: F=1.1, p=0.32, η_p^2=0.558, F0<L0								
Conjugated pregnanolone [nM]	42	9.3 (7.88, 11)	43	11.3 (9.55, 13.3)	14	37.1 (25, 56.6)	17	35.4 (24.9, 51.6)
Stage: F=0.3, p=0.591, η_p^2=0.00548; PMC: F=22.5, p<0.001, η_p^2=0.298; Stage × PMC: F=0.5, p=0.475, η_p^2=0.00968; Subj: F=1.1, p=0.32, η_p^2=0.558, F0<L0								
Conjugated epipregnanolone [nM]	41	2.62 (2.38, 2.9)	39	2.31 (2.09, 2.57)	13	3.78 (3.02, 4.77)	17	3.66 (3.01, 4.48)
Stage: F=0.8, p=0.375, η_p^2=0.0157; PMC: F=7.1, p=0.011, η_p^2=0.124; Stage × PMC: F=0.2, p=0.638, η_p^2=0.00445; Subj: F=4.7, p<0.001, η_p^2=0.84, F0<L0								

Table S1, continued. Changes of steroids, steroid molar ratios that may reflect activities of steroidogenic enzymes and balances between them, and of indices of MS severity after treatment with anti-MS drugs shown as re-transformed means with their 95% confidence intervals

	Follicular phase				Luteal phase			
Variable	n	Before treatment	n	After treatment	n	Before treatment	n	After treatment
**20α-Hydroxy-5α/β-pregnanes**								
5α,20α-Tetrahydroprogesterone [nM]	43	0.183 (0.155, 0.217)	42	0.219 (0.184, 0.261)	14	1.2 (0.765, 1.98)	17	1.14 (0.763, 1.77)
Stage: F=0.2, p=0.64, η_p^2=0.00408; PMC: F=36.7, p<0.001, η_p^2=0.405; Stage × PMC: F=0.4, p=0.509, η_p^2=0.0081; Subj: F=2, p=0.005, η_p^2=0.684, F0<L0								
Conjugated 5α,20α-tetrahydroprogesterone [nM]	44	0.165 (0.134, 0.204)	42	0.12 (0.0966, 0.149)	13	0.815 (0.466, 1.5)	17	0.815 (0.498, 1.38)
Stage: F=0.7, p=0.405, η_p^2=0.0131; PMC: F=23.7, p<0.001, η_p^2=0.309; Stage × PMC: F=0.6, p=0.463, η_p^2=0.0102; Subj: F=1.8, p=0.019, η_p^2=0.662, F0<L0								
5α-Pregnane-3α,20α-diol [nM]	43	0.63 (0.552, 0.72)	42	0.81 (0.705, 0.932)	14	1.84 (1.35, 2.56)	17	3.34 (2.45, 4.63)
Stage: F=10.8, p=0.002, η_p^2=0.166; PMC: F=31.2, p<0.001, η_p^2=0.366; Stage × PMC: F=1, p=0.32, η_p^2=0.0183; Subj: F=2.8, p<0.001, η_p^2=0.75, F0<L0								
Conjugated 5α-pregnane-3α,20α-diol [nM]	44	27.9 (24.7, 31.5)	41	27.5 (24.2, 31.3)	12	117 (78.6, 183)	17	95.4 (69.7, 135)
Stage: F=0.5, p=0.483, η_p^2=0.00952; PMC: F=33.6, p<0.001, η_p^2=0.392; Stage × PMC: F=0.3, p=0.612, η_p^2=0.00499; Subj: F=3.4, p<0.001, η_p^2=0.789, F0<L0								
5α-Pregnane-3β,20α-diol [nM]	38	0.305 (0.208, 0.449)	42	0.182 (0.127, 0.261)	14	0.491 (0.225, 1.13)	17	0.358 (0.179, 0.74)
Stage: F=1.8, p=0.189, η_p^2=0.0356; PMC: F=1.1, p=0.302, η_p^2=0.0222; Stage × PMC: F=0.1, p=0.769, η_p^2=0.00181; Subj: F=1, p=0.441, η_p^2=0.562								
Conjugated 5α-pregnane-3β,20α-diol [nM]	44	122 (98.4, 153)	42	70.4 (56.3, 88.1)	13	155 (93.8, 261)	17	146 (94.6, 231)
Stage: F=2.4, p=0.131, η_p^2=0.0425; PMC: F=2, p=0.168, η_p^2=0.0356; Stage × PMC: F=1.3, p=0.267, η_p^2=0.0232; Subj: F=1.7, p=0.024, η_p^2=0.656, F0>F1								
Conjugated 5β,20α-tetrahydroprogesterone [nM]	44	0.297 (0.25, 0.352)	42	0.288 (0.241, 0.345)	13	1.06 (0.707, 1.62)	17	0.801 (0.565, 1.15)
Stage: F=0.9, p=0.347, η_p^2=0.0167; PMC: F=17.4, p<0.001, η_p^2=0.248; Stage × PMC: F=0.5, p=0.503, η_p^2=0.00853; Subj: F=2.1, p=0.003, η_p^2=0.703, F0<L0								
5β-Pregnane-3α,20α-diol [nM]	44	0.258 (0.207, 0.32)	43	0.349 (0.279, 0.435)	14	1.06 (0.662, 1.69)	17	1.39 (0.913, 2.12)
Stage: F=2.3, p=0.134, η_p^2=0.0404; PMC: F=17.2, p<0.001, η_p^2=0.239; Stage × PMC: F=0, p=0.956, η_p^2=0.0000565; Subj: F=1.7, p=0.02, η_p^2=0.65, F0<L0								
Conjugated 5β-pregnane-3α,20α-diol [nM]	44	28.1 (24.5, 32.4)	42	35.2 (30.3, 41.1)	13	71.3 (49.3, 107)	17	78.1 (56, 113)
Stage: F=1.3, p=0.253, η_p^2=0.0246; PMC: F=12.5, p<0.001, η_p^2=0.19; Stage × PMC: F=0.3, p=0.614, η_p^2=0.00483; Subj: F=3.2, p<0.001, η_p^2=0.782, F0<L0								
5β-Pregnane-3β,20α-diol [nM]	44	0.232 (0.196, 0.275)	43	0.215 (0.182, 0.256)	14	0.235 (0.164, 0.348)	17	0.356 (0.251, 0.524)
Stage: F=1.2, p=0.286, η_p^2=0.0207; PMC: F=0.9, p=0.352, η_p^2=0.0158; Stage × PMC: F=2, p=0.167, η_p^2=0.0344; Subj: F=2.2, p=0.002, η_p^2=0.702								
Conjugated 5β-pregnane-3β,20α-diol [nM]	43	15.8 (13.8, 18.1)	41	13.9 (12, 16)	13	27.9 (20.3, 39.2)	17	24.6 (18.7, 32.9)
Stage: F=1, p=0.313, η_p^2=0.0196; PMC: F=6.9, p=0.011, η_p^2=0.118; Stage × PMC: F=0, p=0.958, η_p^2=0.0000541; Subj: F=3.7, p<0.001, η_p^2=0.807, F0<L0								
**17-Hydroxy-20-oxo- a 20α-hydroxy-5α/β-pregnanes**								
17-Hydroxyallopregnanolone sulfate [nM]	44	1.42 (1.23, 1.63)	43	1.02 (0.878, 1.19)	14	2.24 (1.66, 3)	17	1.75 (1.33, 2.29)
Stage: F=5.2, p=0.026, η_p^2=0.087; PMC: F=5, p=0.029, η_p^2=0.0836; Stage × PMC: F=0.1, p=0.831, η_p^2=0.000837; Subj: F=3.5, p<0.001, η_p^2=0.791, F0>F1, F0<L0								
17-Hydroxypregnanolone [nM]	38	0.0527 (0.0398, 0.0694)	41	0.0411 (0.031, 0.0541)	14	0.2 (0.118, 0.336)	17	0.188 (0.117, 0.3)
Stage: F=0.5, p=0.496, η_p^2=0.00972; PMC: F=13.7, p<0.001, η_p^2=0.222; Stage × PMC: F=0.1, p=0.732, η_p^2=0.00246; Subj: F=1.7, p=0.027, η_p^2=0.676, F0<L0								
Conjugated 17-hydroxypregnanolone [nM]	44	8.3 (7.25, 9.47)	43	5.86 (5.07, 6.76)	14	11.9 (8.92, 15.8)	17	8.65 (6.62, 11.2)
Stage: F=8, p=0.007, η_p^2=0.127; PMC: F=3.2, p=0.082, η_p^2=0.0541; Stage × PMC: F=0, p=0.961, η_p^2=0.0000441; Subj: F=2.4, p<0.001, η_p^2=0.721, F0>F1								
5α-Pregnane-3α,17,20α-triol [pM]	42	105 (89.6, 122)	42	129 (111, 150)	13	180 (130, 246)	15	128 (93.4, 173)
Stage: F=0.3, p=0.608, η_p^2=0.00511; PMC: F=1.3, p=0.264, η_p^2=0.0239; Stage × PMC: F=3.4, p=0.073, η_p^2=0.0607; Subj: F=4.2, p<0.001, η_p^2=0.819, F0<L0								
Conjugated 5α-pregnane-3α,17,20α-triol [nM]	44	29.5 (22.4, 38.9)	43	10.2 (7.68, 13.4)	14	16.9 (9.33, 31)	17	11.7 (6.82, 19.9)
Stage: F=9.1, p=0.004, η_p^2=0.142; PMC: F=0.2, p=0.634, η_p^2=0.00415; Stage × PMC: F=1.6, p=0.205, η_p^2=0.029; Subj: F=1.9, p=0.011, η_p^2=0.666, F0>F1								
5β-Pregnane-3α,17,20α-triol [nM]	42	1.82 (1.65, 2.01)	42	1.98 (1.79, 2.18)	14	2.42 (1.97, 2.94)	16	2.38 (1.97, 2.85)
Stage: F=0.2, p=0.703, η_p^2=0.00277; PMC: F=2.4, p=0.128, η_p^2=0.0433; Stage × PMC: F=0.3, p=0.608, η_p^2=0.00501; Subj: F=5.3, p<0.001, η_p^2=0.851								
Conjugated 5β-pregnane-3α,17,20α-triol [nM]	44	126 (107, 148)	42	55.1 (44.9, 67.1)	13	87.3 (57, 130)	17	67.1 (45.9, 95.8)
Stage: F=11.7, p=0.001, η_p^2=0.178; PMC: F=0.1, p=0.742, η_p^2=0.00202; Stage × PMC: F=2.6, p=0.114, η_p^2=0.0455; Subj: F=3.4, p<0.001, η_p^2=0.787, F0>F1								

Table S1, continued. Changes of steroids, steroid molar ratios that may reflect activities of steroidogenic enzymes and balances between them, and of indices of MS severity after treatment with anti-MS drugs shown as re-transformed means with their 95% confidence intervals

Variable	Follicular phase n	Follicular phase Before treatment	n	After treatment	Luteal phase n	Luteal phase Before treatment	n	After treatment
**17-oxo-5α/β-Androstanes**								
5α-Androstane-3,17-dione [nM]	42	0.25 (0.213, 0.292)	42	0.245 (0.208, 0.287)	14	0.25 (0.176, 0.35)	16	0.305 (0.222, 0.415)
Stage: F=0.5, p=0.505, η_p^2=0.00845; PMC: F=0.2, p=0.659, η_p^2=0.00371; Stage × PMC: F=0.5, p=0.477, η_p^2=0.00958; Subj: F=1.7, p=0.029, η_p^2=0.644								
Androsterone [nM]	42	0.566 (0.516, 0.619)	42	0.522 (0.475, 0.574)	14	0.434 (0.35, 0.532)	16	0.575 (0.477, 0.688)
Stage: F=1.5, p=0.22, η_p^2=0.0282; PMC: F=0.3, p=0.574, η_p^2=0.00601; Stage × PMC: F=4, p=0.052, η_p^2=0.0696; Subj: F=4.4, p<0.001, η_p^2=0.825								
Androsterone sulfate [μM]	41	1.05 (0.913, 1.2)	40	1.18 (1.03, 1.35)	13	0.509 (0.357, 0.709)	15	0.651 (0.478, 0.872)
Stage: F=2, p=0.162, η_p^2=0.0388; PMC: F=8.6, p=0.005, η_p^2=0.147; Stage × PMC: F=0.1, p=0.717, η_p^2=0.00265; Subj: F=4.3, p<0.001, η_p^2=0.826, F0>L0								
Epiandrosterone [nM]	41	0.344 (0.31, 0.381)	41	0.37 (0.333, 0.41)	13	0.304 (0.238, 0.383)	15	0.397 (0.321, 0.486)
Stage: F=3.3, p=0.073, η_p^2=0.0615; PMC: F=0, p=0.881, η_p^2=0.000442; Stage × PMC: F=0.8, p=0.363, η_p^2=0.0162; Subj: F=6.2, p<0.001, η_p^2=0.87								
Epiandrosterone sulfate [nM]	40	303 (274, 335)	39	340 (308, 376)	13	197 (154, 248)	15	263 (213, 322)
Stage: F=4.9, p=0.031, η_p^2=0.0916; PMC: F=4.7, p=0.035, η_p^2=0.0873; Stage × PMC: F=0.6, p=0.431, η_p^2=0.0127; Subj: F=6.1, p<0.001, η_p^2=0.871, F0>L0								
Etiocholanolone [nM]	41	0.246 (0.224, 0.27)	41	0.25 (0.227, 0.274)	14	0.209 (0.168, 0.256)	16	0.245 (0.203, 0.294)
Stage: F=1.1, p=0.29, η_p^2=0.0215; PMC: F=0.4, p=0.553, η_p^2=0.00683; Stage × PMC: F=0.6, p=0.435, η_p^2=0.0118; Subj: F=4.7, p<0.001, η_p^2=0.835								
Etiocholanolone sulfate [nM]	42	78.5 (70.1, 87.7)	40	78.5 (69.7, 88.2)	13	52.3 (39, 68.5)	16	53 (40.9, 67.3)
Stage: F=0, p=0.955, η_p^2=0.0000634; PMC: F=4.7, p=0.035, η_p^2=0.0843; Stage × PMC: F=0, p=0.962, η_p^2=0.000046; Subj: F=3.9, p<0.001, η_p^2=0.81, F0>L0								
Epietiocholanolone sulfate [nM]	42	23.9 (20.1, 28.4)	41	21.3 (17.9, 25.5)	13	9.44 (6.22, 14)	15	10.7 (7.35, 15.4)
Stage: F=0, p=0.977, η_p^2=0.0000162; PMC: F=8.4, p=0.006, η_p^2=0.141; Stage × PMC: F=0.4, p=0.508, η_p^2=0.00864; Subj: F=3.5, p<0.001, η_p^2=0.793, F0>L0								
**17β-Hydroxy-5α/β-androstanes**								
5α-Dihydrotestosterone [pM]	43	159 (134, 189)	43	210 (176, 251)	14	104 (71.5, 152)	16	135 (95.6, 192)
Stage: F=3.3, p=0.077, η_p^2=0.0567; PMC: F=2.4, p=0.125, η_p^2=0.043; Stage × PMC: F=0, p=0.968, η_p^2=0.0000307; Subj: F=2.4, p<0.001, η_p^2=0.722								
Conjugated 5α-dihydrotestosterone [nM]	42	1.04 (0.836, 1.29)	42	0.887 (0.715, 1.1)	13	0.975 (0.612, 1.57)	16	0.5 (0.32, 0.774)
Stage: F=4.8, p=0.033, η_p^2=0.0863; PMC: F=0.9, p=0.361, η_p^2=0.0164; Stage × PMC: F=1.5, p=0.226, η_p^2=0.0286; Subj: F=1.4, p=0.1, η_p^2=0.618								
5α-Androstane-3α,17β-diol [pM]	42	91.6 (81.7, 103)	42	99.9 (89, 112)	14	84.8 (66.2, 109)	16	116 (92.1, 146)
Stage: F=4.1, p=0.049, η_p^2=0.0715; PMC: F=0, p=0.846, η_p^2=0.000715; Stage × PMC: F=1, p=0.316, η_p^2=0.019; Subj: F=4.9, p<0.001, η_p^2=0.84								
Conjugated 5α-androstane-3α,17β-diol [nM]	42	30.9 (27.1, 35.2)	41	26 (22.6, 29.7)	13	13 (9.4, 17.8)	16	14.7 (11, 19.4)
Stage: F=0.1, p=0.783, η_p^2=0.0015; PMC: F=11.1, p=0.002, η_p^2=0.178; Stage × PMC: F=1.1, p=0.292, η_p^2=0.0218; Subj: F=3.2, p<0.001, η_p^2=0.784, F0>L0								
Conjugated 5α-androstane-3β,17β-diol [nM]	44	53.2 (45.2, 62.5)	42	52.3 (44, 61.9)	13	25.8 (17.1, 38.1)	17	27.4 (19.2, 38.5)
Stage: F=0, p=0.9, η_p^2=0.000303; PMC: F=6.6, p=0.013, η_p^2=0.111; Stage × PMC: F=0.1, p=0.826, η_p^2=0.000917; Subj: F=2.6, p<0.001, η_p^2=0.742, F0>L0								
Conjugated 5β-androstane-3α,17β-diol [nM]	42	5.56 (4.84, 6.37)	41	4.41 (3.8, 5.1)	13	5.01 (3.65, 6.79)	16	4.05 (3.01, 5.39)
Stage: F=3.1, p=0.083, η_p^2=0.0579; PMC: F=0.2, p=0.677, η_p^2=0.00342; Stage × PMC: F=0, p=0.936, η_p^2=0.000129; Subj: F=3.1, p<0.001, η_p^2=0.774								
Conjugated 5β-androstane-3β,17β-diol [nM]	41	0.374 (0.306, 0.453)	41	0.258 (0.208, 0.319)	13	0.22 (0.133, 0.348)	15	0.231 (0.144, 0.355)
Stage: F=0.8, p=0.372, η_p^2=0.016; PMC: F=0.9, p=0.344, η_p^2=0.0179; Stage × PMC: F=1.1, p=0.309, η_p^2=0.0207; Subj: F=2.8, p<0.001, η_p^2=0.76								

Table S1, continued. Changes of steroids, steroid molar ratios that may reflect activities of steroidogenic enzymes and balances between them, and of indices of MS severity after treatment with anti-MS drugs shown as re-transformed means with their 95% confidence intervals

	Follicular phase				Luteal phase			
Variable	n	Before treatment	n	After treatment	n	Before treatment	n	After treatment
**Corticoids and 11β-hydroxyandrostanes**								
Cortisol [nM]	43	373 (336, 417)	42	289 (261, 320)	14	227 (187, 279)	17	226 (190, 272)
Stage: F=2.1, p=0.15, η_p^2=0.038; PMC: F=5.9, p=0.019, η_p^2=0.0985; Stage × PMC: F=1.6, p=0.217, η_p^2=0.0281; Subj: F=3.6, p<0.001, η_p^2=0.795, F0>F1, F0>L0								
Cortisol (RIA) [nM]	44	549 (497, 606)	42	436 (394, 483)	14	433 (348, 542)	16	352 (290, 428)
Stage: F=6.6, p=0.013, η_p^2=0.11; PMC: F=1.9, p=0.171, η_p^2=0.0351; Stage × PMC: F=0, p=0.925, η_p^2=0.000167; Subj: F=2.1, p=0.004, η_p^2=0.7, F0>F1								
Cortisone [nM]	42	122 (105, 144)	42	108 (93, 126)	14	92.2 (69.7, 127)	17	65.6 (52.6, 83.6)
Stage: F=4, p=0.051, η_p^2=0.0702; PMC: F=3.3, p=0.074, η_p^2=0.059; Stage × PMC: F=0.9, p=0.351, η_p^2=0.0164; Subj: F=1.5, p=0.07, η_p^2=0.621								
Corticosterone [nM]	43	13 (10.8, 15.5)	42	7.74 (6.35, 9.39)	14	6.46 (4.18, 9.77)	17	6.5 (4.41, 9.4)
Stage: F=2.5, p=0.12, η_p^2=0.0442; PMC: F=2.2, p=0.144, η_p^2=0.0392; Stage × PMC: F=2, p=0.16, η_p^2=0.0363; Subj: F=2.5, p<0.001, η_p^2=0.725, F0>F1, F0>L0								
11β-Hydroxyandrostenedione [nM]	44	51.5 (46, 57.6)	43	39.4 (34.9, 44.3)	13	29.9 (22.6, 39.2)	16	28.4 (22.1, 36.2)
Stage: F=2.3, p=0.137, η_p^2=0.0404; PMC: F=5.5, p=0.023, η_p^2=0.0917; Stage × PMC: F=0.9, p=0.357, η_p^2=0.0157; Subj: F=3.1, p<0.001, η_p^2=0.768, F0>F1, F0>L0								
11β-Hydroxyandrosterone [nM]	44	1.77 (1.56, 2.01)	43	1.45 (1.27, 1.66)	13	1.31 (0.961, 1.76)	16	0.986 (0.74, 1.3)
Stage: F=3.9, p=0.052, η_p^2=0.0679; PMC: F=2.7, p=0.107, η_p^2=0.0473; Stage × PMC: F=0.1, p=0.785, η_p^2=0.00139; Subj: F=4.8, p<0.001, η_p^2=0.837								
11β-Hydroxyandrosterone sulfate [nM]	42	30.3 (27.5, 33.2)	42	27.6 (24.9, 30.4)	13	15 (11.4, 19.3)	15	9.82 (7.3, 12.8)
Stage: F=5.7, p=0.02, η_p^2=0.0994; PMC: F=23.9, p<0.001, η_p^2=0.315; Stage × PMC: F=1.4, p=0.246, η_p^2=0.0258; Subj: F=5.8, p<0.001, η_p^2=0.862, F0>L0								
11β-Hydroxyepiandrosterone [nM]	42	96.3 (82.9, 111)	42	79.2 (67.3, 92.4)	13	93.7 (65.9, 129)	15	62.1 (42.7, 86.7)
Stage: F=4.7, p=0.036, η_p^2=0.0821; PMC: F=0.3, p=0.604, η_p^2=0.00522; Stage × PMC: F=0.4, p=0.523, η_p^2=0.00788; Subj: F=4.7, p<0.001, η_p^2=0.835								
11β-Hydroxyepiandrosterone sulfate [nM]	44	0.808 (0.695, 0.942)	43	0.815 (0.697, 0.955)	14	0.705 (0.506, 0.99)	17	0.789 (0.586, 1.07)
Stage: F=0.2, p=0.654, η_p^2=0.00368; PMC: F=0.1, p=0.728, η_p^2=0.00222; Stage × PMC: F=0.1, p=0.73, η_p^2=0.00218; Subj: F=2.9, p<0.001, η_p^2=0.754								
11β-Hydroxyetiocholanolone [nM]	42	1.68 (1.51, 1.85)	43	1.44 (1.29, 1.59)	13	1.45 (1.13, 1.83)	14	0.927 (0.702, 1.2)
Stage: F=8.9, p=0.004, η_p^2=0.147; PMC: F=2.5, p=0.119, η_p^2=0.046; Stage × PMC: F=1.5, p=0.232, η_p^2=0.0273; Subj: F=3.9, p<0.001, η_p^2=0.809								
11β-Hydroxyetiocholanolone sulfate [nM]	43	8.24 (7.05, 9.59)	42	9.16 (7.82, 10.7)	14	5.11 (3.41, 7.36)	17	2.97 (1.93, 4.35)
Stage: F=1.5, p=0.22, η_p^2=0.0277; PMC: F=9, p=0.004, η_p^2=0.142; Stage × PMC: F=3.2, p=0.078, η_p^2=0.0563; Subj: F=4.6, p<0.001, η_p^2=0.832								
**C17-hydroxylase, C17,20-lyase (CYP17A1), hydroxylase + lyase steps**								
DHEA/Preg	43	3.65 (3.31, 4.01)	42	3.4 (3.08, 3.75)	14	2.7 (2.17, 3.33)	17	2.69 (2.22, 3.25)
Stage: F=0.2, p=0.672, η_p^2=0.00335; PMC: F=3.2, p=0.082, η_p^2=0.0551; Stage × PMC: F=0.1, p=0.713, η_p^2=0.00253; Subj: F=2.5, p<0.001, η_p^2=0.729								
DHEA/Preg, C	43	19.7 (17.7, 21.8)	41	18.5 (16.5, 20.7)	13	17.9 (13.9, 22.6)	17	19.2 (15.5, 23.4)
Stage: F=0, p=0.97, η_p^2=0.0000274; PMC: F=0, p=0.862, η_p^2=0.000584; Stage × PMC: F=0.4, p=0.555, η_p^2=0.00675; Subj: F=2.5, p<0.001, η_p^2=0.737								
DHEA/20α-DHPreg	44	2.25 (2.02, 2.5)	43	2.82 (2.51, 3.17)	14	1.64 (1.31, 2.06)	17	2.17 (1.76, 2.7)
Stage: F=7.3, p=0.009, η_p^2=0.117; PMC: F=2.9, p=0.095, η_p^2=0.05; Stage × PMC: F=0.1, p=0.744, η_p^2=0.00196; Subj: F=2, p=0.006, η_p^2=0.68, F0<F1								
DHEA/20α-DHPreg, C	41	3.52 (3.21, 3.86)	40	3.72 (3.39, 4.08)	13	2.4 (1.92, 2.97)	16	2.9 (2.39, 3.49)
Stage: F=2.1, p=0.156, η_p^2=0.0398; PMC: F=4.6, p=0.037, η_p^2=0.0846; Stage × PMC: F=0.5, p=0.502, η_p^2=0.00907; Subj: F=2.8, p<0.001, η_p^2=0.756, F0>L0								
A/P	44	14.6 (11.5, 18.4)	43	11.1 (8.55, 14.2)	14	0.551 (-0.0442, 1.62)	17	1.24 (0.409, 2.61)
Stage: F=0.1, p=0.816, η_p^2=0.000992; PMC: F=28.7, p<0.001, η_p^2=0.343; Stage × PMC: F=1.9, p=0.18, η_p^2=0.0325; Subj: F=1, p=0.558, η_p^2=0.508, F0>L0								
A/20α-DHP	44	8.98 (7.62, 10.5)	43	7.69 (6.45, 9.11)	14	0.829 (0.317, 1.6)	17	1.67 (0.952, 2.68)
Stage: F=0.7, p=0.413, η_p^2=0.0122; PMC: F=33.7, p<0.001, η_p^2=0.38; Stage × PMC: F=2.8, p=0.099, η_p^2=0.0487; Subj: F=1.6, p=0.047, η_p^2=0.627, F0>L0								
5α-DHA/5α-DHP	39	1.76 (1.46, 2.13)	43	1.34 (1.12, 1.6)	14	0.26 (0.161, 0.402)	17	0.356 (0.239, 0.517)
Stage: F=0, p=0.993, η_p^2=0.0000014; PMC: F=32.3, p<0.001, η_p^2=0.392; Stage × PMC: F=2.4, p=0.125, η_p^2=0.0465; Subj: F=1.9, p=0.01, η_p^2=0.694, F0>L0								

Table S1, continued. Changes of steroids, steroid molar ratios that may reflect activities of steroidogenic enzymes and balances between them, and of indices of MS severity after treatment with anti-MS drugs shown as re-transformed means with their 95% confidence intervals

	Follicular phase				Luteal phase			
Variable	n	Before treatment	n	After treatment	n	Before treatment	n	After treatment
**C17-hydroxylase, C17,20-lyase (CYP17A1), hydroxylase + lyase steps**								

5α-DHA/5α,20α-THP	43	1.34 (1.15, 1.55)	42	0.995 (0.844, 1.17)	14	0.255 (0.146, 0.405)	17	0.382 (0.252, 0.553)
Stage: F=0, p=0.975, η_p^2=0.0000182; PMC: F=22.3, p<0.001, η_p^2=0.292; Stage × PMC: F=3.8, p=0.058, η_p^2=0.0652; Subj: F=1.4, p=0.119, η_p^2=0.596, F0>L0								
3α,5α-THA/3α,5α-THP	43	2.93 (2.5, 3.44)	43	2.35 (2, 2.76)	14	0.361 (0.224, 0.546)	17	0.554 (0.385, 0.775)
Stage: F=0.3, p=0.616, η_p^2=0.00469; PMC: F=47.3, p<0.001, η_p^2=0.467; Stage × PMC: F=3.4, p=0.069, η_p^2=0.0598; Subj: F=1.7, p=0.028, η_p^2=0.647, F0>L0								
3α,5α-THA/3α,5α-THP, C	43	222 (182, 270)	41	276 (225, 339)	13	27.5 (13.3, 48.7)	17	55.7 (34.6, 85.8)
Stage: F=4.8, p=0.034, η_p^2=0.0837; PMC: F=30.5, p<0.001, η_p^2=0.37; Stage × PMC: F=0.7, p=0.415, η_p^2=0.0128; Subj: F=2.1, p=0.004, η_p^2=0.7, F0>L0								
3α,5α-THA/3α,5α,20α-PD	44	0.801 (0.703, 0.914)	43	0.597 (0.524, 0.683)	14	0.255 (0.189, 0.34)	17	0.204 (0.155, 0.265)
Stage: F=5, p=0.03, η_p^2=0.0831; PMC: F=29.6, p<0.001, η_p^2=0.35; Stage × PMC: F=0.1, p=0.773, η_p^2=0.00153; Subj: F=2.7, p<0.001, η_p^2=0.741, F0>F1, F0>L0								
3α,5α-THA/3α,5α,20α-PD, C	42	38.9 (33.5, 45.1)	41	36.6 (31.3, 42.7)	13	3.9 (2.01, 6.55)	16	5.6 (3.4, 8.58)
Stage: F=0.4, p=0.543, η_p^2=0.00731; PMC: F=52.8, p<0.001, η_p^2=0.509; Stage × PMC: F=0.9, p=0.348, η_p^2=0.0173; Subj: F=2.9, p<0.001, η_p^2=0.765, F0>L0								
3β,5α-THA/3β,5α-THP	44	1.71 (1.47, 2.01)	43	0.945 (0.806, 1.11)	14	0.511 (0.353, 0.727)	17	0.608 (0.441, 0.829)
Stage: F=2.5, p=0.122, η_p^2=0.0429; PMC: F=11.2, p=0.002, η_p^2=0.169; Stage × PMC: F=6.1, p=0.016, η_p^2=0.1; Subj: F=1.5, p=0.084, η_p^2=0.608, F0>F1, F0>L0								
3β,5α-THA/3β,5α-THP, C	43	27.2 (23.8, 31.2)	42	24 (20.9, 27.6)	13	10.8 (7.8, 14.8)	16	11.2 (8.41, 14.8)
Stage: F=0.1, p=0.712, η_p^2=0.00259; PMC: F=15.4, p<0.001, η_p^2=0.225; Stage × PMC: F=0.3, p=0.565, η_p^2=0.00628; Subj: F=3.1, p<0.001, η_p^2=0.767, F0>L0								
3β,5α-THA/3β,5α,20α-PD	38	1.22 (0.858, 1.72)	42	2.37 (1.71, 3.27)	14	0.815 (0.376, 1.65)	17	1.32 (0.691, 2.45)
Stage: F=4.2, p=0.047, η_p^2=0.0799; PMC: F=1, p=0.325, η_p^2=0.0202; Stage × PMC: F=0.1, p=0.758, η_p^2=0.002; Subj: F=1.6, p=0.057, η_p^2=0.657								
3β,5α-THA/3β,5α,20α-PD, C	43	2.69 (2.16, 3.33)	42	4.74 (3.81, 5.9)	13	1.19 (0.686, 1.99)	16	1.57 (0.984, 2.44)
Stage: F=4.5, p=0.039, η_p^2=0.078; PMC: F=7.7, p=0.008, η_p^2=0.127; Stage × PMC: F=0.5, p=0.483, η_p^2=0.00933; Subj: F=2.5, p<0.001, η_p^2=0.729, F0<F1, F0>L0								
3α,5β-THA/3α,5β-THP	42	2.86 (2.34, 3.51)	43	2.5 (2.04, 3.06)	14	0.359 (0.213, 0.568)	17	0.478 (0.313, 0.71)
Stage: F=0.1, p=0.706, η_p^2=0.00271; PMC: F=36.7, p<0.001, η_p^2=0.409; Stage × PMC: F=1.1, p=0.311, η_p^2=0.0194; Subj: F=1.4, p=0.1, η_p^2=0.612, F0>L0								
3α,5β-THA/3α,5β-THP, C	42	5.59 (4.99, 6.27)	41	5.52 (4.9, 6.22)	13	1.87 (1.38, 2.48)	16	2.53 (1.97, 3.21)
Stage: F=1.6, p=0.209, η_p^2=0.0302; PMC: F=25.7, p<0.001, η_p^2=0.331; Stage × PMC: F=1.5, p=0.226, η_p^2=0.0281; Subj: F=2.9, p<0.001, η_p^2=0.758, F0>L0								
3α,5β-THA/3α,5β,20α-PD	44	0.833 (0.687, 1.02)	43	0.723 (0.594, 0.884)	14	0.228 (0.15, 0.341)	17	0.222 (0.153, 0.319)
Stage: F=0.2, p=0.624, η_p^2=0.0044; PMC: F=18, p<0.001, η_p^2=0.246; Stage × PMC: F=0.1, p=0.764, η_p^2=0.00165; Subj: F=1.2, p=0.212, η_p^2=0.571, F0>L0								
3α,5β-THA/3α,5β,20α-PD, C	39	3.06 (2.78, 3.36)	39	2.04 (1.83, 2.27)	13	0.469 (0.308, 0.669)	15	0.389 (0.254, 0.554)
Stage: F=8.4, p=0.006, η_p^2=0.146; PMC: F=76.8, p<0.001, η_p^2=0.61; Stage × PMC: F=2.3, p=0.132, η_p^2=0.0456; Subj: F=3.8, p<0.001, η_p^2=0.805, F0>F1, F0>L0								
3β,5β-THA/3β,5β-THP, C	42	8.7 (7.44, 10.2)	41	8.65 (7.36, 10.2)	13	2.58 (1.71, 3.79)	16	3.33 (2.36, 4.62)
Stage: F=0.6, p=0.429, η_p^2=0.0121; PMC: F=18.4, p<0.001, η_p^2=0.261; Stage × PMC: F=0.5, p=0.467, η_p^2=0.0102; Subj: F=2.8, p<0.001, η_p^2=0.748, F0>L0								
3β,5β-THA/3β,5β,20α-PD, C	42	1.59 (1.36, 1.85)	41	1.48 (1.26, 1.73)	12	0.291 (0.174, 0.456)	15	0.56 (0.385, 0.795)
Stage: F=2.6, p=0.117, η_p^2=0.0476; PMC: F=25, p<0.001, η_p^2=0.329; Stage × PMC: F=3.3, p=0.074, η_p^2=0.0612; Subj: F=3.3, p<0.001, η_p^2=0.778, F0>L0								
11β-OH-A/B	41	4.32 (3.79, 4.94)	40	4.73 (4.12, 5.46)	14	3.83 (2.93, 5.07)	17	5.04 (3.92, 6.61)
Stage: F=2.6, p=0.113, η_p^2=0.0476; PMC: F=0, p=0.879, η_p^2=0.000451; Stage × PMC: F=0.5, p=0.48, η_p^2=0.00965; Subj: F=2.1, p=0.004, η_p^2=0.693								

Table S1, continued. Changes of steroids, steroid molar ratios that may reflect activities of steroidogenic enzymes and balances between them, and of indices of MS severity after treatment with anti-MS drugs shown as re-transformed means with their 95% confidence intervals

	Follicular phase				Luteal phase			
Variable	n	Before treatment	n	After treatment	n	Before treatment	n	After treatment
**C17-hydroxylase, C17,20-lyase (CYP17A1), hydroxylase step**								
17-OH-Preg/Preg	44	2.62 (2.22, 3.1)	43	1.91 (1.6, 2.27)	14	1.42 (0.972, 2.05)	17	1.45 (1.04, 2.02)
Stage: F=1, p=0.324, η_p^2=0.0177; PMC: F=2.8, p=0.1, η_p^2=0.0484; Stage × PMC: F=1, p=0.312, η_p^2=0.0186; Subj: F=0.8, p=0.743, η_p^2=0.474, F0>L0								
10³·17-OH-Preg/Preg, C	42	79.3 (71.2, 88.2)	40	56.1 (49.6, 63.2)	13	56 (43.1, 71.6)	17	72.4 (58.4, 89)
Stage: F=0.2, p=0.648, η_p^2=0.00412; PMC: F=0.1, p=0.779, η_p^2=0.00156; Stage × PMC: F=7.1, p=0.01, η_p^2=0.122; Subj: F=1.4, p=0.104, η_p^2=0.613, F0>F1								
17-OH-P/P	44	5.64 (4.52, 7.01)	43	3.73 (2.93, 4.71)	14	0.275 (0.054, 0.644)	17	0.458 (0.188, 0.881)
Stage: F=0.1, p=0.732, η_p^2=0.00215; PMC: F=33.2, p<0.001, η_p^2=0.377; Stage × PMC: F=2.4, p=0.128, η_p^2=0.0416; Subj: F=0.9, p=0.62, η_p^2=0.497, F0>L0								
17-OH-20α-DHP/20α-DHP	43	2 (1.75, 2.27)	42	1.91 (1.67, 2.19)	13	0.34 (0.18, 0.551)	16	0.439 (0.273, 0.651)
Stage: F=0.2, p=0.653, η_p^2=0.00386; PMC: F=38.9, p<0.001, η_p^2=0.423; Stage × PMC: F=0.5, p=0.483, η_p^2=0.00934; Subj: F=1.7, p=0.025, η_p^2=0.648, F0>L0								
10³·3α,5α,17-PD/3α,5α-THP, C	44	332 (295, 371)	41	275 (239, 313)	12	106 (62.4, 161)	17	139 (96.3, 191)
Stage: F=0, p=0.983, η_p^2=0.00000906; PMC: F=14.3, p<0.001, η_p^2=0.216; Stage × PMC: F=2.2, p=0.141, η_p^2=0.0412; Subj: F=2, p=0.007, η_p^2=0.689, F0>L0								
3α,5β,17-PD/3α,5β-THP	38	0.584 (0.467, 0.728)	42	0.447 (0.359, 0.554)	14	0.292 (0.18, 0.463)	17	0.258 (0.166, 0.394)
Stage: F=1.1, p=0.298, η_p^2=0.0225; PMC: F=3.6, p=0.065, η_p^2=0.0694; Stage × PMC: F=0.1, p=0.72, η_p^2=0.00271; Subj: F=2, p=0.007, η_p^2=0.713, F0>L0								
3α,5β,17-PD/3α,5β-THP, C	43	0.623 (0.553, 0.705)	42	0.425 (0.379, 0.478)	13	0.358 (0.28, 0.461)	16	0.328 (0.262, 0.411)
Stage: F=5.2, p=0.027, η_p^2=0.0903; PMC: F=5, p=0.03, η_p^2=0.087; Stage × PMC: F=1.5, p=0.229, η_p^2=0.0277; Subj: F=1.8, p=0.017, η_p^2=0.666, F0>F1, F0>L0								
10³·3α,5α,17α,20α-PT/3α,5α,20α-PD	43	129 (110, 153)	43	144 (121, 171)	13	89.4 (62.9, 129)	15	37.5 (27, 51.5)
Stage: F=7.8, p=0.007, η_p^2=0.128; PMC: F=11.3, p=0.002, η_p^2=0.175; Stage × PMC: F=9.5, p=0.003, η_p^2=0.152; Subj: F=3.3, p<0.001, η_p^2=0.781, L0>L1, F1>L1								
3α,5α,17α,20α-PT/3α,5α,20α-PD, C	44	0.971 (0.724, 1.31)	42	0.292 (0.22, 0.39)	13	0.152 (0.0805, 0.281)	17	0.115 (0.0646, 0.198)
Stage: F=8.3, p=0.006, η_p^2=0.135; PMC: F=10.1, p=0.002, η_p^2=0.161; Stage × PMC: F=2.4, p=0.125, η_p^2=0.0437; Subj: F=2.1, p=0.004, η_p^2=0.7, F0>F1, F0>L0								
3α,5β,17α,20α-PT/3α,5β,20α-PD	44	5.96 (4.9, 7.33)	43	5.14 (4.24, 6.3)	14	2.36 (1.66, 3.41)	17	1.92 (1.41, 2.65)
Stage: F=1.3, p=0.256, η_p^2=0.0234; PMC: F=12, p=0.001, η_p^2=0.179; Stage × PMC: F=0.1, p=0.797, η_p^2=0.00122; Subj: F=0.9, p=0.712, η_p^2=0.481, F0>L0								
3α,5β,17α,20α-PT/3α,5β,20α-PD, C	44	4.2 (3.46, 5.07)	41	1.48 (1.16, 1.87)	12	0.623 (0.31, 1.13)	17	0.586 (0.327, 0.974)
Stage: F=8.2, p=0.006, η_p^2=0.136; PMC: F=14.3, p<0.001, η_p^2=0.215; Stage × PMC: F=5.5, p=0.023, η_p^2=0.0952; Subj: F=2.3, p=0.001, η_p^2=0.721, F0>F1, F0>L0								
F/B	43	30.7 (27, 35.1)	42	39.5 (34.2, 45.9)	14	37.3 (27.8, 51.3)	17	35.7 (27.5, 47.3)
Stage: F=0.8, p=0.384, η_p^2=0.0141; PMC: F=0.1, p=0.822, η_p^2=0.00095; Stage × PMC: F=1.2, p=0.284, η_p^2=0.0213; Subj: F=1.6, p=0.038, η_p^2=0.635								
F (RIA)/B	44	42.4 (36.7, 49.2)	42	62.2 (52.8, 73.9)	13	79.3 (53.8, 124)	15	45.6 (33.5, 63.3)
Stage: F=0.3, p=0.603, η_p^2=0.00525; PMC: F=0.3, p=0.568, η_p^2=0.00632; Stage × PMC: F=8.5, p=0.005, η_p^2=0.14; Subj: F=1.5, p=0.061, η_p^2=0.63, F0<F1, F0<L0								
**C17-hydroxylase, C17,20-lyase (CYP17A1), lyase step**								
DHEA/17-OH-Preg	44	1.32 (1.19, 1.47)	43	1.75 (1.57, 1.96)	14	1.84 (1.45, 2.35)	17	1.77 (1.44, 2.2)
Stage: F=1.7, p=0.196, η_p^2=0.0302; PMC: F=1, p=0.313, η_p^2=0.0185; Stage × PMC: F=2.2, p=0.14, η_p^2=0.0392; Subj: F=2.2, p=0.002, η_p^2=0.697, F0<F1								
DHEA/17-OH-Preg, C	44	238 (209, 272)	41	298 (259, 342)	12	397 (293, 533)	17	287 (220, 371)
Stage: F=0.2, p=0.647, η_p^2=0.00407; PMC: F=1.2, p=0.272, η_p^2=0.0232; Stage × PMC: F=4.2, p=0.046, η_p^2=0.0747; Subj: F=3.4, p<0.001, η_p^2=0.79, F0<L0								
A/17-OH-P	44	2.57 (2.3, 2.87)	43	3.03 (2.71, 3.38)	14	0.994 (0.725, 1.32)	17	1.27 (0.981, 1.62)
Stage: F=3.7, p=0.059, η_p^2=0.0634; PMC: F=24, p<0.001, η_p^2=0.304; Stage × PMC: F=0, p=0.859, η_p^2=0.00058; Subj: F=2.8, p<0.001, η_p^2=0.753, F0>L0								
A/17-OH-20α-DHP	44	4.48 (3.94, 5.07)	43	3.97 (3.46, 4.54)	14	2.21 (1.52, 3.07)	17	2.79 (2.06, 3.68)
Stage: F=0.1, p=0.725, η_p^2=0.00227; PMC: F=5.8, p=0.019, η_p^2=0.0957; Stage × PMC: F=1.6, p=0.215, η_p^2=0.0278; Subj: F=1.4, p=0.097, η_p^2=0.603, F0>L0								
10-3·3α,5α-THA/3α,5α,17-PD, C	42	0.692 (0.568, 0.844)	41	1.27 (1.03, 1.56)	13	0.292 (0.182, 0.459)	16	0.444 (0.297, 0.659)
Stage: F=8, p=0.007, η_p^2=0.134; PMC: F=9.4, p=0.003, η_p^2=0.153; Stage × PMC: F=0.2, p=0.631, η_p^2=0.00447; Subj: F=2.6, p<0.001, η_p^2=0.736, F0<F1, F0>L0								

Table S1, continued. Changes of steroids, steroid molar ratios that may reflect activities of steroidogenic enzymes and balances between them, and of indices of MS severity after treatment with anti-MS drugs shown as re-transformed means with their 95% confidence intervals

	Follicular phase				Luteal phase			
Variable	n	Before treatment	n	After treatment	n	Before treatment	n	After treatment
**C17-hydroxylase, C17,20-lyase (CYP17A1), lyase step**								

3α,5β-THA/3α,5β,17-PD	39	4.38 (3.55, 5.46)	42	5.37 (4.33, 6.72)	14	1.24 (0.823, 1.85)	17	1.71 (1.19, 2.46)
Stage: F=2.4, p=0.125, η_p^2=0.0473; PMC: F=16.5, p<0.001, η_p^2=0.252; Stage × PMC: F=0.2, p=0.697, η_p^2=0.00311; Subj: F=1.8, p=0.02, η_p^2=0.682, F0>L0								
3α,5β-THA/3α,5β,17-PD, C	39	8.4 (7.6, 9.27)	38	13.5 (12.2, 15)	12	6.24 (4.93, 7.84)	15	7.77 (6.35, 9.49)
Stage: F=15.4, p<0.001, η_p^2=0.243; PMC: F=6.9, p=0.011, η_p^2=0.126; Stage × PMC: F=1.7, p=0.198, η_p^2=0.0343; Subj: F=5.5, p<0.001, η_p^2=0.857, F0<F1								
3α,5α-THA/3α,5α,17α,20α-PT	42	5.43 (4.7, 6.26)	42	4.01 (3.46, 4.64)	14	2.79 (2.01, 3.84)	16	4.13 (3.08, 5.51)
Stage: F=0.1, p=0.745, η_p^2=0.00202; PMC: F=1.9, p=0.175, η_p^2=0.0345; Stage × PMC: F=6, p=0.018, η_p^2=0.102; Subj: F=2.3, p=0.002, η_p^2=0.708, F0>F1, F0>L0								
3α,5α-THA/3α,5α,17α,20α-PT, C	42	32.2 (24, 42.9)	40	126 (92.1, 172)	13	37.9 (19.7, 72.1)	16	62.3 (35.2, 111)
Stage: F=12.6, p<0.001, η_p^2=0.195; PMC: F=0.3, p=0.562, η_p^2=0.00652; Stage × PMC: F=2.1, p=0.156, η_p^2=0.0383; Subj: F=2.7, p<0.001, η_p^2=0.743, F0<F1								
103·3α,5β-THA/3α,5β,17α,20α-PT	43	137 (124, 151)	42	129 (116, 143)	14	91 (73.9, 112)	17	112 (92.7, 136)
Stage: F=0.8, p=0.373, η_p^2=0.0147; PMC: F=3.2, p=0.08, η_p^2=0.0556; Stage × PMC: F=1.9, p=0.174, η_p^2=0.034; Subj: F=2.7, p<0.001, η_p^2=0.745, F0>L0								
3α,5β-THA/3α,5β,17α,20α-PT, C	42	0.569 (0.482, 0.674)	40	1.62 (1.31, 2.04)	13	0.509 (0.354, 0.744)	16	0.62 (0.446, 0.877)
Stage: F=14.1, p<0.001, η_p^2=0.214; PMC: F=3.4, p=0.07, η_p^2=0.0616; Stage × PMC: F=4.7, p=0.035, η_p^2=0.0825; Subj: F=2.8, p<0.001, η_p^2=0.748, F0<F1								
103·11β-OH-A/F	44	130 (118, 144)	43	134 (121, 148)	13	116 (91, 147)	16	125 (101, 153)
Stage: F=0.3, p=0.592, η_p^2=0.00537; PMC: F=0.3, p=0.574, η_p^2=0.00589; Stage × PMC: F=0, p=0.847, η_p^2=0.000695; Subj: F=1.1, p=0.417, η_p^2=0.532								
103·11β-OH-A/F (RIA)	44	92.5 (82.9, 103)	42	89.4 (79.5, 99.9)	12	61.1 (43.2, 82.5)	14	92.5 (72.1, 116)
Stage: F=2.7, p=0.105, η_p^2=0.0506; PMC: F=0.9, p=0.354, η_p^2=0.0169; Stage × PMC: F=3.2, p=0.082, η_p^2=0.0583; Subj: F=1.3, p=0.163, η_p^2=0.595, F0>L0								
**3β-Hydroxysteroid dehydrogenases (HSD3Bs)**								
103·P/Preg	44	86 (69.6, 106)	43	79.1 (63.7, 98.6)	14	4120 (1480, 18200)	17	1760 (836, 4580)
Stage: F=1.5, p=0.222, η_p^2=0.027; PMC: F=56.8, p<0.001, η_p^2=0.508; Stage × PMC: F=0.5, p=0.487, η_p^2=0.00884; Subj: F=1.1, p=0.427, η_p^2=0.53, F0<L0								
103·P/PregC	44	1.42 (1.11, 1.82)	42	1.61 (1.24, 2.1)	13	101 (30.4, 579)	17	47.8 (19.4, 155)
Stage: F=0.2, p=0.655, η_p^2=0.0038; PMC: F=48.8, p<0.001, η_p^2=0.479; Stage × PMC: F=0.8, p=0.377, η_p^2=0.0147; Subj: F=1, p=0.446, η_p^2=0.536, F0<L0								
103·20α-DHP/20α-DHPreg	44	91.2 (79.3, 105)	43	93.6 (80.9, 109)	14	1010 (576, 2020)	17	640 (411, 1070)
Stage: F=0.8, p=0.373, η_p^2=0.0145; PMC: F=54.6, p<0.001, η_p^2=0.498; Stage × PMC: F=1, p=0.332, η_p^2=0.0171; Subj: F=1.8, p=0.015, η_p^2=0.658, F0<L0								
103·20α-DHP/20α-DHPreg, C	43	1.25 (1, 1.55)	42	0.945 (0.746, 1.19)	13	2.58 (1.6, 4.15)	16	3.69 (2.4, 5.68)
Stage: F=0.1, p=0.812, η_p^2=0.0011; PMC: F=9.1, p=0.004, η_p^2=0.149; Stage × PMC: F=2.1, p=0.153, η_p^2=0.0389; Subj: F=1.3, p=0.174, η_p^2=0.59, F0<L0								
17-OH-P/17-OH-Preg	44	0.175 (0.152, 0.201)	43	0.144 (0.125, 0.165)	14	0.662 (0.438, 1.08)	17	0.507 (0.36, 0.748)
Stage: F=2.6, p=0.116, η_p^2=0.0443; PMC: F=26.9, p<0.001, η_p^2=0.328; Stage × PMC: F=0, p=0.984, η_p^2=0.00000765; Subj: F=2.2, p=0.002, η_p^2=0.698, F0<L0								
17-OH-P/17-OH-PregC	42	0.0887 (0.0764, 0.103)	41	0.094 (0.0805, 0.11)	13	0.621 (0.403, 0.997)	16	0.554 (0.376, 0.843)
Stage: F=0, p=0.922, η_p^2=0.000188; PMC: F=47.4, p<0.001, η_p^2=0.481; Stage × PMC: F=0.2, p=0.64, η_p^2=0.00433; Subj: F=2.6, p<0.001, η_p^2=0.745, F0<L0								
16α-OH-P/16α-OH-Preg	44	0.698 (0.611, 0.801)	43	0.577 (0.504, 0.662)	14	2.59 (1.72, 4.21)	17	1.99 (1.42, 2.92)
Stage: F=2.6, p=0.116, η_p^2=0.0443; PMC: F=26.9, p<0.001, η_p^2=0.328; Stage × PMC: F=0, p=0.984, η_p^2=0.00000765; Subj: F=2.2, p=0.002, η_p^2=0.698, F0<L0								
A/DHEA	42	0.331 (0.302, 0.363)	41	0.256 (0.235, 0.28)	13	0.341 (0.279, 0.423)	16	0.394 (0.326, 0.48)
Stage: F=0.6, p=0.448, η_p^2=0.0111; PMC: F=2.6, p=0.11, η_p^2=0.0483; Stage × PMC: F=4.6, p=0.037, η_p^2=0.0814; Subj: F=2.6, p<0.001, η_p^2=0.739, F0>F1								
103·A/DHEAC	44	1.05 (0.907, 1.22)	42	0.951 (0.811, 1.11)	13	1.78 (1.26, 2.52)	17	1.97 (1.46, 2.67)
Stage: F=0, p=0.992, η_p^2=0.00000199; PMC: F=6.9, p=0.011, η_p^2=0.115; Stage × PMC: F=0.4, p=0.517, η_p^2=0.00796; Subj: F=1.9, p=0.009, η_p^2=0.679, F0<L0								
T/Adiol	44	0.296 (0.263, 0.331)	43	0.342 (0.304, 0.383)	14	0.286 (0.218, 0.368)	17	0.412 (0.332, 0.505)
Stage: F=6.6, p=0.013, η_p^2=0.108; PMC: F=0.2, p=0.648, η_p^2=0.00382; Stage × PMC: F=1, p=0.321, η_p^2=0.0179; Subj: F=2.3, p<0.001, η_p^2=0.714								
103·T/AdiolC	44	0.61 (0.51, 0.728)	42	0.805 (0.668, 0.969)	13	1.36 (0.909, 2.05)	17	1.69 (1.18, 2.43)
Stage: F=2.3, p=0.138, η_p^2=0.0411; PMC: F=7.5, p=0.008, η_p^2=0.124; Stage × PMC: F=0, p=0.859, η_p^2=0.000602; Subj: F=2.3, p=0.001, η_p^2=0.719, F0<L0								

Table S1, continued. Changes of steroids, steroid molar ratios that may reflect activities of steroidogenic enzymes and balances between them, and of indices of MS severity after treatment with anti-MS drugs shown as re-transformed means with their 95% confidence intervals

	Follicular phase				Luteal phase			
Variable	n	Before treatment	n	After treatment	n	Before treatment	n	After treatment
**Conjugated/unconjugated steroids (sulfotransferase 2A1/steroid sulfatase, SULT2A1/STS)**								
Preg C/U	44	58.9 (50.2, 69.5)	42	46.3 (39.3, 54.8)	13	31.9 (22.8, 45.2)	17	25.7 (19.2, 34.5)
Stage: F=2.6, p=0.11, η_p^2=0.0475; PMC: F=6.1, p=0.017, η_p^2=0.104; Stage × PMC: F=0, p=0.987, η_p^2=0.0000048; Subj: F=1.7, p=0.024, η_p^2=0.656, F0>L0								
20α-DHPreg C/U	44	231 (202, 264)	42	196 (171, 225)	13	148 (112, 197)	17	118 (92.7, 152)
Stage: F=2.8, p=0.1, η_p^2=0.0502; PMC: F=5.6, p=0.022, η_p^2=0.0951; Stage × PMC: F=0.1, p=0.789, η_p^2=0.00136; Subj: F=1.6, p=0.054, η_p^2=0.633, F0>L0								
17-OH-Preg C/U	42	1.59 (1.41, 1.82)	42	1.32 (1.18, 1.48)	13	1.28 (1.02, 1.66)	15	1.19 (0.963, 1.51)
Stage: F=1.7, p=0.201, η_p^2=0.0319; PMC: F=0.8, p=0.391, η_p^2=0.0145; Stage × PMC: F=0.2, p=0.636, η_p^2=0.00442; Subj: F=2.5, p<0.001, η_p^2=0.734								
DHEA C/U	44	302 (259, 353)	42	242 (208, 282)	13	207 (152, 289)	17	180 (137, 238)
Stage: F=1.9, p=0.173, η_p^2=0.0347; PMC: F=2.2, p=0.148, η_p^2=0.0391; Stage × PMC: F=0.1, p=0.831, η_p^2=0.000869; Subj: F=1.5, p=0.058, η_p^2=0.63								
Adiol C/U	44	485 (418, 561)	42	421 (359, 492)	13	226 (154, 324)	17	241 (173, 330)
Stage: F=0.1, p=0.771, η_p^2=0.00162; PMC: F=7.3, p=0.009, η_p^2=0.121; Stage × PMC: F=0.4, p=0.515, η_p^2=0.00805; Subj: F=1.9, p=0.008, η_p^2=0.682, F0>L0								
3β,16α,17β-AT C/U	44	415 (339, 504)	42	350 (281, 432)	13	212 (124, 344)	17	209 (131, 320)
Stage: F=0.3, p=0.615, η_p^2=0.0048; PMC: F=3.2, p=0.081, η_p^2=0.0563; Stage × PMC: F=0.1, p=0.709, η_p^2=0.00264; Subj: F=1.2, p=0.263, η_p^2=0.569								
20α-DHP C/U	43	3.6 (2.92, 4.46)	42	2.12 (1.71, 2.64)	13	0.262 (0.139, 0.45)	16	0.336 (0.198, 0.54)
Stage: F=0.7, p=0.411, η_p^2=0.0131; PMC: F=40.5, p<0.001, η_p^2=0.438; Stage × PMC: F=3, p=0.09, η_p^2=0.0542; Subj: F=2, p=0.006, η_p^2=0.692, F0>F1, F0>L0								
5α-DHT C/U	43	6.28 (4.74, 8.39)	42	3.97 (2.98, 5.31)	13	9.58 (5.06, 19)	17	3.5 (2, 6.12)
Stage: F=8.4, p=0.006, η_p^2=0.139; PMC: F=0.1, p=0.757, η_p^2=0.00186; Stage × PMC: F=0.9, p=0.347, η_p^2=0.017; Subj: F=1.4, p=0.137, η_p^2=0.605								
3α,5α-THP C/U	42	20.7 (17.2, 25.2)	41	14.1 (11.8, 16.9)	12	13.3 (9.06, 20.4)	16	12.4 (8.9, 17.8)
Stage: F=2, p=0.169, η_p^2=0.0376; PMC: F=0.9, p=0.348, η_p^2=0.0176; Stage × PMC: F=0.7, p=0.404, η_p^2=0.014; Subj: F=1.6, p=0.041, η_p^2=0.649, F0>F1								
3β,5α-THP C/U	42	49.8 (41.9, 60)	41	32.7 (28.2, 38.4)	12	23.1 (17.3, 31.8)	15	31.1 (23.3, 43.2)
Stage: F=0.1, p=0.827, η_p^2=0.000969; PMC: F=2.8, p=0.098, η_p^2=0.0537; Stage × PMC: F=5, p=0.031, η_p^2=0.0902; Subj: F=1.8, p=0.022, η_p^2=0.664, F0>F1, F0>L0								
3α,5β-THP C/U	42	49.8 (41.9, 60)	41	32.7 (28.2, 38.4)	12	23.1 (17.3, 31.8)	15	31.1 (23.3, 43.2)
Stage: F=0.1, p=0.827, η_p^2=0.000969; PMC: F=2.8, p=0.098, η_p^2=0.0537; Stage × PMC: F=5, p=0.031, η_p^2=0.0902; Subj: F=1.8, p=0.022, η_p^2=0.664, F0>F1, F0>L0								
3α,5β,17-PD C/U	38	157 (121, 206)	41	134 (105, 174)	14	59.5 (38.6, 94.6)	17	48.4 (33.1, 72.3)
Stage: F=0.9, p=0.349, η_p^2=0.0183; PMC: F=8.5, p=0.005, η_p^2=0.151; Stage × PMC: F=0, p=0.853, η_p^2=0.000718; Subj: F=1.7, p=0.025, η_p^2=0.677, F0>L0								
5α,20α-THP C/U	44	0.818 (0.64, 1.06)	42	0.483 (0.383, 0.616)	13	0.539 (0.327, 0.942)	17	0.534 (0.344, 0.866)
Stage: F=1.5, p=0.223, η_p^2=0.0279; PMC: F=0.2, p=0.687, η_p^2=0.00309; Stage × PMC: F=1.1, p=0.3, η_p^2=0.0202; Subj: F=1.6, p=0.05, η_p^2=0.635, F0>F1								
3α,5α,20α-PD C/U	44	45.3 (38, 53.9)	42	33.2 (27.7, 39.8)	13	51.4 (34.6, 77.2)	17	32.6 (23.2, 46)
Stage: F=5.9, p=0.019, η_p^2=0.0995; PMC: F=0, p=0.847, η_p^2=0.00071; Stage × PMC: F=0.2, p=0.689, η_p^2=0.00304; Subj: F=2.8, p<0.001, η_p^2=0.757								
3β,5α,20α-PD C/U	38	302 (223, 417)	41	404 (295, 565)	13	299 (161, 599)	17	322 (185, 598)
Stage: F=0.5, p=0.504, η_p^2=0.00975; PMC: F=0.1, p=0.802, η_p^2=0.00139; Stage × PMC: F=0.1, p=0.733, η_p^2=0.00255; Subj: F=0.8, p=0.825, η_p^2=0.498								
3α,5β,20α-PD C/U	44	116 (95.3, 141)	42	105 (85.3, 129)	13	82.4 (53.7, 128)	17	85.2 (58.5, 126)
Stage: F=0, p=0.849, η_p^2=0.000693; PMC: F=0.8, p=0.377, η_p^2=0.0148; Stage × PMC: F=0.1, p=0.736, η_p^2=0.00216; Subj: F=2.1, p=0.004, η_p^2=0.696								
3β,5β,20α-PD C/U	44	68.2 (53.8, 86.1)	42	66.4 (51.7, 84.8)	13	154 (92, 254)	17	83.3 (52.1, 131)
Stage: F=2.4, p=0.129, η_p^2=0.043; PMC: F=2.1, p=0.158, η_p^2=0.0373; Stage × PMC: F=1.6, p=0.218, η_p^2=0.0285; Subj: F=2.6, p<0.001, η_p^2=0.742, F0<L0								
3α,5α,17,20α-PT C/U	44	317 (229, 444)	43	73.2 (53.7, 100)	14	95.7 (49.1, 190)	17	70.9 (39.1, 130)
Stage: F=10.3, p=0.002, η_p^2=0.158; PMC: F=1.5, p=0.224, η_p^2=0.0267; Stage × PMC: F=3.4, p=0.07, η_p^2=0.0584; Subj: F=1.5, p=0.08, η_p^2=0.61, F0>F1, F0>L0								
3α,5β,17,20α-PT C/U	44	75.8 (62.8, 91)	43	25.9 (20.1, 32.9)	14	43.2 (26.4, 67.2)	17	31.1 (19.4, 47.7)
Stage: F=14.2, p<0.001, η_p^2=0.205; PMC: F=0.4, p=0.516, η_p^2=0.0077; Stage × PMC: F=3.4, p=0.072, η_p^2=0.0577; Subj: F=1.2, p=0.294, η_p^2=0.554, F0>F1								

Table S1, continued. Changes of steroids, steroid molar ratios that may reflect activities of steroidogenic enzymes and balances between them, and of indices of MS severity after treatment with anti-MS drugs

	**Follicular phase**				**Luteal phase**			
Variable	n	Before treatment	n	After treatment	n	Before treatment	n	After treatment
**Conjugated/unconjugated steroids (sulfotransferase 2A1/steroid sulfatase, SULT2A1/STS)**								
10-3·3α,5α-THA C/U	41	2.04 (1.7, 2.42)	41	2.5 (2.1, 2.95)	13	0.905 (0.541, 1.41)	15	0.842 (0.52, 1.28)
Stage: F=0.2, p=0.634, η_p^2=0.00448; PMC: F=9.9, p=0.003, η_p^2=0.163; Stage × PMC: F=0.6, p=0.454, η_p^2=0.011; Subj: F=1.7, p=0.024, η_p^2=0.652, F0>L0								
10-3·3β,5α-THA C/U	41	0.895 (0.766, 1.04)	40	0.828 (0.701, 0.97)	12	0.781 (0.522, 1.11)	15	0.679 (0.47, 0.943)
Stage: F=0.6, p=0.458, η_p^2=0.0111; PMC: F=0.4, p=0.53, η_p^2=0.00795; Stage × PMC: F=0, p=0.863, η_p^2=0.000602; Subj: F=2.1, p=0.005, η_p^2=0.69								
10-3·3α,5β-THA C/U	41	0.35 (0.307, 0.396)	40	0.315 (0.274, 0.36)	13	0.216 (0.151, 0.296)	16	0.175 (0.124, 0.238)
Stage: F=1.4, p=0.243, η_p^2=0.0266; PMC: F=5.9, p=0.019, η_p^2=0.103; Stage × PMC: F=0.1, p=0.807, η_p^2=0.00118; Subj: F=1.2, p=0.224, η_p^2=0.571, F0>L0								
3α,5α,17β-AD C/U	39	328 (288, 371)	39	271 (237, 308)	13	131 (92.7, 179)	15	104 (73.5, 143)
Stage: F=2.9, p=0.096, η_p^2=0.0566; PMC: F=17.8, p<0.001, η_p^2=0.27; Stage × PMC: F=0, p=0.973, η_p^2=0.0000239; Subj: F=3.9, p<0.001, η_p^2=0.813, F0>L0								
11β-OH-3α,5α-THA C/U	43	16.7 (14.6, 19.1)	42	18.3 (15.9, 21)	14	12.5 (9.14, 16.9)	17	11.6 (8.72, 15.2)
Stage: F=0, p=0.946, η_p^2=0.0000869; PMC: F=3.1, p=0.086, η_p^2=0.0537; Stage × PMC: F=0.4, p=0.538, η_p^2=0.00706; Subj: F=2.7, p<0.001, η_p^2=0.744								
11β-OH-3β,5α-THA C/U	43	9.28 (7.74, 11.2)	43	10.8 (9, 13.1)	14	9.92 (6.66, 15)	16	13 (8.9, 19.4)
Stage: F=1.7, p=0.195, η_p^2=0.0309; PMC: F=0.2, p=0.682, η_p^2=0.00314; Stage × PMC: F=0.1, p=0.763, η_p^2=0.0017; Subj: F=3.5, p<0.001, η_p^2=0.791								
11β-OH-3α,5β-THA C/U	43	4.38 (3.71, 5.16)	42	5.6 (4.72, 6.63)	14	3.42 (2.36, 4.92)	17	2.55 (1.81, 3.55)
Stage: F=0, p=0.885, η_p^2=0.000391; PMC: F=3.9, p=0.053, η_p^2=0.0677; Stage × PMC: F=2.7, p=0.108, η_p^2=0.0471; Subj: F=4.2, p<0.001, η_p^2=0.818								
**11β-Hydroxylase (CYP11B1)**								
11β-OH-A/A	44	18.4 (15.9, 21.5)	43	16 (13.8, 18.8)	14	9.66 (7.08, 13.3)	17	8.54 (6.47, 11.3)
Stage: F=1, p=0.315, η_p^2=0.0184; PMC: F=7.7, p=0.007, η_p^2=0.123; Stage × PMC: F=0, p=0.981, η_p^2=0.0000109; Subj: F=2.3, p=0.001, η_p^2=0.713, F0>L0								
11β-OH-3α,5α-THA/3α,5α-THA	44	3.41 (2.89, 4.02)	43	2.63 (2.22, 3.12)	14	2.16 (1.49, 3.11)	17	1.47 (1.05, 2.04)
Stage: F=4.8, p=0.033, η_p^2=0.0805; PMC: F=4, p=0.052, η_p^2=0.067; Stage × PMC: F=0.1, p=0.713, η_p^2=0.00248; Subj: F=1.9, p=0.009, η_p^2=0.669								
103·11β-OH-3α,5α-THA/3α,5α-THA, C	40	32.8 (29, 37.2)	39	24.6 (21.8, 28)	12	14.8 (11.2, 19.5)	15	11.1 (8.7, 14.2)
Stage: F=7, p=0.011, η_p^2=0.124; PMC: F=16.5, p<0.001, η_p^2=0.251; Stage × PMC: F=0, p=0.979, η_p^2=0.0000145; Subj: F=7, p<0.001, η_p^2=0.883, F0>F1, F0>L0								
11β-OH-3β,5α-THA/3α,5α-THA	44	0.271 (0.231, 0.317)	43	0.199 (0.167, 0.236)	14	0.275 (0.192, 0.388)	17	0.177 (0.125, 0.246)
Stage: F=6.7, p=0.012, η_p^2=0.108; PMC: F=0, p=0.851, η_p^2=0.000644; Stage × PMC: F=0.2, p=0.693, η_p^2=0.00285; Subj: F=2.6, p<0.001, η_p^2=0.734								
103·11β-OH-3β,5α-THA/3α,5α-THA, C	42	2.76 (2.36, 3.24)	41	2.27 (1.93, 2.68)	13	3.92 (2.74, 5.68)	16	3.76 (2.73, 5.21)
Stage: F=0.7, p=0.415, η_p^2=0.0128; PMC: F=2.9, p=0.095, η_p^2=0.0527; Stage × PMC: F=0.2, p=0.644, η_p^2=0.00413; Subj: F=4, p<0.001, η_p^2=0.811								
11β-OH-3α,5β-THA/3α,5α-THA	44	8.38 (7.41, 9.42)	43	6.36 (5.51, 7.29)	14	5.69 (4.06, 7.64)	17	3.33 (2.25, 4.63)
Stage: F=9.8, p=0.003, η_p^2=0.151; PMC: F=5.1, p=0.028, η_p^2=0.0848; Stage × PMC: F=0.4, p=0.545, η_p^2=0.00671; Subj: F=1.9, p=0.009, η_p^2=0.671, F0>F1								
103·11β-OH-3α,5β-THA/3α,5α-THA, C	42	111 (95.1, 129)	41	108 (92, 127)	13	85.4 (59, 121)	16	55.2 (38.8, 76.9)
Stage: F=2.4, p=0.126, η_p^2=0.0444; PMC: F=3.5, p=0.069, η_p^2=0.0623; Stage × PMC: F=1.4, p=0.237, η_p^2=0.0268; Subj: F=3.6, p<0.001, η_p^2=0.794								

Table S1, continued. Changes of steroids, steroid molar ratios that may reflect activities of steroidogenic enzymes and balances between them, and of indices of MS severity after treatment with anti-MS drugs shown as re-transformed means with their 95% confidence intervals

	Follicular phase				Luteal phase			
Variable	n	Before treatment	n	After treatment	n	Before treatment	n	After treatment
**7α/β- and 16α-hydroxylating enzymes (CYP7B1, CYP3A4, CYP3A7)**								
10³·7α-OH-DHEA/DHEA	44	112 (99.9, 127)	43	70.9 (64.2, 78.7)	14	111 (86.8, 148)	17	72.1 (59.6, 88.3)
Stage: F=22.1, p<0.001, η_p^2=0.287; PMC: F=0, p=0.977, η_p^2=0.000015; Stage × PMC: F=0, p=0.91, η_p^2=0.000236; Subj: F=1.4, p=0.131, η_p^2=0.592, F0>F1								
10³·5-Androstene-3β,7α,17β-triol/Adiol	44	177 (161, 196)	43	160 (145, 177)	14	198 (159, 248)	17	161 (133, 196)
Stage: F=3.2, p=0.082, η_p^2=0.0541; PMC: F=0.1, p=0.72, η_p^2=0.00235; Stage × PMC: F=0.3, p=0.603, η_p^2=0.00495; Subj: F=2.8, p<0.001, η_p^2=0.75								
10³·7β-Hydroxy-DHEA/DHEA	43	51.9 (46.2, 58.7)	42	37.5 (33.6, 42.1)	14	44.2 (34.7, 57.3)	17	35.9 (29.2, 44.6)
Stage: F=7.3, p=0.009, η_p^2=0.119; PMC: F=0.3, p=0.571, η_p^2=0.00599; Stage × PMC: F=0.2, p=0.626, η_p^2=0.00443; Subj: F=1.8, p=0.019, η_p^2=0.653, F0>F1								
10³·3β,7α,17β-AT/Adiol	42	141 (128, 155)	41	105 (94.3, 117)	14	118 (93.9, 145)	17	119 (97.2, 144)
Stage: F=2.7, p=0.108, η_p^2=0.048; PMC: F=0.1, p=0.831, η_p^2=0.000864; Stage × PMC: F=2.3, p=0.132, η_p^2=0.0423; Subj: F=2, p=0.006, η_p^2=0.681, F0>F1								
10³·16α-OH-Preg/Preg	44	169 (147, 194)	43	110 (95.5, 126)	14	119 (88, 161)	17	111 (84.7, 145)
Stage: F=4.3, p=0.042, η_p^2=0.0729; PMC: F=0.6, p=0.435, η_p^2=0.0111; Stage × PMC: F=1.8, p=0.191, η_p^2=0.0308; Subj: F=1.7, p=0.022, η_p^2=0.648, F0>F1								
10³·3β,16α,17β-AT/Adiol	44	86.8 (74.6, 101)	43	103 (88.1, 122)	14	81.1 (58.4, 115)	17	87.1 (64.7, 119)
Stage: F=0.8, p=0.368, η_p^2=0.0147; PMC: F=0.2, p=0.626, η_p^2=0.00436; Stage × PMC: F=0.1, p=0.741, η_p^2=0.00201; Subj: F=2.9, p<0.001, η_p^2=0.755								
10³·3β,16α,17β-AT/Adiol, C	44	74.1 (64.7, 85.2)	42	85.4 (73.8, 99.2)	13	72.3 (53.2, 99.4)	17	70.5 (53.9, 93.2)
Stage: F=0.2, p=0.647, η_p^2=0.004; PMC: F=0.3, p=0.622, η_p^2=0.00461; Stage × PMC: F=0.3, p=0.567, η_p^2=0.00622; Subj: F=3.7, p<0.001, η_p^2=0.806								
16α-OH-P/P	44	1.54 (1.23, 1.91)	43	1.09 (0.861, 1.37)	14	0.094 (0.0306, 0.197)	17	0.119 (0.0519, 0.221)
Stage: F=0.3, p=0.614, η_p^2=0.00467; PMC: F=35.8, p<0.001, η_p^2=0.394; Stage × PMC: F=1.2, p=0.282, η_p^2=0.021; Subj: F=1.3, p=0.179, η_p^2=0.578, F0>L0								
**Type 1 11β-hydroxysteroid dehydrogenase (HSD11B1)**								
7β-OH-DHEA/7α-OH-DHEA	43	0.467 (0.429, 0.507)	42	0.531 (0.488, 0.577)	14	0.393 (0.324, 0.473)	17	0.499 (0.424, 0.583)
Stage: F=6.4, p=0.015, η_p^2=0.105; PMC: F=0.8, p=0.375, η_p^2=0.0146; Stage × PMC: F=0.4, p=0.529, η_p^2=0.00739; Subj: F=1.9, p=0.007, η_p^2=0.676								
3β,7β,17β-AT/3β,7α,17β-AT	41	0.75 (0.689, 0.814)	40	0.678 (0.619, 0.741)	14	0.589 (0.482, 0.71)	17	0.707 (0.599, 0.826)
Stage: F=0.3, p=0.613, η_p^2=0.00497; PMC: F=0.6, p=0.455, η_p^2=0.0108; Stage × PMC: F=2.8, p=0.101, η_p^2=0.051; Subj: F=1.5, p=0.065, η_p^2=0.621								
F/E	43	2.84 (2.41, 3.34)	43	2.57 (2.17, 3.04)	14	2.83 (1.96, 4.02)	17	3.56 (2.59, 4.85)
Stage: F=0.2, p=0.642, η_p^2=0.00403; PMC: F=0.4, p=0.532, η_p^2=0.00727; Stage × PMC: F=1.1, p=0.31, η_p^2=0.0191; Subj: F=1.1, p=0.385, η_p^2=0.542								
F (RIA)/E	41	4.47 (3.97, 5)	41	4.9 (4.36, 5.46)	13	4.08 (3.02, 5.29)	14	3.88 (2.93, 4.97)
Stage: F=0.1, p=0.819, η_p^2=0.00108; PMC: F=0.7, p=0.416, η_p^2=0.0136; Stage × PMC: F=0.4, p=0.545, η_p^2=0.00754; Subj: F=1.8, p=0.022, η_p^2=0.669								
**Aromatase (CYP19A1)**								
10³·E2/A	34	56.7 (44.2, 72.4)	35	52.3 (40.9, 66.5)	8	91.8 (48, 174)	8	87.3 (45.5, 166)
Stage: F=0.1, p=0.808, η_p^2=0.00154; PMC: F=1.3, p=0.267, η_p^2=0.0315; Stage × PMC: F=0, p=0.962, η_p^2=0.0000595; Subj: F=2.3, p=0.006, η_p^2=0.71								
10³·E2/T	33	46 (31, 67.3)	34	29.3 (19.3, 43.2)	8	141 (53, 373)	8	90.6 (33.4, 239)
Stage: F=1.2, p=0.286, η_p^2=0.0299; PMC: F=2.8, p=0.106, η_p^2=0.0675; Stage × PMC: F=0, p=0.986, η_p^2=0.00000813; Subj: F=3, p<0.001, η_p^2=0.762								
10³·E2/(A+T)	34	45.3 (35.1, 58.1)	35	37.9 (29.4, 48.5)	8	70.5 (36.4, 134)	8	67.2 (34.6, 128)
Stage: F=0.2, p=0.679, η_p^2=0.00443; PMC: F=1.3, p=0.266, η_p^2=0.0317; Stage × PMC: F=0, p=0.834, η_p^2=0.00114; Subj: F=2.1, p=0.012, η_p^2=0.69								

Table S1, continued. Changes of steroids, steroid molar ratios that may reflect activities of steroidogenic enzymes and balances between them, and of indices of MS severity after treatment with anti-MS drugs shown as re-transformed means with their 95% confidence intervals

	Follicular phase				Luteal phase			
Variable	n	Before treatment	n	After treatment	n	Before treatment	n	After treatment
**5α-Reductases (SRD5As)**								
(5α-DHP+3α/β,5α-THP)/P	38	2.64 (2.17, 3.19)	42	4.53 (3.82, 5.33)	14	0.409 (0.163, 0.766)	17	0.48 (0.234, 0.823)
Stage: F=4.4, p=0.041, η_p^2=0.0822; PMC: F=36.8, p<0.001, η_p^2=0.429; Stage × PMC: F=1.8, p=0.191, η_p^2=0.0347; Subj: F=1.2, p=0.249, η_p^2=0.589, F0<F1, F0>L0								
(3α/β,5α-THP, C)/P	44	84.7 (66.8, 107)	42	80.7 (62.8, 103)	13	4.84 (0.928, 11.7)	17	7.21 (2.74, 14.5)
Stage: F=0.2, p=0.692, η_p^2=0.00298; PMC: F=32.4, p<0.001, η_p^2=0.38; Stage × PMC: F=0.3, p=0.584, η_p^2=0.00571; Subj: F=1.1, p=0.354, η_p^2=0.552, F0>L0								
(5α,20α-THP+3α/β,5α,20α-PD)/20α-DHP	38	4.74 (4.08, 5.51)	42	4.72 (4.09, 5.46)	14	1.12 (0.799, 1.54)	17	1.4 (1.05, 1.85)
Stage: F=0.7, p=0.399, η_p^2=0.0149; PMC: F=38.3, p<0.001, η_p^2=0.444; Stage × PMC: F=0.6, p=0.445, η_p^2=0.0122; Subj: F=2, p=0.007, η_p^2=0.711, F0>L0								
(5α,20α-THP+3α/β,5α,20α-PD)/20α-DHP, C	44	158 (129, 193)	42	156 (127, 194)	13	229 (144, 380)	17	182 (122, 277)
Stage: F=0.4, p=0.527, η_p^2=0.00761; PMC: F=0.7, p=0.422, η_p^2=0.0122; Stage × PMC: F=0.3, p=0.609, η_p^2=0.00498; Subj: F=1.4, p=0.132, η_p^2=0.601								
3α,5α,17-PD, C/17-OH-P	44	1.23 (1.04, 1.46)	43	1.19 (1, 1.42)	14	0.826 (0.562, 1.2)	17	0.575 (0.403, 0.811)
Stage: F=1.7, p=0.204, η_p^2=0.0292; PMC: F=4.4, p=0.042, η_p^2=0.0734; Stage × PMC: F=0.9, p=0.346, η_p^2=0.0162; Subj: F=2.3, p<0.001, η_p^2=0.715								
103·3α,5α,17,20α-PT/17-OH-20α-DHP	42	145 (129, 162)	42	217 (193, 245)	14	141 (111, 181)	16	105 (83.6, 131)
Stage: F=0.2, p=0.631, η_p^2=0.00438; PMC: F=4.4, p=0.042, η_p^2=0.076; Stage × PMC: F=10.2, p=0.002, η_p^2=0.161; Subj: F=3.4, p<0.001, η_p^2=0.785, F0<F1								
3α,5α,17,20α-PT/17-OH-20α-DHP, C	42	52.8 (41.3, 67.8)	40	14.1 (11.1, 18)	13	9.45 (5.62, 15.9)	17	7.4 (4.72, 11.5)
Stage: F=14.3, p<0.001, η_p^2=0.215; PMC: F=10, p=0.003, η_p^2=0.161; Stage × PMC: F=5.2, p=0.026, η_p^2=0.0913; Subj: F=2.9, p<0.001, η_p^2=0.758, F0>F1, F0>L0								
(5α-DHA+3α/β,5α-THA)/A	44	0.41 (0.367, 0.458)	43	0.503 (0.448, 0.566)	14	0.458 (0.359, 0.589)	17	0.455 (0.365, 0.569)
Stage: F=1, p=0.32, η_p^2=0.018; PMC: F=0, p=0.976, η_p^2=0.0000164; Stage × PMC: F=0.9, p=0.345, η_p^2=0.0163; Subj: F=1.5, p=0.071, η_p^2=0.614								
3α/β,5α-THA, C/A	41	503 (432, 586)	40	691 (589, 811)	13	245 (173, 345)	15	225 (162, 308)
Stage: F=0.7, p=0.404, η_p^2=0.0137; PMC: F=14.3, p<0.001, η_p^2=0.218; Stage × PMC: F=1.7, p=0.202, η_p^2=0.0317; Subj: F=2.5, p<0.001, η_p^2=0.728, F0<F1, F0>L0								
(5α-DHT+3α,5α,17β-AD)/T	43	0.5 (0.446, 0.564)	43	0.552 (0.49, 0.626)	14	0.463 (0.362, 0.603)	16	0.364 (0.293, 0.456)
Stage: F=0.6, p=0.446, η_p^2=0.0108; PMC: F=1.8, p=0.186, η_p^2=0.0322; Stage × PMC: F=2.3, p=0.132, η_p^2=0.0415; Subj: F=2.2, p=0.002, η_p^2=0.702								
(5α-DHTC+3α/β,5α,17β-ADC)/T	42	145 (122, 172)	41	118 (98.3, 140)	12	135 (90.2, 199)	16	85.5 (59, 122)
Stage: F=4.7, p=0.035, η_p^2=0.086; PMC: F=0.5, p=0.502, η_p^2=0.00906; Stage × PMC: F=0.5, p=0.482, η_p^2=0.00993; Subj: F=2.8, p<0.001, η_p^2=0.762								
103·(11β-OH-3α/β,5α-THA)/11β-OH-A	43	42.4 (36.5, 48.9)	43	46.3 (39.9, 53.3)	14	54.3 (39.7, 72.2)	16	36.4 (26, 49.1)
Stage: F=1.5, p=0.22, η_p^2=0.0277; PMC: F=0, p=0.964, η_p^2=0.0000382; Stage × PMC: F=3, p=0.091, η_p^2=0.0519; Subj: F=4.5, p<0.001, η_p^2=0.828								
(11β-OH-3α/β,5α-THAC)/11β-OH-A	44	0.614 (0.505, 0.738)	43	0.823 (0.684, 0.981)	14	0.724 (0.473, 1.06)	17	0.503 (0.328, 0.734)
Stage: F=0, p=0.867, η_p^2=0.000515; PMC: F=0.3, p=0.586, η_p^2=0.00543; Stage × PMC: F=3.1, p=0.083, η_p^2=0.0538; Subj: F=2.1, p=0.003, η_p^2=0.695								

Table S1, continued. Changes of steroids, steroid molar ratios that may reflect activities of steroidogenic enzymes and balances between them, and of indices of MS severity after treatment with anti-MS drugs shown as re-transformed means with their 95% confidence intervals

**5β-Reductase (AKR1D1)**								
3α,5β-THP/P	42	0.345 (0.278, 0.427)	43	0.485 (0.393, 0.595)	14	0.0751 (0.0368, 0.134)	17	0.0936 (0.0525, 0.154)
Stage: F=1.9, p=0.171, η_p^2=0.0351; PMC: F=18.8, p<0.001, η_p^2=0.262; Stage × PMC: F=0.2, p=0.67, η_p^2=0.00346; Subj: F=1.3, p=0.143, η_p^2=0.598, F0>L0								
3α/β,5β-THPC/P	44	80.5 (62.6, 103)	42	72.2 (55.3, 93.9)	13	4.25 (0.966, 10.1)	17	5.38 (1.95, 11.1)
Stage: F=0, p=0.947, η_p^2=0.0000835; PMC: F=33.7, p<0.001, η_p^2=0.389; Stage × PMC: F=0.2, p=0.638, η_p^2=0.0042; Subj: F=0.9, p=0.629, η_p^2=0.505, F0>L0								
(3α/β,5β,20α-PD)/20α-DHP	43	1.99 (1.68, 2.35)	42	2.13 (1.79, 2.52)	14	0.459 (0.234, 0.777)	17	0.852 (0.544, 1.26)
Stage: F=3, p=0.091, η_p^2=0.052; PMC: F=14.9, p<0.001, η_p^2=0.216; Stage × PMC: F=1.3, p=0.269, η_p^2=0.0226; Subj: F=1.6, p=0.053, η_p^2=0.625, F0>L0								
(5β,20α-THP+3α/β,5β,20α-PD)/20α-DHP, C	44	41.8 (33.5, 52.4)	42	80 (62.4, 104)	13	101 (58.6, 185)	17	84.7 (53.1, 141)
Stage: F=1.3, p=0.254, η_p^2=0.0245; PMC: F=1.6, p=0.212, η_p^2=0.0292; Stage × PMC: F=2.9, p=0.095, η_p^2=0.0518; Subj: F=1.4, p=0.104, η_p^2=0.61, F0<F1, F0<L0								
103·3α,5β,17-PD/17-OH-P	39	41.7 (30.7, 56.4)	42	54.5 (40.6, 73)	14	73.5 (39.6, 136)	17	55.4 (31.6, 96.2)
Stage: F=0, p=0.972, η_p^2=0.0000258; PMC: F=0.4, p=0.511, η_p^2=0.00886; Stage × PMC: F=0.9, p=0.339, η_p^2=0.0187; Subj: F=1.3, p=0.216, η_p^2=0.6								
3α,5β,17-PDC/17-OH-P	43	7.85 (6.72, 9.21)	43	7.23 (6.19, 8.49)	14	3.61 (2.67, 4.94)	16	2.09 (1.59, 2.75)
Stage: F=6.7, p=0.012, η_p^2=0.111; PMC: F=18.9, p<0.001, η_p^2=0.259; Stage × PMC: F=3.2, p=0.081, η_p^2=0.0553; Subj: F=2.3, p=0.001, η_p^2=0.713, F0>L0								
3α,5β,17,20α-PT/17-OH-20α-DHP	44	2.58 (2.33, 2.84)	43	3.06 (2.77, 3.38)	13	1.89 (1.49, 2.38)	16	1.92 (1.56, 2.37)
Stage: F=1.1, p=0.298, η_p^2=0.02; PMC: F=5.9, p=0.019, η_p^2=0.0982; Stage × PMC: F=0.6, p=0.451, η_p^2=0.0105; Subj: F=2.3, p=0.001, η_p^2=0.711								
3α,5β,17,20α-PT/17-OH-20α-DHPC	43	194 (161, 230)	42	76.5 (58.4, 97.8)	14	97.6 (56.5, 154)	17	70.1 (40.2, 111)
Stage: F=11.1, p=0.002, η_p^2=0.17; PMC: F=1.4, p=0.235, η_p^2=0.026; Stage × PMC: F=2.4, p=0.127, η_p^2=0.0426; Subj: F=1.3, p=0.173, η_p^2=0.581, F0>F1, F0>L0								
3α,5β-THA/A	41	85.6 (74.6, 97.9)	41	103 (89.5, 117)	14	86.3 (64.1, 115)	16	71.2 (53.4, 93.4)
Stage: F=0, p=0.979, η_p^2=0.0000136; PMC: F=0.7, p=0.409, η_p^2=0.0132; Stage × PMC: F=2, p=0.161, η_p^2=0.0374; Subj: F=1.8, p=0.019, η_p^2=0.657								
3α/β,5β-THAC/A	40	40.6 (35.3, 46.7)	38	45.9 (39.7, 53)	13	24.2 (17.1, 33.4)	16	19.6 (14.1, 26.5)
Stage: F=0.1, p=0.801, η_p^2=0.00128; PMC: F=9.3, p=0.004, η_p^2=0.157; Stage × PMC: F=1.2, p=0.277, η_p^2=0.0236; Subj: F=2.4, p=0.001, η_p^2=0.719, F0>L0								
3α/β,5β,17β-AD/T, C	42	145 (122, 172)	41	118 (98.3, 140)	12	135 (90.2, 199)	16	85.5 (59, 122)
Stage: F=4.7, p=0.035, η_p^2=0.086; PMC: F=0.5, p=0.502, η_p^2=0.00906; Stage × PMC: F=0.5, p=0.482, η_p^2=0.00993; Subj: F=2.8, p<0.001, η_p^2=0.762								
11β-OH-3α,5β-THA/11β-OH-A	42	37.5 (32.3, 43.1)	42	40.3 (34.9, 46.2)	14	53.9 (40.8, 68.9)	16	31.7 (22.5, 42.5)
Stage: F=3.8, p=0.057, η_p^2=0.0669; PMC: F=0.1, p=0.727, η_p^2=0.00232; Stage × PMC: F=5.1, p=0.028, η_p^2=0.0876; Subj: F=2.4, p<0.001, η_p^2=0.721								
11β-OH-3α,5β-THAC/11β-OH-A	44	146 (117, 181)	43	240 (194, 297)	14	191 (117, 302)	17	89.2 (54.6, 140)
Stage: F=0.4, p=0.55, η_p^2=0.00652; PMC: F=1.1, p=0.306, η_p^2=0.0191; Stage × PMC: F=8.3, p=0.006, η_p^2=0.131; Subj: F=3.3, p<0.001, η_p^2=0.777, F0<F1								

Table S1, continued. Changes of steroids, steroid molar ratios that may reflect activities of steroidogenic enzymes and balances between them, and of indices of MS severity after treatment with anti-MS drugs shown as re-transformed means with their 95% confidence intervals

**Subfamily 1C aldoketoreductase type 1 (AKR1C1) vs. 17β-hydroxysteroid dehydrogenase type 2 (HSD17B2)**								
20α-DHPreg/Preg	44	1.51 (1.34, 1.69)	43	1.18 (1.04, 1.33)	14	1.52 (1.18, 1.98)	17	1.18 (0.936, 1.49)
Stage: F=6, p=0.018, η_p^2=0.0985; PMC: F=0, p=0.969, η_p^2=0.0000282; Stage × PMC: F=0, p=0.974, η_p^2=0.0000202; Subj: F=2, p=0.004, η_p^2=0.686, F0>F1								
20α-DHPreg/Preg, C	44	5.72 (5.2, 6.3)	42	4.81 (4.36, 5.31)	13	6.73 (5.4, 8.46)	17	5.42 (4.5, 6.57)
Stage: F=5, p=0.03, η_p^2=0.0858; PMC: F=0.9, p=0.354, η_p^2=0.0162; Stage × PMC: F=0, p=0.848, η_p^2=0.000696; Subj: F=3, p<0.001, η_p^2=0.767								
20α-DHP/P	43	1.6 (1.38, 1.84)	42	1.55 (1.34, 1.8)	14	0.423 (0.273, 0.623)	17	0.433 (0.295, 0.612)
Stage: F=0, p=0.973, η_p^2=0.0000212; PMC: F=26.9, p<0.001, η_p^2=0.332; Stage × PMC: F=0, p=0.875, η_p^2=0.00046; Subj: F=1.1, p=0.315, η_p^2=0.55, F0>L0								
20α-DHPC/P	43	5.85 (4.45, 7.68)	42	3.03 (2.28, 4.03)	13	0.14 (0.0149, 0.361)	16	0.201 (0.0599, 0.439)
Stage: F=0.8, p=0.369, η_p^2=0.0155; PMC: F=40.2, p<0.001, η_p^2=0.436; Stage × PMC: F=2.4, p=0.127, η_p^2=0.0443; Subj: F=1.2, p=0.296, η_p^2=0.564, F0>F1, F0>L0								
17-OH-20α-DHP/17-OH-P	43	0.568 (0.496, 0.655)	42	0.698 (0.6, 0.819)	14	0.445 (0.339, 0.598)	17	0.439 (0.344, 0.569)
Stage: F=0.6, p=0.457, η_p^2=0.0103; PMC: F=2.7, p=0.104, η_p^2=0.0483; Stage × PMC: F=0.6, p=0.439, η_p^2=0.0112; Subj: F=1.1, p=0.402, η_p^2=0.535								
5α,20α-THP/5α-DHP	38	1.53 (1.33, 1.76)	42	1.25 (1.09, 1.43)	14	1.15 (0.846, 1.53)	17	1.14 (0.873, 1.48)
Stage: F=0.8, p=0.378, η_p^2=0.0159; PMC: F=0.9, p=0.357, η_p^2=0.0173; Stage × PMC: F=0.6, p=0.445, η_p^2=0.012; Subj: F=1.1, p=0.357, η_p^2=0.568								
5α,20α-THPC/5α-DHP	39	1.05 (0.778, 1.45)	42	0.633 (0.483, 0.841)	13	0.579 (0.329, 1.07)	17	0.546 (0.332, 0.934)
Stage: F=1.3, p=0.265, η_p^2=0.0259; PMC: F=0.8, p=0.385, η_p^2=0.0158; Stage × PMC: F=0.6, p=0.448, η_p^2=0.012; Subj: F=1.1, p=0.33, η_p^2=0.582								
3α,5α,20α-PD/3α,5α-THP	42	3.23 (2.81, 3.74)	42	3.36 (2.91, 3.92)	14	1.29 (1.03, 1.62)	17	2.3 (1.83, 2.96)
Stage: F=10, p=0.003, η_p^2=0.159; PMC: F=11.8, p=0.001, η_p^2=0.182; Stage × PMC: F=6.3, p=0.015, η_p^2=0.106; Subj: F=2.4, p<0.001, η_p^2=0.722, L0<L1, F0>L0, F1<L1								
3α,5α,20α-PD/3α,5α-THP, C	44	6.58 (5.95, 7.28)	42	8.32 (7.47, 9.28)	13	7.39 (5.87, 9.34)	17	8.7 (7.09, 10.7)
Stage: F=4.6, p=0.037, η_p^2=0.0795; PMC: F=0.3, p=0.62, η_p^2=0.00467; Stage × PMC: F=0.1, p=0.731, η_p^2=0.00224; Subj: F=1.5, p=0.075, η_p^2=0.622, F0<F1								
3β,5α,20α-PD/3α,5α-THP	37	0.693 (0.489, 1.06)	41	0.379 (0.292, 0.508)	14	0.438 (0.256, 0.899)	17	0.408 (0.253, 0.75)
Stage: F=1.7, p=0.203, η_p^2=0.0343; PMC: F=0.2, p=0.702, η_p^2=0.00315; Stage × PMC: F=0.8, p=0.392, η_p^2=0.0157; Subj: F=0.9, p=0.634, η_p^2=0.529								
3β,5α,20α-PD/3α,5α-THP, C	44	7.8 (6.4, 9.74)	42	5.09 (4.28, 6.13)	13	8.06 (5.23, 14.2)	17	6.58 (4.63, 10.1)
Stage: F=3.2, p=0.078, η_p^2=0.0576; PMC: F=0.3, p=0.62, η_p^2=0.00468; Stage × PMC: F=0.4, p=0.542, η_p^2=0.00707; Subj: F=1, p=0.583, η_p^2=0.513, F0>F1								
3α,5β,20α-PD/3α,5β-THP	42	2.95 (2.36, 3.73)	43	3.08 (2.46, 3.93)	14	1.52 (1, 2.37)	17	2.01 (1.36, 3.08)
Stage: F=0.8, p=0.363, η_p^2=0.0156; PMC: F=2.7, p=0.105, η_p^2=0.0488; Stage × PMC: F=0.4, p=0.546, η_p^2=0.00691; Subj: F=1.7, p=0.029, η_p^2=0.651								
3α,5β,20α-PD/3α,5β-THP, C	43	1.89 (1.65, 2.16)	42	2.5 (2.16, 2.89)	13	3.92 (2.79, 5.73)	16	6.73 (4.65, 10.4)
Stage: F=8.1, p=0.006, η_p^2=0.135; PMC: F=12.1, p=0.001, η_p^2=0.189; Stage × PMC: F=0.3, p=0.604, η_p^2=0.0052; Subj: F=2.3, p=0.002, η_p^2=0.716, F0<F1, F0<L0								
3β,5β,20α-PD/3β,5β-THP, C	43	4.91 (4.4, 5.49)	42	6.18 (5.5, 6.97)	13	8.91 (6.76, 12.1)	16	13.7 (10.2, 19.2)
Stage: F=8.1, p=0.006, η_p^2=0.135; PMC: F=12.1, p=0.001, η_p^2=0.189; Stage × PMC: F=0.3, p=0.604, η_p^2=0.0052; Subj: F=2.3, p=0.002, η_p^2=0.716, F0<F1, F0<L0								
3α,5α,17,20α-PT/3α,5α,17-PD, C	44	21.5 (16.9, 27.6)	43	10.1 (7.94, 12.8)	14	8.13 (4.9, 13.6)	17	6.83 (4.34, 10.7)
Stage: F=5, p=0.029, η_p^2=0.084; PMC: F=3.4, p=0.071, η_p^2=0.0581; Stage × PMC: F=1.5, p=0.228, η_p^2=0.0264; Subj: F=1.9, p=0.011, η_p^2=0.666, F0>F1, F0>L0								
3α,5β,17,20α-PT/3α,5β,17-PD	39	33.9 (26.1, 44.6)	42	39.1 (30, 51.5)	14	13.2 (8.05, 21.8)	17	15.7 (10.1, 24.9)
Stage: F=0.6, p=0.451, η_p^2=0.0116; PMC: F=6.2, p=0.016, η_p^2=0.113; Stage × PMC: F=0, p=0.916, η_p^2=0.000232; Subj: F=1.4, p=0.123, η_p^2=0.625, F0>L0								
3α,5β,17,20α-PT/3α,5β,17-PD, C	42	15.5 (13.6, 17.7)	41	8.69 (7.37, 10.2)	14	9.66 (6.89, 13.2)	17	9.16 (6.75, 12.1)
Stage: F=6.5, p=0.014, η_p^2=0.11; PMC: F=1, p=0.333, η_p^2=0.0177; Stage × PMC: F=3.6, p=0.064, η_p^2=0.0634; Subj: F=4, p<0.001, η_p^2=0.81, F0>F1, F0>L0								

Table S1, continued. Changes of steroids, steroid molar ratios that may reflect activities of steroidogenic enzymes and balances between them, and of indices of MS severity after treatment with anti-MS drugs shown as re-transformed means with their 95% confidence intervals

	Follicular phase				Luteal phase			
Variable	n	Before treatment	n	After treatment	n	Before treatment	n	After treatment
**Subfamily 1C aldoketoreductase type 2 (AKR1C2) vs. vs. 17β-hydroxysteroid dehydrogenase type 2 and 6 (HSD17B2 and HSD17B6)**								
3α,5α-THP/3β,5α-THP	43	0.973 (0.846, 1.11)	43	0.587 (0.497, 0.687)	14	2.26 (1.75, 2.89)	17	1.62 (1.26, 2.04)
Stage: F=12.9, p<0.001, η_p^2=0.193; PMC: F=20.3, p<0.001, η_p^2=0.273; Stage × PMC: F=0.1, p=0.763, η_p^2=0.0017; Subj: F=1.2, p=0.209, η_p^2=0.576, F0>F1, F0<L0								
3α,5α-THP/3β,5α-THP, C	44	5.07 (4.73, 5.44)	42	5.25 (4.88, 5.67)	12	6.54 (5.49, 7.92)	16	4.73 (4.11, 5.46)
Stage: F=4.3, p=0.043, η_p^2=0.0762; PMC: F=0.4, p=0.541, η_p^2=0.00725; Stage × PMC: F=5.3, p=0.026, η_p^2=0.0922; Subj: F=2.5, p<0.001, η_p^2=0.732, L0>L1, F0<L0, F1>L1								
3α,5β-THP/3β,5β-THP, C	44	5.07 (4.73, 5.44)	42	5.25 (4.88, 5.67)	12	6.54 (5.49, 7.92)	16	4.73 (4.11, 5.46)
Stage: F=4.3, p=0.043, η_p^2=0.0762; PMC: F=0.4, p=0.541, η_p^2=0.00725; Stage × PMC: F=5.3, p=0.026, η_p^2=0.0922; Subj: F=2.5, p<0.001, η_p^2=0.732, L0>L1, F0<L0, F1>L1								
3α,5α,20α-PD/3β,5α,20α-PD	37	3.53 (2.35, 5.14)	41	5.33 (3.73, 7.46)	14	5.74 (2.69, 11.2)	17	10.3 (5.64, 18)
Stage: F=3.1, p=0.084, η_p^2=0.0621; PMC: F=1.3, p=0.257, η_p^2=0.0273; Stage × PMC: F=0.1, p=0.731, η_p^2=0.00253; Subj: F=1.3, p=0.211, η_p^2=0.608								
3α,5α,20α-PD/3β,5α,20α-PD, C	44	0.303 (0.252, 0.36)	42	0.423 (0.357, 0.495)	13	0.884 (0.67, 1.14)	17	0.903 (0.711, 1.13)
Stage: F=1.5, p=0.234, η_p^2=0.0266; PMC: F=17.6, p<0.001, η_p^2=0.25; Stage × PMC: F=0.8, p=0.383, η_p^2=0.0144; Subj: F=2.5, p<0.001, η_p^2=0.737, F0<L0								
3α,5β,20α-PD/3β,5β,20α-PD, C	44	1.81 (1.56, 2.1)	42	2.42 (2.06, 2.87)	13	2.66 (1.87, 3.91)	17	3.46 (2.48, 5)
Stage: F=3.6, p=0.062, η_p^2=0.0642; PMC: F=2.2, p=0.148, η_p^2=0.0391; Stage × PMC: F=0, p=0.871, η_p^2=0.000501; Subj: F=1.5, p=0.085, η_p^2=0.618								
3α,5α-THA/3β,5α-THA	44	1.61 (1.46, 1.79)	43	1.31 (1.2, 1.45)	13	1.34 (1.09, 1.68)	16	1.44 (1.19, 1.77)
Stage: F=0.6, p=0.462, η_p^2=0.01; PMC: F=0.1, p=0.776, η_p^2=0.00151; Stage × PMC: F=2, p=0.167, η_p^2=0.035; Subj: F=1.9, p=0.009, η_p^2=0.673, F0>F1								
3α,5α-THA/3β,5α-THA, C	40	2.98 (2.82, 3.15)	41	3.26 (3.08, 3.44)	12	3.36 (2.95, 3.85)	12	3.33 (2.92, 3.82)
Stage: F=0.6, p=0.449, η_p^2=0.0118; PMC: F=0.6, p=0.455, η_p^2=0.0114; Stage × PMC: F=0.7, p=0.404, η_p^2=0.0143; Subj: F=3.2, p<0.001, η_p^2=0.77								
3α,5β-THA/3β,5β-THA, C	42	3.22 (2.84, 3.63)	41	3.33 (2.93, 3.78)	13	4.64 (3.51, 6.16)	15	4.12 (3.19, 5.33)
Stage: F=0.1, p=0.708, η_p^2=0.00273; PMC: F=2.1, p=0.151, η_p^2=0.0393; Stage × PMC: F=0.4, p=0.546, η_p^2=0.00705; Subj: F=2.8, p<0.001, η_p^2=0.749								
3α,5α,17β-AD/3β,5α,17β-AD, C	42	0.553 (0.521, 0.587)	41	0.498 (0.468, 0.529)	13	0.53 (0.465, 0.606)	16	0.579 (0.513, 0.654)
Stage: F=0, p=0.867, η_p^2=0.000553; PMC: F=0.3, p=0.564, η_p^2=0.00656; Stage × PMC: F=2.6, p=0.114, η_p^2=0.0483; Subj: F=5.4, p<0.001, η_p^2=0.857								
3α,5β,17β-AD/3β,5β,17β-AD, C	43	16.3 (13.8, 19.5)	42	17.8 (14.9, 21.6)	13	20.2 (13.7, 32.2)	16	14.5 (10.5, 20.8)
Stage: F=0.6, p=0.441, η_p^2=0.0115; PMC: F=0, p=0.996, η_p^2=0.000000534; Stage × PMC: F=1.4, p=0.244, η_p^2=0.026; Subj: F=1, p=0.539, η_p^2=0.521								
11β-OH-3α,5α-THA/11β-OH-3β,5α-THA	42	20.2 (17.7, 23.2)	42	19.4 (17, 22.3)	14	13.7 (10.6, 18)	16	14.2 (11.2, 18.2)
Stage: F=0, p=0.982, η_p^2=0.00000937; PMC: F=3, p=0.089, η_p^2=0.0535; Stage × PMC: F=0.1, p=0.781, η_p^2=0.00147; Subj: F=1.9, p=0.011, η_p^2=0.668								
11β-OH-3α,5α-THA/11β-OH-3β,5α-THA, C	44	36.6 (31.9, 41.9)	43	33.1 (28.7, 38.1)	14	19.2 (13.5, 26.6)	17	14.6 (10.5, 20)
Stage: F=1.9, p=0.169, η_p^2=0.0341; PMC: F=10, p=0.003, η_p^2=0.153; Stage × PMC: F=0.3, p=0.615, η_p^2=0.00463; Subj: F=2.7, p<0.001, η_p^2=0.745, F0>L0								
3α,5α-THP/5α-DHP	38	1.5 (1.26, 1.77)	42	1.23 (1.03, 1.45)	14	1.31 (0.897, 1.83)	17	1.22 (0.86, 1.66)
Stage: F=0.9, p=0.338, η_p^2=0.0187; PMC: F=0.1, p=0.767, η_p^2=0.00181; Stage × PMC: F=0.2, p=0.688, η_p^2=0.00333; Subj: F=1.2, p=0.225, η_p^2=0.594								
3α,5α-THPC/5α-DHP	39	29.2 (22.8, 37.6)	42	19.1 (15.2, 24.2)	13	10.8 (6.75, 17.6)	17	9.76 (6.44, 14.9)
Stage: F=1.7, p=0.204, η_p^2=0.0334; PMC: F=5.9, p=0.019, η_p^2=0.11; Stage × PMC: F=0.4, p=0.518, η_p^2=0.00876; Subj: F=1.3, p=0.147, η_p^2=0.623, F0>L0								
3α,5α,20α-PD/5α,20α-THP, C	44	162 (132, 201)	42	224 (178, 283)	13	127 (80.4, 206)	17	122 (81.3, 185)
Stage: F=0.5, p=0.491, η_p^2=0.00899; PMC: F=1.6, p=0.208, η_p^2=0.0298; Stage × PMC: F=0.7, p=0.414, η_p^2=0.0126; Subj: F=1.4, p=0.13, η_p^2=0.602								
3α,5β,20α-PD/5β,20α-THP, C	43	118 (98.1, 141)	42	138 (114, 165)	13	94.5 (60.9, 142)	16	122 (83.8, 175)
Stage: F=1.6, p=0.211, η_p^2=0.0299; PMC: F=0.3, p=0.565, η_p^2=0.00641; Stage × PMC: F=0.1, p=0.791, η_p^2=0.00136; Subj: F=2.1, p=0.003, η_p^2=0.703								
3α,5α-THA/5α-DHA	44	2.29 (2.07, 2.53)	43	2.13 (1.92, 2.36)	14	1.79 (1.42, 2.24)	17	1.9 (1.55, 2.32)
Stage: F=0, p=0.949, η_p^2=0.0000744; PMC: F=1.3, p=0.266, η_p^2=0.0224; Stage × PMC: F=0.4, p=0.519, η_p^2=0.00761; Subj: F=1.6, p=0.034, η_p^2=0.637								
3α,5α-THAC/5α-DHA	41	4.72 (3.89, 5.69)	40	5.15 (4.23, 6.23)	13	1.55 (0.856, 2.56)	15	1.66 (0.977, 2.62)
Stage: F=0.2, p=0.681, η_p^2=0.00334; PMC: F=11.3, p=0.002, η_p^2=0.181; Stage × PMC: F=0, p=0.928, η_p^2=0.00016; Subj: F=2, p=0.007, η_p^2=0.68, F0>L0								

Table S1, continued. Changes of steroids, steroid molar ratios that may reflect activities of steroidogenic enzymes and balances between them, and of indices of MS severity after treatment with anti-MS drugs shown as re-transformed means with their 95% confidence intervals.

**Subfamily 1C aldoketoreductase type 3 (AKR1C3) vs. 17β-hydroxysteroid dehydrogenase type 2 (HSD17B2)**								
Adiol/DHEA	44	0.221 (0.203, 0.242)	43	0.182 (0.166, 0.199)	14	0.261 (0.214, 0.321)	17	0.22 (0.184, 0.262)
Stage: F=5.5, p=0.023, η_p^2=0.0908; PMC: F=1.6, p=0.214, η_p^2=0.0279; Stage × PMC: F=0, p=0.894, η_p^2=0.000328; Subj: F=1.9, p=0.008, η_p^2=0.672, F0>F1								
Adiol/DHEA, C	42	0.29 (0.264, 0.318)	42	0.273 (0.249, 0.3)	13	0.288 (0.235, 0.356)	15	0.37 (0.302, 0.46)
Stage: F=1.3, p=0.27, η_p^2=0.0238; PMC: F=0.9, p=0.345, η_p^2=0.0175; Stage × PMC: F=2.7, p=0.109, η_p^2=0.0497; Subj: F=4, p<0.001, η_p^2=0.816								
3β,7α,17β-AT/7α-OH-DHEA	43	0.335 (0.317, 0.354)	42	0.402 (0.38, 0.426)	14	0.453 (0.401, 0.514)	17	0.473 (0.424, 0.53)
Stage: F=5.5, p=0.023, η_p^2=0.0917; PMC: F=7.1, p=0.01, η_p^2=0.116; Stage × PMC: F=1.7, p=0.205, η_p^2=0.0296; Subj: F=5.8, p<0.001, η_p^2=0.861, F0<F1, F0<L0								
3β,7β,17β-AT/7β-OH-DHEA	42	0.549 (0.51, 0.592)	41	0.52 (0.482, 0.562)	14	0.698 (0.59, 0.834)	17	0.7 (0.603, 0.821)
Stage: F=0.2, p=0.691, η_p^2=0.003; PMC: F=5.1, p=0.028, η_p^2=0.0882; Stage × PMC: F=0.2, p=0.694, η_p^2=0.00294; Subj: F=3.7, p<0.001, ηp2=0.8								
T/A	42	0.191 (0.171, 0.212)	41	0.227 (0.204, 0.253)	14	0.203 (0.16, 0.254)	17	0.254 (0.208, 0.306)
Stage: F=4.8, p=0.033, η_p^2=0.083; PMC: F=0.3, p=0.59, η_p^2=0.00551; Stage × PMC: F=0.1, p=0.807, η_p^2=0.00114; Subj: F=1.7, p=0.021, η_p^2=0.652								
5α-DHT/5α-DHA	44	0.739 (0.612, 0.878)	42	0.79 (0.651, 0.943)	13	0.85 (0.547, 1.22)	17	0.799 (0.545, 1.1)
Stage: F=0, p=0.993, η_p^2=0.00000135; PMC: F=0.1, p=0.794, η_p^2=0.00127; Stage × PMC: F=0.1, p=0.715, η_p^2=0.00249; Subj: F=1.3, p=0.175, η_p^2=0.58								
5α-DHTC/5α-DHA	42	4.99 (3.91, 6.39)	42	3.85 (3.01, 4.93)	13	3.06 (1.81, 5.21)	16	1.1 (0.633, 1.83)
Stage: F=8.9, p=0.004, η_p^2=0.149; PMC: F=4.9, p=0.032, η_p^2=0.0875; Stage × PMC: F=2.5, p=0.12, η_p^2=0.0468; Subj: F=1.9, p=0.013, η_p^2=0.679								
3α,5α,17β-AD/3α,5α-THA	44	0.163 (0.148, 0.18)	43	0.199 (0.18, 0.221)	14	0.208 (0.167, 0.261)	17	0.222 (0.182, 0.274)
Stage: F=2.3, p=0.132, η_p^2=0.0409; PMC: F=1.2, p=0.271, η_p^2=0.022; Stage × PMC: F=0.5, p=0.493, η_p^2=0.00857; Subj: F=2.5, p<0.001, η_p^2=0.724, F0<F1								
3α,5α,17β-AD/3α,5α-THA, C	41	29.4 (27.8, 31.1)	38	23.5 (22.2, 24.9)	11	22.4 (19.6, 25.5)	15	22 (19.7, 24.6)
Stage: F=5.2, p=0.028, η_p^2=0.0952; PMC: F=3.6, p=0.064, η_p^2=0.0682; Stage × PMC: F=3.1, p=0.084, η_p^2=0.0599; Subj: F=6.5, p<0.001, η_p^2=0.874, F0>F1, F0>L0								
3β,5α,17β-AD/3β,5α-THA, C	42	163 (151, 176)	41	156 (144, 168)	13	159 (134, 189)	15	139 (118, 162)
Stage: F=1.7, p=0.193, η_p^2=0.0324; PMC: F=0.3, p=0.579, η_p^2=0.00597; Stage × PMC: F=0.4, p=0.544, η_p^2=0.00713; Subj: F=5.5, p<0.001, η_p^2=0.853								
3α,5β,17β-AD/3α,5β-THA, C	43	78.3 (71, 86.4)	41	65.7 (59.2, 72.8)	13	93 (74.4, 116)	16	66.4 (54.3, 81)
Stage: F=8.1, p=0.006, η_p^2=0.132; PMC: F=0.3, p=0.561, η_p^2=0.00643; Stage × PMC: F=0.6, p=0.437, η_p^2=0.0114; Subj: F=2.6, p<0.001, η_p^2=0.734								
3β,5β,17β-AD/3β,5β-THA, C	41	15.1 (12.9, 18.2)	39	12.2 (10.5, 14.3)	12	13.7 (9.9, 21.6)	15	10.6 (8.31, 14.3)
Stage: F=3.1, p=0.084, η_p^2=0.0585; PMC: F=0.2, p=0.637, η_p^2=0.0045; Stage × PMC: F=0, p=0.837, η_p^2=0.000859; Subj: F=1.3, p=0.207, η_p^2=0.572								
**Indices of MS severity**								
Expanded Disability Status Scale (EDSS)	42	1.8 (1.71, 1.89)	42	1.79 (1.7, 1.88)	14	2.31 (2.11, 2.51)	16	2.2 (2.02, 2.39)
Stage: F=0.5, p=0.469, η_p^2=0.00995; PMC: F=10, p=0.003, η_p^2=0.158; Stage × PMC: F=0.3, p=0.595, η_p^2=0.00538; Subj: F=15.4, p<0.001, η_p^2=0.943, F0<L0								
Timed 25-Foot Walk (T25FWT)	30	5.1 (4.98, 5.23)	32	4.97 (4.86, 5.09)	11	5.04 (4.81, 5.31)	11	4.9 (4.68, 5.14)
Stage: F=1.8, p=0.194, η_p^2=0.044; PMC: F=0.1, p=0.713, η_p^2=0.00361; Stage × PMC: F=0, p=0.923, η_p^2=0.00025; Subj: F=30.9, p<0.001, η_p^2=0.972								
Nine Hole Peg Test for right hand (HPT9_P)	29	19 (18.6, 19.5)	31	18.5 (18.1, 18.9)	11	19.4 (18.6, 20.3)	11	18.1 (17.3, 19)
Stage: F=6.2, p=0.018, η_p^2=0.143; PMC: F=0, p=0.999, η_p^2=0.0000000152; Stage × PMC: F=1, p=0.332, η_p^2=0.0255; Subj: F=4.5, p<0.001, η_p^2=0.833								
Nine Hole Peg Test for left hand (HPT9_L)	30	20.4 (20, 20.9)	32	19.2 (18.8, 19.6)	11	20.8 (19.9, 21.9)	11	19.5 (18.7, 20.4)
Stage: F=11.9, p=0.001, η_p^2=0.238; PMC: F=0.3, p=0.613, η_p^2=0.00681; Stage × PMC: F=0, p=0.99, η_p^2=0.00000415; Subj: F=4.9, p<0.001, η_p^2=0.844, F0>F1								

PMC = phase of menstrual cycle, Subj = subject, F0= follicular phase before treatment, F1 = follicular phase after treatment, L0 = luteal phase before treatment, L1 = luteal phase after treatment, F = F-statistic, p = p-value, n_p^2 = effect size, n=number of patients
