# Supplementary material for: Effect of Treatment on Steroidome in Women with Multiple Sclerosis
Source: Int J Mol Sci. 2025 Feb 20;26(5):1835. doi: 10.3390/ijms26051835 (PMC11899614; doi:10.3390/ijms26051835)
Supplement: Supplementary file 1 [file ijms-26-01835-s001.zip › Effect of treatment on steroidome in women with multiple sclerosis v021, Table S2.pdf]

Table S2. Effects of treatment with individual anti-MS drugs and/or their groups on steroidome as evaluated by orthogonal projections to latent structures (OPLS) and ordinary multiple regression (OMR)

	**GA** follicular phase			**IFNβ-1a** follicular phase			**IFNβ-1a** luteal phase			**S1PRI** follicular phase			**Ofatumumab** follicular phase			**Ocrelizumab** follicular phase			**Ocrelizumab** luteal phase		
**Variable**	Component loading, OPLS	OPLS, p-value	OMR, p-value	Component loading, OPLS	OPLS, p-value	OMR, p-value	Component loading, OPLS	OPLS, p-value	OMR, p-value	Component loading, OPLS	OPLS, p-value	OMR, p-value	Component loading, OPLS	OPLS, p-value	OMR, p-value	Component loading, OPLS	OPLS, p-value	OMR, p-value	Component loading, OPLS	OPLS, p-value	OMR, p-value
**Steroids**																					
Pregnenolone (Preg)							0.870	**	*												
Pregnenolone sulfate (PregC)																			-0.820	**	*
20α-Dihydropregnenolone (20α-DHPreg)				-0.282	*																
20α-Dihydropregnenolone sulfate (20α-DHPregC)				-0.580	*																
17-Hydroxypregnenolone (17-OH-Preg)							0.759	**													
17-Hydroxypregnenolone sulfate (17-OH-PregC)							0.885	**	**							-0.701	**	*			
16α-Hydroxypregnenolone (16α-OH-Preg)																					
Dehydroepiandrosterone (DHEA)																					
DHEA sulfate (DHEAC)																			-0.533	*	
7α-Hydroxy-DHEA (7α-OH-DHEA)													-0.682	**	**						
7β-Hydroxy-DHEA (7β-OH-DHEA)																					
Androstenediol (Adiol)																					
Androstenediol sulfate (AdiolC)																					
5-Androstene-3β,7α,17β-triol (3β,7α,17β-AT)													-0.593	**	**						
5-Androstene-3β,7β,17β-triol (3β,7β,17β-AT)													-0.633	**	**	-0.634	**	**			
5-Androstene-3β,16α,17β-triol (3β,16α,17β-AT)							0.819	**					-0.674	**	**						
5-Androstene-3β,16α,17β-triol sulfate (3β,16α,17β-ATC)							0.581	**	*										-0.570	**	**
Progesterone (P)																			0.079		**
20α-Dihydroprogesterone (20α-DHP)																-0.455	**	*			
Conjugated 20α-dihydroprogesterone (20α-DHPC)							0.733	**	*	-0.358	*		-0.617	**	**	-0.581	**	**			
17-Hydroxyprogesterone (17-OH-P)													-0.652	**	**	-0.751	**	*			
17,20α-Dihydroxy-4-pregnene-3-one (17-OH-20α-DHP)																					
16α-Hydroxyprogesterone (16α-OH-P)							0.695	*					-0.738	**	**	-0.621	**	*			
Androstenedione (A)													-0.668	**	*						
Testosterone (T)							0.632	**	*												
5α-Dihydrotestosterone (5α-DHT)							0.648	**											-0.705	**	*
Conjugated 5α-dihydrotestosterone (5α-DHTC)													-0.461	**	*						
Estrone sulfate (E1C)													-0.504	**	**	-0.564	**	**			
Estradiol (E2)																-0.527	**	*			
5α-Dihydroprogesterone (5α-DHP)																			-0.039		*
Allopregnanolone (3α,5α-THP)																					
Allopregnanolone sulfate (3α,5α-THPC)				-0.405	**					-0.410	**	**	-0.577	**	*						
Isopregnanolone (3β,5α-THP)																			0.079		*
Isopregnanolone sulfate (3β,5α-THPC)							0.768	**	*	-0.280	**										

Table S2. Effects of treatment with individual anti-MS drugs and/or their groups on steroidome as evaluated by orthogonal projections to latent structures (OPLS) and ordinary multiple regression (OMR)

	**GA** follicular phase			**IFNβ-1a** follicular phase			**IFNβ-1a** luteal phase			**S1PRI** follicular phase			**Ofatumumab** follicular phase			**Ocrelizumab** follicular phase			**Ocrelizumab** luteal phase		
**Variable**	Component loading, OPLS	OPLS, p-value	OMR, p-value	Component loading, OPLS	OPLS, p-value	OMR, p-value	Component loading, OPLS	OPLS, p-value	OMR, p-value	Component loading, OPLS	OPLS, p-value	OMR, p-value	Component loading, OPLS	OPLS, p-value	OMR, p-value	Component loading, OPLS	OPLS, p-value	OMR, p-value	Component loading, OPLS	OPLS, p-value	OMR, p-value
**Steroids**																					
Pregnanolone (3α,5β-THP)																					
Conjugated pregnanolone (3α,5β-THPC)																					
Conjugated epipregnanolone (3β,5β-THPC)										-0.286	*										
17-Hydroxyallopregnanolone sulfate (3α,5α,17-PDC)				-0.423	**					-0.325	*	*	-0.802	**	**	-0.813	**	*			
17-Hydroxypregnanolone (3α,5β,17-PD)							0.463	*								-0.639	**	*	-0.537	**	*
Conjugated 17-hydroxypregnanolone (3α,5β,17-PDC)				-0.409	**	**							-0.728	**	**	-0.791	**	**			
5α,20α-Tetrahydroprogesterone (5α,20α-THP)							0.738	**	*												
Conjugated 5α,20α-tetrahydroprogesterone (5α,20α-THPC)																-0.533	**				
5α-Pregnane-3α,20α-diol (3α,5α,20α-PD)							0.593	**	*												
Conjugated 5α-pregnane-3α,20α-diol (3α,5α,20α-PDC)																					
5α-Pregnane-3β,20α-diol (3β,5α,20α-PD)										-0.296	**		-0.537	**	**	-0.536	**	*			
Conjugated 5α-pregnane-3β,20α-diol (3β,5α,20α-PDC)										-0.304	*		-0.427	**	*						
Conjugated 5β,20α-tetrahydroprogesterone (5β,20α-THPC)																					
5β-Pregnane-3α,20α-diol (3α,5β,20α-PD)																					
Conjugated 5β-pregnane-3α,20α-diol (3α,5β,20α-PDC)										0.466	*	*									
5β-Pregnane-3β,20α-diol (3β,5β,20α-PD)							0.494	**	*												
Conjugated 5β-pregnane-3β,20α-diol (3β,5β,20α-PD)																					
5α-Pregnane-3α,17,20α-triol (3α,5α,17,20α-PT)																			-0.666	**	*
Conjugated 5α-pregnane-3α,17,20α-triol (3α,5α,17,20α-PTC)										-0.630	**		-0.732	**	**	-0.569	**	**	-0.641	**	*
5β-Pregnane-3α,17,20α-triol (3β,5α,17,20α-PT)																			-0.757	**	*
Conjugated 5β-pregnane-3α,17,20α-triol (3α,5β,17,20α-PTC)							0.690	**		-0.499	**	**				-0.762	**	**	-0.812	**	*
5α-Androstane-3,17-dione (5α-DHA)				0.355	*	**															
Androsterone (3α,5α-THA)																					
Androsterone sulfate (3α,5α-THAC)																					
Epiandrosterone (3β,5α-THA)																					
Epiandrosterone sulfate (3β,5α-THAC)																					
Etiocholanolone (3α,5β-THA)																					
Epietiocholanolone (3α,5β-THAC)																			-0.635	**	*
Epietiocholanolone sulfate (3β,5β-THAC)																			-0.641	**	

Table S2. Effects of treatment with individual anti-MS drugs and/or their groups on steroidome as evaluated by orthogonal projections to latent structures (OPLS) and ordinary multiple regression (OMR)

	**GA** follicular phase			**IFNβ-1a** follicular phase			**IFNβ-1a** luteal phase			**S1PRI** follicular phase			**Ofatumumab** follicular phase			**Ocrelizumab** follicular phase			**Ocrelizumab** luteal phase		
**Variable**	Component loading, OPLS	OPLS, p-value	OMR, p-value	Component loading, OPLS	OPLS, p-value	OMR, p-value	Component loading, OPLS	OPLS, p-value	OMR, p-value	Component loading, OPLS	OPLS, p-value	OMR, p-value	Component loading, OPLS	OPLS, p-value	OMR, p-value	Component loading, OPLS	OPLS, p-value	OMR, p-value	Component loading, OPLS	OPLS, p-value	OMR, p-value
**Steroids**																					
5α-Androstane-3α,17β-diol (3α,5α,17β-AD)																			-0.611	**	*
Conjugated 5α-androstane-3α,17β-diol (3α,5α,17β-ADC)																			-0.623	**	
Conjugated 5α-androstane-3β,17β-diol (3β,5α,17β-ADC)				0.501	*	**													-0.754	**	*
Conjugated 5α-androstane-3α,17β-diol (3α,5β,17β-ADC)										-0.266	*					-0.479	**	*	-0.772	**	*
Conjugated 5α-androstane-3β,17β-diol (3β,5β,17β-ADC)							0.729	**	**												
Cortisol (F)							0.740	**	*	-0.274	**					0.227	*				
Cortisol, RIA (F, RIA)										-0.348	**	*									
Cortisone (E)																			-0.350		**
Corticosterone (B)																			-0.632	*	
11β-Hydroxyandrostenedione (11β-OH-A)				-0.344		*	0.602	**											-0.669	*	*
11β-Hydroxyandrosterone (11β-OH-3α,5α-THA)																					
11β-Hydroxyandrosterone sulfate (11β-OH-3α,5α-THAC)													-0.740	**	*				-0.807	**	**
11β-Hydroxyepiandrosterone (11β-OH-3β,5α-THA)																					
11β-Hydroxyepiandrosterone sulfate (11β-OH-3β,5α-THAC)							0.657	**	*												
11β-Hydroxyetiocholanolone (11β-OH-3α,5β-THA)							0.629	**	*										-0.568	*	
11β-Hydroxyetiocholanolone sulfate (11β-OH-3α,5β-THAC)							0.630	**	*												
Explained variability, OPLS				0.693	**		0.581	**		0.782	**		0.549	**		0.486	*		0.630	**	
**C17-hydroxylase, C17,20-lyase (CYP17A1), hydroxylase + lyase steps**																					
Preg/DHEA																					
Preg/DHEA, C																					
20α-DHPreg/DHEA				0.546	**	*				0.407	*	*									
20α-DHPreg/DHEA, C																					
A/P																			-0.820	**	**
A/20α-DHP																			-0.841	**	*
5α-DHA/5α-DHP																					
5α-DHA/5α,20α-THP																			-0.757	**	*
3α,5α-THA/3α,5α-THP																					
3α,5α-THA/3α,5α-THP, C				0.493	**					0.512	**	*							-0.731	**	*
3α,5α-THA/3α,5α,20α-PD																			-0.910	**	*
3α,5α-THA/3α,5α,20α-PD, C				0.376	**					0.322	*								-0.637	**	*
3β,5α-THA/3β,5α-THP				-0.597	**	**															
3β,5α-THA/3β,5α-THP, C				0.248	**	*				0.423	**										
3β,5α-THA/3β,5α,20α-PD										0.362	**										
3β,5α-THA/3β,5α,20α-PD, C										0.514	**										

Table S2. Effects of treatment with individual anti-MS drugs and/or their groups on steroidome as evaluated by orthogonal projections to latent structures (OPLS) and ordinary multiple regression (OMR)

	**GA** follicular phase			**IFNβ-1a** follicular phase			**IFNβ-1a** luteal phase			**S1PRI** follicular phase			**Ofatumumab** follicular phase			**Ocrelizumab** follicular phase			**Ocrelizumab** luteal phase		
**Variable**	Component loading, OPLS	OPLS, p-value	OMR, p-value	Component loading, OPLS	OPLS, p-value	OMR, p-value	Component loading, OPLS	OPLS, p-value	OMR, p-value	Component loading, OPLS	OPLS, p-value	OMR, p-value	Component loading, OPLS	OPLS, p-value	OMR, p-value	Component loading, OPLS	OPLS, p-value	OMR, p-value	Component loading, OPLS	OPLS, p-value	OMR, p-value
**C17-hydroxylase, C17,20-lyase (CYP17A1), hydroxylase + lyase steps**																					
3α,5β-THA/3α,5β-THP																			-0.701	*	
3α,5β-THA/3α,5β-THP, C																					
3α,5β-THA/3α,5β,20α-PD																			-0.780	**	*
3α,5β-THA/3α,5β,20α-PD, C										-0.659	*	**									
3β,5β-THA/3β,5β-THP, C				0.432	**	*															
3β,5β-THA/3β,5β,20α-PD, C																					
11β-OH-A/B																					
Explained variability, OPLS				0.693	**					0.684	*								0.517	*	
**C17-hydroxylase, C17,20-lyase (CYP17A1), hydroxylase step**																					
17-OH-Preg/Preg																-0.563	**	*			
17-OH-Preg/Preg, C							0.809	**	**				-0.615	**	**	-0.737	**	*			
17-OH-P/P										-0.289	*										
17-OH-20α-DHP/20α-DHP																			-0.804	**	
3α,5α,17-PD/3α,5α-THP, C													-0.725	**	**				-0.630	*	*
3α,5β,17-PD/3α,5β-THP				-0.687	**	*										-0.594	**	**	-0.640	**	
3α,5β,17-PD/3α,5β-THP, C													-0.702	**	*	-0.617	**	**	-0.793	**	
3α,5α,17,20α-PT/3α,5α,20α-PD							-0.792	**											-0.821	**	
3α,5α,17,20α-PT/3α,5α,20α-PD, C				-0.722	**	*				-0.819	**	*	-0.762	**	**	-0.763	**	*			
3α,5β,17,20α-PT/3α,5β,20α-PD																			-0.904	**	
3α,5β,17,20α-PT/3α,5β,20α-PD, C				-0.792	**	*				-0.918	**	*	-0.602	*	*	-0.742	**	*	-0.796	**	*
F/B													0.631	*							
F(RIA)/B													0.732	**	*						
Explained variability, OPLS				0.564	**		0.624	**		0.582	**		0.622	**		0.499	*		0.506	**	
**C17-hydroxylase, C17,20-lyase (CYP17A1), lyase step**																					
DHEA/17-OH-Preg	0.872	**	*	0.625	**	*										0.816	**	*			
DHEA/17-OH-Preg, C				0.693	**											0.773	**	*			
A/17-OH-P	0.856	**	*													0.705	**	*			
A/17-OH-20α-DHP																					
3α,5α-THA/3α,5α,17-PD, C				0.813	**	*							0.428	**							
3α,5β-THA/3α,5β,17-PD																0.641	**	*			
3α,5β-THA/3α,5β,17-PD, C													0.564	**		0.711	**				
3α,5α-THA/3α,5α,17,20α-PT																					
3α,5α-THA/3α,5α,17,20α-PT, C				0.613	**	*							0.730	**	*	0.679	**				
3α,5β-THA/3α,5β,17,20α-PT													-0.368	*	**						

Table S2. Effects of treatment with individual anti-MS drugs and/or their groups on steroidome as evaluated by orthogonal projections to latent structures (OPLS) and ordinary multiple regression (OMR)

	**GA** follicular phase			**IFNβ-1a** follicular phase			**IFNβ-1a** luteal phase			**S1PRI** follicular phase			**Ofatumumab** follicular phase			**Ocrelizumab** follicular phase			**Ocrelizumab** luteal phase		
**Variable**	Component loading, OPLS	OPLS, p-value	OMR, p-value	Component loading, OPLS	OPLS, p-value	OMR, p-value	Component loading, OPLS	OPLS, p-value	OMR, p-value	Component loading, OPLS	OPLS, p-value	OMR, p-value	Component loading, OPLS	OPLS, p-value	OMR, p-value	Component loading, OPLS	OPLS, p-value	OMR, p-value	Component loading, OPLS	OPLS, p-value	OMR, p-value
**C17-hydroxylase, C17,20-lyase (CYP17A1), lyase step**																					
3α,5β-THA/3α,5β,17,20α-PT, C				0.549	**	*							0.710	**	**	0.535	**				
11β-OH-A/F																					
11β-OH-A/F(RIA)																					
Explained variability, OPLS				0.540	**								0.669	**							
**3β-Hydroxysteroid dehydrogenases (HSD3Bs)**																					
P/Preg																					
P/Preg, C																			0.935	**	
20α-DHP/20α-DHPreg																					
20α-DHP/20α-DHPreg, C													-0.465	*							
17-OH-P/17-OH-Preg													-0.693	**	**						
17-OH-P/17-OH-Preg, C																			0.936	**	*
16α-OH-P/16α-OH-Preg																					
A/DHEA													-0.869	**	**						
A/DHEA, C													-0.647	**	*						
T/Adiol																					
T/Adiol, C																					
Explained variability, OPLS													0.557	**					0.431	*	
**Conjugated/unconjugated steroids (sulfotransferase 2A1/steroid sulfatase, SULT2A1/STS)**																					
Preg, C/U																			-0.752	**	*
20α-DHPreg, C/U	-0.659	**																	-0.705	**	*
17-OH-Preg, C/U										-0.359	*								-0.504	*	*
DHEA, C/U	-0.495	**																			
Adiol, C/U																					
3β,16α,17β-AT, C/U	-0.659	**	*																		
20α-DHP, C/U										-0.322	*		-0.309	*							
3α,5α-THP, C/U													-0.332	**							
3β,5α-THP, C/U													-0.310	*							
3α,5β-THP, C/U				-0.661	**	*										-0.450	**	**			
3α,5β,17-PD, C/U																					
5α,20α-THP, C/U	-0.810	**	*													-0.590			-0.645	*	**
3α,5α,20α-PD, C/U																					
3β,5α,20α-PD, C/U	-0.372	*																			
3α,5β,20α-PD, C/U	-0.685	**								0.350	*	**	0.360	**	**						
3β,5β,20α-PD, C/U																					

Table S2. Effects of treatment with individual anti-MS drugs and/or their groups on steroidome as evaluated by orthogonal projections to latent structures (OPLS) and ordinary multiple regression (OMR)

Variable	**GA** follicular phase			**IFNβ-1a** follicular phase			**IFNβ-1a** luteal phase			**S1PRI** follicular phase			**Ofatumumab** follicular phase			**Ocrelizumab** follicular phase			**Ocrelizumab** luteal phase		
	Component loading, OPLS	OPLS, p-value	OMR, p-value	Component loading, OPLS	OPLS, p-value	OMR, p-value	Component loading, OPLS	OPLS, p-value	OMR, p-value	Component loading, OPLS	OPLS, p-value	OMR, p-value	Component loading, OPLS	OPLS, p-value	OMR, p-value	Component loading, OPLS	OPLS, p-value	OMR, p-value	Component loading, OPLS	OPLS, p-value	OMR, p-value
**Conjugated/unconjugated steroids (sulfotransferase 2A1/steroid sulfatase, SULT2A1/STS)**																					
3α,5α,17,20α-PT, C/U				-0.674	**	*				-0.563	**	**	-0.764	**	*	-0.808	**	*			
3α,5β,17,20α-PT, C/U				-0.745	**	**				-0.805	**	**	-0.892	**	**	-0.797	*	**	-0.676		*
3α,5α-THA, C/U				0.275		**															
3β,5α-THA, C/U																					
3α,5β-THA, C/U																					
5α-DHT, C/U																					
3α,5α,17β-AD, C/U																-0.424	*	*			
11β-OH-3α,5α-THA, C/U																			-0.457		*
11β-OH-3β,5α-THA, C/U																					
11β-OH-3α,5β-THA, C/U																					
Explained variability, OPLS	0.428	*		0.556	*					0.728	**		0.757	**		0.506	*				
**7α/β- and 16α-hydroxylating enzymes (CYP7B1, CYP3A4, CYP3A7)**																					
7α-OH-DHEA/DHEA										-0.838	**	**	-0.783	**	**	-0.960	**	**	-0.932	**	*
3β,7α,17β-AT/Adiol										-0.787	**		-0.556	*	*						
7β-OH-DHEA/DHEA													-0.310	*	*	-0.601	**		-0.725	**	*
3β,7β,17β-AT/Adiol										0.729	**	*	-0.534	**		-0.639	**				
16α-OH-Preg/Preg													-0.567	**		-0.392			-0.720	**	*
3β,16α,17β-AT/Adiol													-0.616	*	**						
3β,16α,17β-AT/Adiol, C																					
16α-OH-P/P													-0.198						-0.642	**	*
Explained variability, OPLS										0.416	*		0.760	**		0.522	*		0.587	**	
**11β-Hydroxysteroid dehydrogenase type1 (HSD11B1)**																					
7β-OH-DHEA/7α-OH-DHEA													0.641	**	**						
3β,7β,17β-AT/3β,7α,17β-AT													-0.199		*	-0.563	*	**			
F/E													-0.826	**	**	0.685	**	**			
F(RIA)/E																					
Explained variability, OPLS													0.553	**		0.436	*				
**5α-Reductases (SRD5As)**																					
(5α-DHP+3α/β,5α-THP)/P										-0.489	*	*	0.502	*	*	0.767	**	**			
(3α/β,5α-THP, C)/P										-0.685	**					0.478	*	*			
(5α,20α-THP+3α/β,5α,20α-PD)/20α-DHP																					
(5α,20α-THP+3α/β,5α,20α-PD)/20α-DHP, C																					
3α,5α,17-PD, C/17-OH-P										-0.802	**	*									
3α,5α,17,20α-PT/17-OH-20α-DHP										-0.461	*	*				0.603	**	**			

Table S2. Effects of treatment with individual anti-MS drugs and/or their groups on steroidome as evaluated by orthogonal projections to latent structures (OPLS) and ordinary multiple regression (OMR)

Variable	**GA** follicular phase			**IFNβ-1a** follicular phase			**IFNβ-1a** luteal phase			**S1PRI** follicular phase			**Ofatumumab** follicular phase			**Ocrelizumab** follicular phase			**Ocrelizumab** luteal phase		
	Component loading, OPLS	OPLS, p-value	OMR, p-value	Component loading, OPLS	OPLS, p-value	OMR, p-value	Component loading, OPLS	OPLS, p-value	OMR, p-value	Component loading, OPLS	OPLS, p-value	OMR, p-value	Component loading, OPLS	OPLS, p-value	OMR, p-value	Component loading, OPLS	OPLS, p-value	OMR, p-value	Component loading, OPLS	OPLS, p-value	OMR, p-value
**5α-Reductases (SRD5As)**																					
(5α-DHA+3α/β,5α-THA)/A													0.806	**	**						
3α/β,5α-THA, C/A													0.656	**	*						
(5α-DHT+3α,5α,17β-AD)/T																					
(5α-DHTC+3α/β,5α,17β-ADC)/T																					
11β-OH-3α,5β-THA/11β-OH-A																					
11β-OH-3α,5β-THAC/11β-OH-A																					
Explained variability, OPLS										0.495	**		0.590	**		0.464	*				
**5β-Reductase (AKR1D1)**																					
3α,5β-THP/P							0.787	**	*	-0.616	**	*									
3α/β,5β-THPC/P																			-0.473		**
(3α/β,5β,20α-PD)/20α-DHP							0.787	**	*												
(5β,20α-THP+3α/β,5β,20α-PD)/20α-DHP, C							-0.410			0.537	*	*	0.452	**							
3α,5β,17-PD/17-OH-P							0.660	**	*										-0.694	**	
3α,5β,17-PDC/17-OH-P										-0.800	**	*							-0.795	**	*
3α,5β,17,20α-PT/17-OH-20α-DHP							0.773	*	**				0.598	**	**				-0.813		**
3α,5β,17,20α-PTC/17-OH-20α-DHP							0.755	**		-0.695	**	*	-0.580	**	**				-0.787	**	*
3α,5β-THA/A													0.567	*							
3α/β,5β-THAC/A													0.554	*					-0.570		*
3α/β,5β,17β-ADC/T										-0.458	**	*							-0.659	*	*
11β-OH-3α,5β-THA/11β-OH-A																					
11β-OH-3α,5β-THAC/11β-OH-A																					
Explained variability, OPLS							0.558	*		0.606	**		0.732	**					0.698	*	
**Subfamily 1C aldoketoreductase type 1 (AKR1C1) vs. 17β-hydroxysteroid dehydrogenase type 2 (HSD17B2)**																					
20α-DHPreg/Preg	-0.590	**	*							-0.395	**										
20α-DHPreg/Preg, C							-0.714	**	**												
20α-DHP/P										-0.498	**										
20α-DHPC/P							0.660	**	**												
17-OH-20α-DHP/17-OH-P													0.464	*	*						
5α,20α-THP/5α-DHP	-0.644	**	*																-0.880	**	**
5α,20α-THPC/5α-DHP	-0.654	**	*	-0.364	*																
3α,5α,20α-PD/3α,5α-THP							0.783	**	*				0.079								
3α,5α,20α-PD/3α,5α-THP, C				0.586	**	*				0.536	**	*									
3β,5α,20α-PD/3β,5α-THP				-0.300	*					-0.125	**		-0.395	*							
3β,5α,20α-PD/3β,5α-THP, C	-0.516	**		0.441	**					-0.114			-0.259	**							

Table S2. Effects of treatment with individual anti-MS drugs and/or their groups on steroidome as evaluated by orthogonal projections to latent structures (OPLS) and ordinary multiple regression (OMR)

Variable	**GA** follicular phase			**IFNβ-1a** follicular phase			**IFNβ-1a** luteal phase			**S1PRI** follicular phase			**Ofatumumab** follicular phase			**Ocrelizumab** follicular phase			**Ocrelizumab** luteal phase		
	Component loading, OPLS	OPLS, p-value	OMR, p-value	Component loading, OPLS	OPLS, p-value	OMR, p-value	Component loading, OPLS	OPLS, p-value	OMR, p-value	Component loading, OPLS	OPLS, p-value	OMR, p-value	Component loading, OPLS	OPLS, p-value	OMR, p-value	Component loading, OPLS	OPLS, p-value	OMR, p-value	Component loading, OPLS	OPLS, p-value	OMR, p-value
**Subfamily 1C aldoketoreductase type 1 (AKR1C1) vs. 17β-hydroxysteroid dehydrogenase type 2 (HSD17B2)**																					
3α,5β,20α-PD/3α,5β-THP																					
3α,5β,20α-PD/3α,5β-THP, C										0.608	**	*									
3β,5β,20α-PD/3β,5β-THP, C	-0.532	**					-0.563	**	*												
3α,5α,17,20α-PT/3α,5α,17-PD, C										-0.611	**	*	-0.848	**	**						
3α,5β,17,20α-PT/3α,5β,17-PD							-0.278														
3α,5β,17,20α-PT/3α,5β,17-PD, C	-0.382	*	*	-0.616	*	**	0.514	*		-0.457		*							-0.748	**	*
Explained variability, OPLS	0.552	*		0.542	**		0.655	*		0.682	**		0.624	**					0.683	**	
**Subfamily 1C aldoketoreductase type 2 (AKR1C2) vs. vs. 17β-hydroxysteroid dehydrogenase type 2 and 6 (HSD17B2 and HSD17B6)**																					
3α,5α-THP/3β,5α-THP	-0.590	*								-0.715	**	*	-0.756	**	*	-0.770	**	*			
3α,5α-THP/3β,5α-THP, C	-0.547	**	*				-0.578	*		-0.669	*	*	-0.587	*	*	-0.793	**	*			
3α,5β-THP/3β,5β-THP, C							-0.551	*	*												
3α,5α,20α-PD/3β,5α,20α-PD													0.630	*	*						
3α,5α,20α-PD/3β,5α,20α-PD, C	0.631	**								0.527	*					0.642	**	**			
3α,5β,20α-PD/3β,5β,20α-PD				0.284		*															
3α,5β,20α-PD/3β,5β,20α-PD, C	0.684	**								0.837	**	**									
3α,5α-THA/3β,5α-THA	-0.623	**	*																		
3α,5α-THA/3β,5α-THA, C				0.560	*	*															
3α,5β-THA/3β,5β-THA, C													-0.303	*							
3α,5α,17β-AD/3β,5α,17β-AD, C				-0.537	*	**															
3α,5β,17β-AD/3β,5β,17β-AD, C							-0.751	**	**							0.458	*	*			
11β-OH-3α,5α-THA/11β-OH-3β,5α-THA				0.561	**	*															
11β-OH-3α,5α-THA/11β-OH-3β,5α-THA, C																0.340	**	*			
3α,5α-THP/5α-DHP																					
3α,5α-THPC/5α-DHP				-0.590	**	**															
3α,5α,20α-PD/5α,20α-THP							0.624	*	**				0.571	**	**	0.492	**				
3α,5α,20α-PD/5α,20α-THP, C	0.633	**	*				-0.426														
3α,5β,20α-PD/5β,20α-THP, C										0.811	**	**									
3α,5α-THA/5α-DHA										0.032		*									
3α,5α-THAC/5α-DHA										0.171		**	0.444	*	*						
Explained variability, OPLS	0.414	*		0.683	**		0.867	**		0.536	**		0.440	*		0.579	**				

Table S2. Effects of treatment with individual anti-MS drugs and/or their groups on steroidome as evaluated by orthogonal projections to latent structures (OPLS) and ordinary multiple regression (OMR)

Variable	**GA** follicular phase			**IFNβ-1a** follicular phase			**IFNβ-1a** luteal phase			**S1PRI** follicular phase			**Ofatumumab** follicular phase			**Ocrelizumab** follicular phase			**Ocrelizumab** luteal phase		
	Component loading, OPLS	OPLS, p-value	OMR, p-value	Component loading, OPLS	OPLS, p-value	OMR, p-value	Component loading, OPLS	OPLS, p-value	OMR, p-value	Component loading, OPLS	OPLS, p-value	OMR, p-value	Component loading, OPLS	OPLS, p-value	OMR, p-value	Component loading, OPLS	OPLS, p-value	OMR, p-value	Component loading, OPLS	OPLS, p-value	OMR, p-value
**Subfamily 1C aldoketoreductase type 3 (AKR1C3) vs. 17β-hydroxysteroid dehydrogenase type 2 (HSD17B2)**																					
Adiol/DHEA																			-0.639	**	**
Adiol/DHEA, C													-0.599	**							
3β,7α,17β-AT/7α-OH-DHEA																			0.486		
3β,7β,17β-AT/7β-OH-DHEA													-0.549	*							
T/A																					
5α-DHT/5α-DHA																			-0.783	**	**
5α-DHTC/5α-DHA																					
3α,5α,17β-AD/3α,5α-THA																			-0.635	**	
3α,5α,17β-AD/3α,5α-THA, C													-0.823	**	*						
3β,5α,17β-AD/3β,5α-THA, C													-0.882	**	*						
3α,5β,17β-AD/3α,5β-THA, C																					
3β,5β,17β-AD/3β,5β-THA, C													-0.639	**							
Explained variability, OPLS													0.476	*					0.665	**	

**p<0.05, **p<0.01; the values in Table S2 represent the component loadings expressed as correlation coefficients with a predictive component (for detailed explanation see section Statistical analyses). The empty spaces in the Table represent lack of significance.*
